# Parasitaemia and fever in uncomplicated *Plasmodium vivax* malaria: A systematic review and individual patient data meta-analysis

**DOI:** 10.1371/journal.pntd.0012951

**Published:** 2025-03-28

**Authors:** Emily S. Groves, Julie A. Simpson, Peta Edler, André Daher, Ayodhia P. Pasaribu, Dhelio B. Pereira, Kavitha Saravu, Lorenz von Seidlein, Megha Rajasekhar, Ric N. Price, Robert J. Commons, on behalf of the WorldWide Antimalarial Resistance Network (WWARN) P. vivax Fever Study Group

**Affiliations:** 1 Nuffield Department of Medicine, Centre for Tropical Medicine and Global Health, University of Oxford, Oxford, United Kingdom; 2 WorldWide Antimalarial Resistance Network, Oxford, United Kingdom; 3 Global Health Division, Menzies School of Health Research and Charles Darwin University, Darwin, Northern Territory, Australia; 4 Centre for Epidemiology and Biostatistics, Melbourne School of Population and Global Health, The University of Melbourne, Melbourne, Victoria, Australia; 5 WorldWide Antimalarial Resistance Network, Asia-Pacific Regional Centre, Melbourne, Australia; 6 Department of Infectious Diseases, The University of Melbourne at the Peter Doherty Institute for Infection and Immunity, Melbourne, Victoria, Australia; 7 Fiocruz Clinical Research Platform, Oswaldo Cruz Foundation, Rio de Janeiro, Brazil; 8 Vice‑presidency of Research and Biological Collections, Oswaldo Cruz Foundation (FIOCRUZ), Rio de Janeiro, Brazil; 9 Department of Pediatrics, Medical Faculty, Universitas Sumatera Utara, Medan, North Sumatera, Indonesia; 10 Centro de Pesquisa em Medicina Tropical de Rondonia, Porto Velho, Brazil; 11 Fundação Universidade Federal de Rondonia, Porto Velho, Brazil; 12 Department of Infectious Diseases, Kasturba Medical College Manipal, Manipal Academy of Higher Education, Manipal, Karnataka, India; 13 Manipal Centre for Infectious Diseases, Prasanna School of Public Health, Manipal Academy of Higher Education, Manipal, Karnataka, India; 14 Mahidol Oxford Tropical Medicine Research Unit, Faculty of Tropical Medicine, Mahidol University, Bangkok, Thailand; 15 General and Subspecialty Medicine, Grampians Health - Ballarat, Ballarat, Victoria, Australia; Institut Pasteur, FRANCE

## Abstract

**Background:**

Parasite density thresholds used for diagnosing symptomatic malaria are defined by the relationship between parasitaemia and fever. This relationship can inform the design and development of novel diagnostic tests but appropriate parasitaemia thresholds for *Plasmodium vivax* malaria remain poorly defined.

**Methodology/principal findings:**

We undertook an individual patient data meta-analysis of *P. vivax* clinical trials mapped to the WorldWide Antimalarial Resistance Network (WWARN) repository and used parasitaemia centiles of febrile patients at enrolment to derive proportions of patients who would have been diagnosed at different parasite densities. Febrile and afebrile patients with recurrent infections were selected to estimate pyrogenic densities using receiver operating characteristic curve analysis. In total 13,263 patients from 50 studies were included in the analysis. In 27 studies (8,378 febrile patients) in which a parasitaemia threshold was not applied as an inclusion criterion, the median parasitaemia at enrolment was 3,280/µL (interquartile range, 968 – 8,320); 90% of patients had a parasitaemia above 278/µL (10^th^ centile), and 95% above 120/µL (5^th^ centile). The 10^th^ centile was higher in children <5 years old (368/µL) compared to adults ≥15 years (240/µL). In high relapse periodicity regions (Southeast Asia and Oceania) febrile patients presented with lower parasitaemias (10^th^ centile 185/µL vs. 504/µL) and a wider range of parasitaemias compared to those from low relapse periodicity regions (interquartile range 760/µL – 8,774/µL vs. 1,204/µL – 8,000/µL).

In total 2,270 patients from 41 studies had at least one episode of recurrent *P. vivax* parasitaemia, of whom 43% (849/1,983) were febrile at their first recurrence. The *P. vivax* pyrogenic density at first recurrence was 1,063/µL, defining fever with 74% sensitivity and 65% specificity. The pyrogenic density was lower in young children compared to adults ≥15 years (935/µL vs. 1,179/µL).

**Conclusions/significance:**

The derived parasitaemia centiles will inform the use of current and the design of novel point-of-care tests to diagnose patients with symptomatic vivax malaria. Variation by age and location should be considered when selecting diagnostic thresholds and interpreting results.

**Trial registration:**

This trial was registered with PROSPERO: CRD42021254905. The date of the first registration was 17th May 2021.

## Author summary

Rapid diagnostic tests (RDTs) offer an affordable and scalable method for diagnosing symptomatic malaria with reduced need for equipment, electricity, and training compared with light microscopy. These tests are particularly helpful in remote settings with the heaviest burden of malaria. However, appropriate parasite densities to accurately diagnose patients with symptomatic *Plasmodium vivax* malaria remain poorly defined. To inform the use of current and the design of novel RDTs for *P. vivax* malaria, we combined data from 50 clinical trials and used parasite concentrations in symptomatic patients at enrolment to determine the proportions of patients who would have been diagnosed at different parasitaemias. We found that detecting a parasitaemia >278/µL would diagnose 90% of patients presenting with a fever. Our analyses also explore how the *P. vivax* parasitaemia-fever relationship varies between regions and patient populations and include estimates of *P. vivax* pyrogenic densities at the first detected recurrent infection. These findings will inform *P. vivax* RDT use and development to support clinicians managing febrile patients in malaria endemic areas. The results highlight that while pyrogenic density estimates for recurrent *P. vivax* may be epidemiologically informative, they have relatively low sensitivities for defining symptomatic malaria.

## Introduction

*Plasmodium vivax* causes an estimated 4–14 million cases of malaria per year, reported from 45 endemic countries across the Asia-Pacific, the Horn of Africa, and the Americas [1,2]. Relapsing infections result from activation of dormant liver stages (hypnozoites) and can cause anaemia and substantial morbidity, particularly in children and pregnant women [3,4].

Optimal malaria control activities differ between endemic settings according to a variety of factors including the burden of malaria and transmission intensity. In high transmission areas, the priority is early diagnosis and treatment of patients with clinical disease, the majority of whom are young children [5]. The main burden of malaria is in remote and poorly resourced areas, where there is a critical need for rapid, accessible, affordable, and easy-to-use diagnostic tools. Conversely in low endemicity settings, where elimination strategies are being considered, an additional focus is to identify and kill parasite reservoirs; this includes active detection of individuals with both symptomatic and asymptomatic infection [6].

Rapid diagnostic tests (RDTs) are used routinely in the case management of patients with *P. falciparum* malaria, and this increases the proportion of patients with confirmed infection prior to administration of antimalarial treatment. RDTs have potential to facilitate parasite surveillance, reduce the time to diagnosis and treatment, and rationalise the use of antimalarial drugs [6]. The development of accurate RDTs for *P. vivax* is more challenging than for *P. falciparum,* a consequence of lower peripheral parasitaemias, different parasite antigens, and a smaller global burden compared to *P. falciparum* [7,8]. Development of high-quality *P. vivax*-detecting RDTs has therefore been slower than for *P. falciparum*, although diagnostic performance has improved over time [9].

RDTs are simple to use but only provide a binary readout, in contrast to light microscopy which quantifies the peripheral blood parasite density. The pyrogenic threshold of a *Plasmodium* infection is defined as the peripheral blood parasite density required to induce a fever. It is not a fixed entity and varies substantially both within individuals and populations depending on a wide variety of inter-related factors [10]. These include parasite species and strain, and factors related to exposure to previous infection (immunity), including patient age, occupation, and transmission intensity, which vary by geographic location and season in areas of seasonal transmission. To interpret pyrogenic density estimates it is therefore important to understand the context in which and the methods by which they were derived.

The most rigorous method for estimating a patient’s pyrogenic density is to measure their parasitaemia and temperature at frequent intervals during a *Plasmodium* infection to quantify the parasitaemia at the precise onset of fever, as performed in the *P. vivax* malaria therapy studies conducted in the first half of the 20^th^ century [11]. However, this approach is generally impracticable at both individual and population levels. Instead, various mathematical modelling and statistical methods have been employed to estimate pyrogenic densities, almost all in the context of *P. falciparum* [10,12–14].

Context-specific population pyrogenic density estimates are valuable both clinically and epidemiologically. Knowledge of whether the parasite density exceeds the pyrogenic threshold can aid clinical decision-making about the probability that a fever is due to malaria. This is particularly important for *P. falciparum* in highly endemic areas where peripheral parasitaemia may be incidental to other causes of fever. As peripheral parasitaemia decreases the pre-test probability of concurrent malarial fever reduces, thus, in patients with lower parasitaemia, alternative causes of febrile illness should be considered, such as sepsis requiring antibiotic treatment. It is also possible that symptomatic malaria may occasionally occur in non-immune patients who have a rising (but as yet undetectable) parasitaemia [15]. *P. vivax* population pyrogenic density estimates can be informative from an epidemiological perspective, with their variation over time informing the assessment of disease burden and the identification of populations where asymptomatic transmission is likely to be high [13]. They can therefore be helpful for targeting malaria control and elimination strategies.

We undertook a systematic review and individual patient data (IPD) meta-analysis of patients enrolled into prospective clinical efficacy studies with *P. vivax* parasitaemia and investigated the relationship between parasitaemia and fever to inform *P. vivax* RDT target product profiles and estimate pyrogenic densities at the first recurrence.

## Methods

### Search strategy and selection criteria

Studies were identified from an ongoing systematic review registered in PROSPERO according to the Preferred Reporting Items for Systematic Reviews and Meta-Analyses (PRISMA) statement which was updated as of 16^th^ February 2021 [16] (Checklist A in S1 Text). MEDLINE, Web of Science, Embase, and Cochrane Central were searched to identify prospective clinical efficacy studies of uncomplicated *P. vivax* mono-infection published in any language between 1^st^ January 2000 and 16^th^ February 2021. Search terms and criteria are presented in Box A in S1 Text. Investigators of eligible *P. vivax* prospective clinical efficacy studies published since 2000 identified by the systematic review were invited to share their data and any additional unpublished data to the WorldWide Antimalarial Resistance Network (WWARN) data repository for previous and planned individual patient data meta-analyses [17–21].

Eligible studies were included in the analysis if they fulfilled the following criteria: mapped to the WWARN repository by 16^th^ February 2021; patients were followed for a minimum of 28 days; patients were treated with chloroquine (CQ) or an artemisinin-based combination therapy (ACT) without an adjunctive schizonticidal drug; and data were available on the following variables: date of enrolment; age and sex of participants; the asexual parasite density based on blood film microscopy for each episode of *P. vivax* parasitaemia; objective or subjective fever information; and timing of recurrent parasitaemia in relation to the date of enrolment. Patients treated with CQ or an ACT plus a primarily hypnozoitocidal drug were included. A Data Access Request was submitted to WWARN and permission was sought from the investigators or sponsors to re-use eligible data. Data held in the repository are pseudonymised, curated, and standardised according to a predefined data management plan to produce a single dataset [22].

All studies included in the analysis had been granted ethical approvals from their countries of origin. Data were pseudonymised, de-identified, and could not be traced to original participants. This analysis did not require additional ethics committee review according to Oxford Central University Research Ethics Committee guidelines.

### Procedures

Patients were excluded from the analysis if they had any of the following criteria: i) no documentation of presence of *P. vivax* parasitaemia on day 0, ii) mixed species infection detected on day 0 or 1, iii) presented with severe malaria, an enrolment deviation, or were pregnant. The presenting and recurrent episodes of *P. vivax* infection were excluded if febrile status or baseline parasitaemia were unavailable. Patients with mixed species infection or severe malaria at the time of recurrence were excluded from the recurrent episode analysis.

Patients were classified as febrile if they had a documented fever or recent history of fever at presentation or recurrence. Fever was defined as an axillary temperature ≥37.5°C or tympanic, oral, or rectal temperature ≥38.0°C. If the method of temperature measurement was not recorded, fever was defined as a temperature of ≥37.5°C (7 studies). Recent history of fever was defined as participant-reported fever within the preceding 72 hours. If there was no information about fever at study enrolment, the participant was assumed to be febrile if fever or recent history of fever were part of the study inclusion criteria.

The first recurrence was defined as the first episode of microscopy-confirmed *P. vivax* parasitaemia occurring >7 days after initiation of antimalarial treatment, with an intervening negative smear. Subsequent recurrences were defined according to the same criteria, occurring >7 days after the preceding recurrence with an intervening negative smear.

Parasitaemia centiles were defined as the percentage of febrile patients with *P. vivax* parasitaemia below that parasitaemia. Age groups were categorised as <5 years, 5 to <15 years, and ≥15 years. Regional relapse periodicity was categorised as high (short) or low (long) according to geographical area [23]. High periodicity regions had a median time to patent relapse of ≤47 days. Transmission intensity was categorised as low (≤1 case per 1000 people per year), moderate (>1 to ≤10 cases per 1000 people per year), or high (>10 cases per 1000 people per year) according to malaria incidence rate estimates obtained for subnational regions from the Malaria Atlas Project using the median year of study enrolment [24]. Elimination half-life of schizonticidal treatment was categorised as rapid (<1 day), intermediate (1–7 days), or slow (>7 days) and combination therapies were categorised based on the drug with the longest elimination half-life [25] (Table A in S1 Text).

### Outcomes

The primary outcome was the 10^th^ centile of *P. vivax* parasitaemia in febrile patients presenting for treatment at enrolment. Secondary outcomes were the 25^th^, 5^th^, and 1^st^ centiles for febrile patients on day 0; 25^th^, 10^th^, 5^th^, and 1^st^ centiles for febrile and afebrile patients at the first recorded recurrent infection; and *P. vivax* pyrogenic density at the first recorded recurrent infection. Pyrogenic density is defined as the peripheral parasitaemia required to induce a febrile illness.

### Statistical analysis

At presentation (day 0) the distribution of parasitaemia for febrile patients enrolled into all studies that did not apply a parasitaemia threshold for enrolment was examined to determine the median, interquartile range (IQR), and parasitaemia centiles. A sensitivity analysis compared these parasitaemia centiles to those calculated for studies which estimated parasite density by the same method (parasite count per number of white blood cells (WBCs) and assumed a WBC count of 5,500-8,000/µL) (Table B in S1 Text). Subgroup analyses were performed for each age group, relapse periodicity category, as well as age groups within each relapse periodicity category. Analyses were repeated for the first recurrent episode in febrile and afebrile patients enrolled in studies in which participants experienced at least one recurrence, regardless of whether a parasitaemia threshold was applied at enrolment.

Receiver operating characteristic (ROC) curves were constructed and Youden’s index calculated to identify parasite density values with optimal sensitivity and specificity for defining the pyrogenic threshold for the first recurrence, overall and by age group and relapse periodicity.

Univariable logistic regression analysis was used to define the relationship between fever and *P. vivax* parasitaemia at first recurrence. Mixed effects multivariable logistic regression analysis was then used to adjust for age group, sex, relapse periodicity, half-life of schizonticidal treatment, and timing of recurrence (before or after day 90), with random effects for study site.

To assess for bias related to individual studies for either the enrolment parasitaemia centiles or pyrogenic density analyses, study sites were removed one at a time and the coefficient of variation (CV) around the parameter estimates recalculated to assess heterogeneity (Table C in S1 Text). Baseline characteristics of included studies were also compared to targeted studies which were not included (Table D in S1 Text).

Statistical analyses were conducted in Stata (version 17.0) as per an a-priori statistical analysis plan [26]. The review protocol is registered in PROSPERO, number CRD42021254905 [27].

## Results

**Fig 1:**
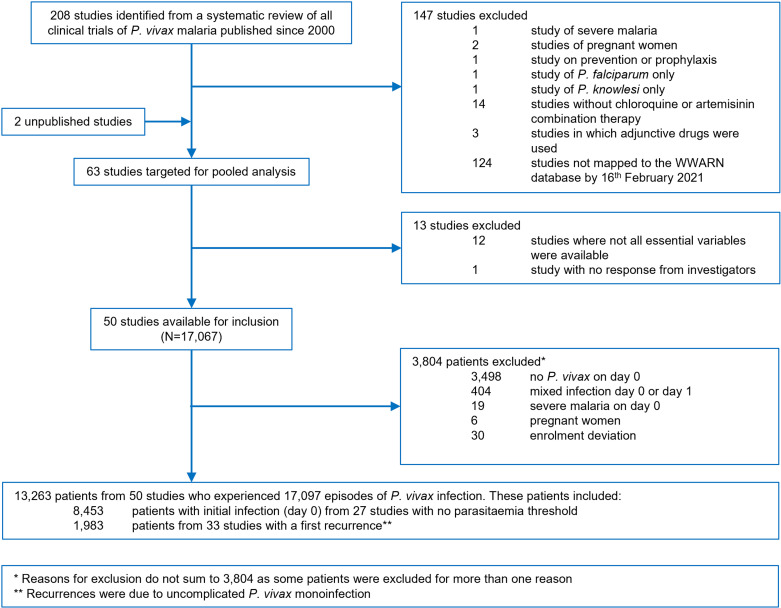
Study and patient flow diagram.

Between 1st January 2000 and 16th February 2021, a total of 208 *P. vivax* clinical efficacy studies were identified of which 23 were excluded due to not satisfying the inclusion and exclusion criteria. An additional 124 studies were excluded as they were not mapped to the WWARN repository by 16th February 2021 and 13 mapped studies were excluded due to missing essential variables or being unable to confirm inclusion with the investigators (Fig 1 and Tables E and F in S1 Text). Individual patient data from 48 (23.2%) published studies and 2 unpublished studies were included in the analysis (Tables G and H in S1 Text). Of the 17,067 patients with data available, 3,804 (22.3%) were excluded since patients presented with *P. vivax* mixed infections, severe malaria, pregnancy, or had at least one other enrolment deviation. In total 13,263 patients from 50 studies were included in the final analysis (Fig 1). Patients in included studies had similar baseline characteristics to those in studies targeted for the analysis (Table D in S1 Text).

### Parasitaemia centiles for febrile patients on day of enrolment (passive case detection)

In 23 (46%) studies (4,799 patients), a minimum parasite density was used to determine eligibility of patients for enrolment; these studies were excluded from the analysis of parasitaemia centiles. Of the remaining 8,464 patients, 11 were excluded because their febrile status was not recorded. Of 8,453 patients from 27 studies, 7,253 (85.8%) were enrolled in the Asia-Pacific (13 countries), 189 (2.2%) in the Americas (1 country), and 1,011 (12.0%) in Africa (2 countries) (Table 1). The median age of patients was 16.5 years (IQR 9.0–28.0, range 0.3–94.3), with 605 (7.2%) aged <5 years, 3,073 (36.4%) aged 5–<15 years, and 4,775 (56.5%) aged ≥15 years old. Overall, 4,969 (58.8%) were from high relapse periodicity regions compared to 3,484 (41.2%) from low relapse periodicity regions. The origin of patients was equally divided between settings of low (2,629, 31.1%), moderate (2,987, 35.3%) and high (2,776, 32.8%) transmission intensity (Table 1).

**Table 1:** Baseline demographics and characteristics.

Variable	Enrolment (day 0) patient cohort (N = 8 453) n (%)^a^		First recurrence patient cohort with fever (N = 849) n (%)^a^		First recurrence patient cohort without fever (N = 1 134) n (%)^a^	
**Studies, n (%)**	27	(54.0%)	29	(58.0%)	31	(62.0%)
**Sex**						
Male	5,148	(60.9%)	552	(65.0%)	730	(64.4%)
Female	3,305	(39.1%)	297	(35.0%)	404	(35.6%)
**Age (years)**						
Median (IQR)	16.5 (9.0–28.0)		16.0 (8.0–27.0)		15.0 (6.0–25.0)	
<5	605	(7.16%)	103	(12.1%)	227	(20.0%)
5 to <15	3,073	(36.4%)	280	(33.0%)	324	(28.6%)
≥15	4,775	(56.5%)	466	(54.9%)	583	(51.4%)
**Geographical region**						
Asia-Pacific	7,253	(85.8%)	570	(67.1%)	970	(85.5%)
The Americas	189	(2.24%)	29	(3.42%)	46	(4.06%)
Africa	1,011	(12.0%)	250	(29.5%)	118	(10.4%)
**Relapse periodicity**						
Low	3,484	(41.2%)	360	(42.4%)	237	(20.9%)
High	4,969	(58.8%)	489	(57.6%)	897	(79.1%)
**Transmission intensity**						
Low	2,629	(31.1%)	254	(29.9%)	119	(10.5%)
Moderate	2,987	(35.3%)	249	(29.3%)	599	(52.8%)
High	2,776	(32.8%)	345	(40.6%)	407	(35.9%)
Unavailable	61	(0.72%)	1	(0.12%)	9	(0.79%)
**Fever or recent history of fever**						
Yes	8,378	(99.1%)	–		–	
No	75	(0.89%)	–		–	
**Parasitaemia (parasites per µL), Median (IQR)**						
Fever	3,280 (968–8,320)		3,926 (1,019–9,744)		–	
No Fever	375 (188–2,336)		–		434 (93–2,480)	

^a^Unless otherwise specified

**Fig 2:**
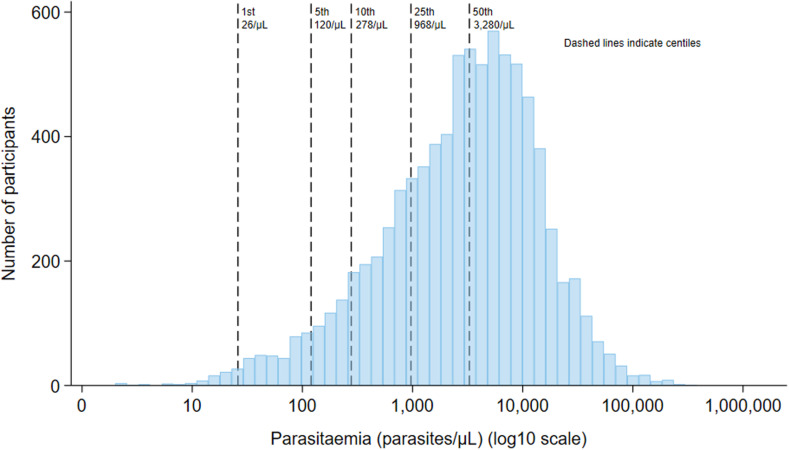
Distribution of parasite density at enrolment in febrile participants presenting with *P. vivax* malaria. The dashed lines indicate the 1^st^, 5^th^, 10^th^, 25^th^ and 50^th^ parasitaemia centiles. Includes the 99% (8,378/8,453) of participants enrolled into 27 clinical efficacy studies who were symptomatic with a fever or history of fever.

**Fig 3:**
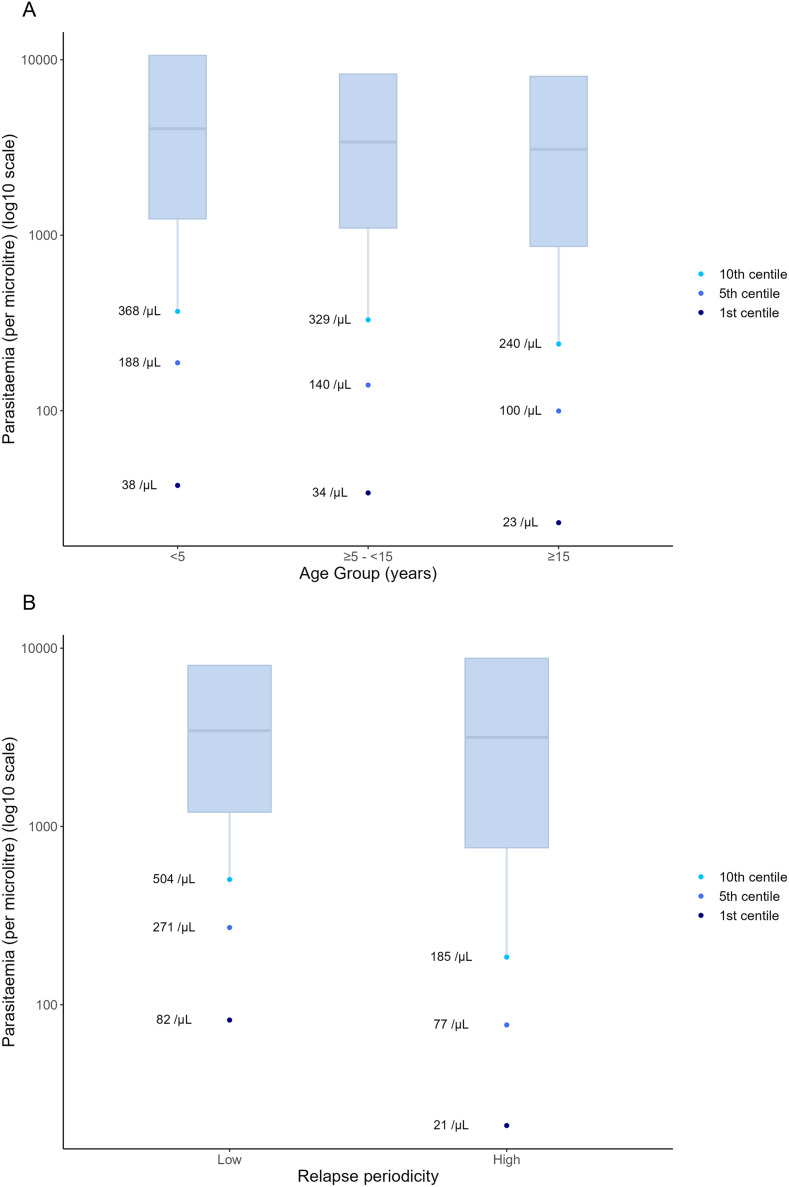
Parasitaemia centiles for febrile participants at enrolment by A) age group (years) and B) relapse periodicity region. The boxes describe the 25^th^-75^th^ parasitaemia centiles with the 50^th^ centile at the internal line.

Fever or history of fever was present at enrolment in 99% (8,378/8,453) of patients. The median parasite density (50th centile) was 3,280/µL (IQR 968–8,320) for febrile patients compared to 375/µL (IQR 188–2,336) for the 75 afebrile patients. The 25^th^, 10^th^, 5^th^, and 1^st^ parasitaemia centiles for febrile patients were 968/µL, 278/µL, 120/µL, and 26/µL, respectively (Fig 2). In a sensitivity analysis removing one study site at a time there was no evidence of bias relating to individual study sites (Table C in S1 Text). Parasitaemia centiles were similar in a sensitivity analysis which included 12 studies (3,009 patients) which calculated parasite density by the same method (Table B in S1 Text). Parasite densities varied with age. Compared to febrile patients older than 15 years, febrile children presented with higher parasitaemias with a wider interquartile range (IQR) (Fig 3 and Table I in S1 Text). The 10^th^ parasitaemia centile for febrile patients was 368/µL in children <5 years old compared to 329/µL for children aged 5 to <15 years and 240/µL for patients aged ≥15 years. There was substantial heterogeneity between studies (Figs A and B in S1 Text). In high relapse periodicity regions parasitaemia centiles at enrolment were lower and the IQR wider compared to patients enrolled in low relapse periodicity regions (10^th^ centile 185/µL vs. 504/µL) (Fig 3 and Table I in S1 Text).

**Fig 4:**
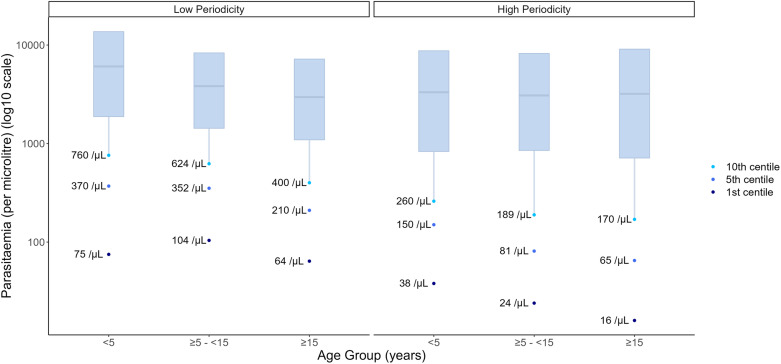
Parasitaemia centiles for febrile participants at enrolment by relapse periodicity region and age group (years). The box describes the 25^th^-75^th^ parasitaemia centiles with the 50^th^ centile at the internal line.

Parasitaemia centiles were lower for each age group in high compared to low relapse periodicity regions (Fig 4 and Table J in S1 Text). Children had higher parasitaemia centiles than people ≥15 years in both settings, and this trend was more pronounced in low relapse periodicity areas.

### Parasite density and fever at the time of first recurrence (active and passive case detection)

**Fig 5:**
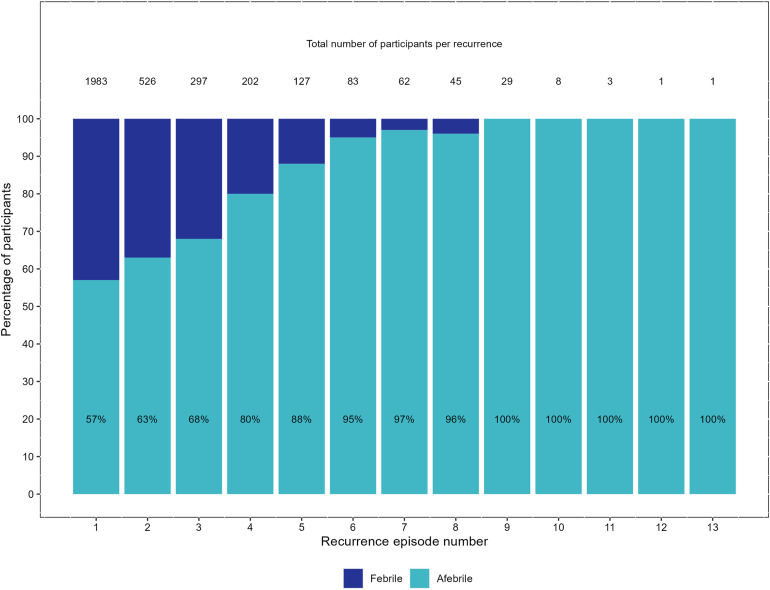
Percentage of participants with and without fever at the time recurrent parasitaemia was observed.

Overall, 2,270 patients (41 studies) experienced at least one episode of recurrent *P. vivax* parasitaemia during follow up. Study follow up ranged from 28 days to 2 years and the total number of recorded recurrences ranged from 1 to 13. The proportion of recurrences associated with fever decreased with an increasing number of recurrences per patient (Fig 5).

**Fig 6:**
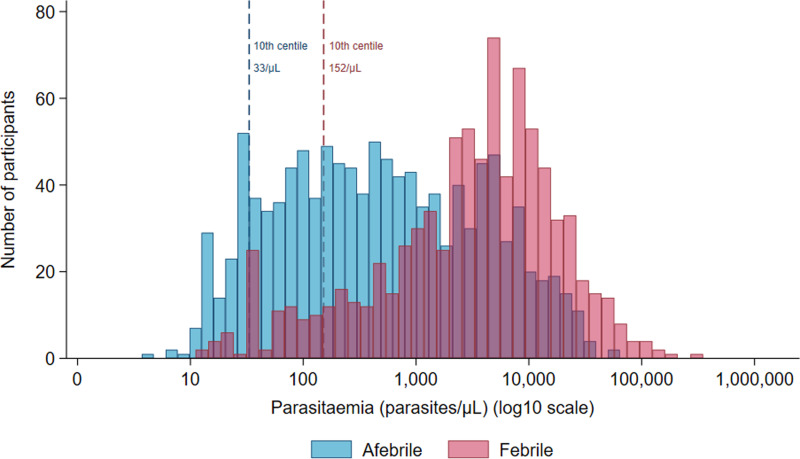
Parasite density distribution by fever status at first *P. vivax* recurrence.

At the first recurrence, data on both parasitaemia and fever were recorded for 1,983 (87.4%) patients enrolled into 33 studies. The median time to first recurrence was 46 days (range 8 to 371), with 43% (849/1,983) of patients febrile at the time of detection of recurrence (Fig 5). Overall, 31.2% (103/330) of children <5 years old were febrile compared to 46.4% (280/604) of older children (5 to <15 years old), and 44.4% (466/1,049) of people ≥15 years. The median parasite density was 3,926/µL (IQR 1,019 – 9,744) in the 849 febrile patients compared to 434/µL (IQR 93 – 2,480) in the 1,134 afebrile patients (Fig 6). The parasitaemia centiles for febrile and afebrile patients at first recurrent *P. vivax* episode are presented in Table I in S1 Text.

Of the 849 patients with fever at the time of their first recurrence, 23% (196/849) had a second recorded recurrence of whom 62% (121/196) were febrile (median parasite density 5,880/µL [IQR 2,211 – 12,500]), and 38% (74/196) were afebrile (median parasite density 536/µL [IQR 136 – 3074]). One patient’s fever status was not recorded.

### Pyrogenic densities at the first recorded recurrence (active and passive case detection)

At the time of their first recorded *P. vivax* recurrence, 43% (849/1,983) of patients were febrile. There was a greater odds of fever in patients with higher parasitaemia (Odds Ratio (OR) 2.77 [95%CI 2.00-3.84] for every 10-fold increase in peripheral parasitaemia; p<0.0001). The estimated *P. vivax* pyrogenic density for patients at their first recorded recurrence was 1,063/µL, and at this threshold the sensitivity for detecting a patient with documented fever was 74% [95%CI 71-77%], with a specificity of 65% [95%CI 62-67%] (Fig C in S1 Text). The estimated pyrogenic density was 935/µL in children <5 years old (n=330; sensitivity 76%, specificity 61%) compared with 1,915/µL in children 5-<15 years old (n=604; sensitivity 69%, specificity 75%) and 1,179/µL in people ≥15 years (n=1,049; sensitivity 74%, specificity 65%). The estimated pyrogenic density was 930/µL in low relapse periodicity areas (sensitivity 84%, specificity 51%) where 60% (360/597) of patients had fever at first detected recurrence, and 1,743/µL in high relapse periodicity settings (sensitivity 65%, specificity 73%) where 35% (489/1,386) of patients had fever at first detected recurrence.

Patients residing in high relapse periodicity areas had a lower risk of fever at first recurrence than those in low relapse periodicity regions (OR 0.36 [95%CI 0.14-0.90]; p=0.029), as did patients treated with rapidly-eliminated compared to slowly-eliminated schizonticidal antimalarials (OR 0.18 [95%CI 0.11-0.29]; p<0.0001), and patients whose first recurrence occurred within 90 days of the initial episode (OR 0.41 [95%CI 0.24-0.70]; p=0.001) (Table K in S1 Text). The median time to first recurrence was 28 days (range 14-252 days) for rapidly-eliminated antimalarials, compared to 35 days (range 14-293 days) for intermediately-eliminated antimalarials and 49 days (range 8-371 days) for slowly-eliminated antimalarials. After adjusting for confounding factors, the Adjusted Odds Ratio (AOR) for fever at first recurrence was 2.62 [95%CI 1.98 – 3.47] for every 10-fold increase in parasitaemia; p<0.0001.

## Discussion

Our analysis derived parasitaemia centiles for patients presenting with vivax malaria across diverse populations. Overall parasitaemias greater than 278/µL and 120/µL were present in 90% and 95% of febrile patients, respectively. Diagnostic tests capable of detecting these levels of parasitaemia would thus identify the majority of patients with symptomatic *P. vivax* malaria. There were significant differences between sites and patient populations, with parasitaemia centiles lower in older age groups (10^th^ centile 240/µL in adults versus >300/µL in both groups of children) and areas with high relapse periodicity (10^th^ centile 185/µL versus 504/µL in low relapse periodicity settings). These findings could inform target product profiles for novel and existing RDTs and guide National Malaria Programmes (NMPs) when selecting RDTs to ensure diagnostic detection thresholds are appropriate for local endemic settings. At the first recurrence, the estimated *P. vivax* pyrogenic density was 1,063/µL overall, and was almost two-fold higher in high versus low relapse periodicity regions and ~1.25-fold higher in patients ≥15 years old compared to young children <5 years old.

Parasitological confirmation of malaria prior to administration of treatment improves clinical outcomes and rationalises the use of antimalarial drugs [6,28]. Light microscopy remains the gold standard diagnostic test for symptomatic malaria, however, it is not universally available and is subject to interindividual variability [29]. Malaria RDTs offer an affordable alternative and are critical both for case management and progress towards elimination [30]. In 2023, approximately 130 million *P. falciparum*/*P. vivax* combination RDTs were sold by manufacturers to NMPs, approximately half of which (~65 million) were sold to NMPs outside sub-Saharan Africa [1]. The use of RDTs at the community level for diagnosing malaria is recommended as part of national policy in 82% (37/45) of *P. vivax* endemic countries, of which 78% (29/37) implemented this policy in 2023 [1]. World Health Organization (WHO) pre-qualification for RDTs requires satisfactory laboratory performance against diagnostic thresholds of 200/µL and 2000/µL for *P. vivax* [31]. Our individual patient data pooled meta-analysis suggests that the lower proposed threshold of 200/µL would detect ~92% of patients with symptomatic vivax malaria. There are currently 13 WHO-prequalified RDTs capable of detecting *P. vivax*, all of which are combination RDTs and 8 of which detect *P. vivax*-specific pLDH (the remaining 5 detect pan-species pLDH) [32]. The pooled sensitivity and specificity of the CareStart Pf/Pv RDT for *P. vivax* from 4 studies were 99% and 99% compared to microscopy, and the pooled sensitivity and specificity of the Falcivax RDT from 2 studies for *P. vivax* were 77% and 99% respectively. However, further high-quality, independent studies conducted in endemic areas are needed to evaluate the real-world performance of existing RDTs for diagnosing *P. vivax* malaria [7].

In 1886, Golgi showed that malarial fever is caused by sporulation of the *Plasmodium* – the rupture of mature erythrocytic schizonts in the circulation. Rupture results in the release of merozoites and erythrocyte and parasite debris, including malarial pigment (haemozoin) and glycophosphatidylinositol, the putative “malaria toxin” [33]; the temperature returns to normal over several hours. Subsequent merozoites undergo asexual reproduction, and schizont rupture recurs [34]. Once an infected individual becomes symptomatic and fever persists they are more likely to present for medical assistance. Our pooled meta-analysis used data from febrile patients who presented to healthcare facilities and were enrolled into antimalarial clinical trials. The parasitaemia at the time of presentation is determined by a variety of factors including transmission intensity, patient age, innate and acquired immunity (including the ability to tolerate parasitaemia without symptoms), access to healthcare, and treatment-seeking behaviour [35,36]. Repeated episodes of malaria result in the development of pathogen-specific host immunity, allowing some individuals to harbour higher parasitaemias without manifesting symptoms (anti-disease immunity) [11]. Acquired immunity may also lead to suppression of parasitaemia on re-exposure (anti-parasite immunity) [37]. In patients with *P. falciparum* malaria, increasing age and transmission intensity enhance both types of immunity [38]. In our study, parasitaemia centiles were lower in patients ≥15 years old and those living in high relapse regions, potentially consistent with anti-parasite immunity restricting the ability of the parasite to replicate and suppressing parasitaemia. Similarly, in low endemic areas of the Peruvian Amazon, adults with *P. vivax* infection were more likely to have symptoms at lower parasite densities compared to young children [39].

Our estimates of the pyrogenic density at the first recurrence were almost two-fold higher in high (1,743/µL) versus low (930/µL) relapse periodicity areas. The pyrogenic density was lowest in young children (935/µL), highest in older children (1,915/µL), but fell in adults (1,179/µL). This may reflect the increased burden of infection in older children on a background of increasing immunity. There were insufficient numbers of patients to estimate the pyrogenic density for each age group by relapse periodicity. These estimates were derived from a specific group of patients who had recently been diagnosed and treated for symptomatic *P. vivax* malaria and whose recurrent parasitaemia was identified through both active and passive follow-up. However, this is a highly relevant group of patients, since ~80% of all *P. vivax* infections in endemic settings are estimated to be due to relapses [40]. The relative differences according to age and relapse periodicity are likely informative, but the absolute numbers should be interpreted with our particular study population in mind.

Pyrogenic thresholds have been studied extensively in patients with falciparum malaria. In holoendemic areas of sub-Saharan Africa, young children have the highest pyrogenic density, attributable to persistent high parasite loads maintaining high levels of anti-toxin antibodies [14]. With increasing age, anti-parasite immunity suppresses parasite growth and resulting parasite loads are insufficient to maintain anti-toxin antibodies, hence fever tends to develop at lower parasitaemias. The variation of the *P. vivax* pyrogenic density with age and endemicity has been less studied. In a low transmission setting on the western border of Thailand, the pyrogenic density in children aged 4-15 years was estimated to be 181/µL for *P. vivax* compared with 1,460/µL for *P. falciparum* (determined from community cross-sectional surveys by calculating the geometric mean parasite density at the onset of symptoms) [41]. We estimated population pyrogenic densities by comparing the parasitaemias of febrile and afebrile patients at the first detected recurrence after a recently diagnosed and treated episode of symptomatic malaria (recurrence pyrogenic density). Cross-sectional surveys of populations comparing the parasite densities of febrile and afebrile patients in different community settings would provide more accurate assessments of overall population pyrogenic densities and their variation with sociodemographic and epidemiological factors.

Estimating pyrogenic densities at the population level is different from determining pyrogenic densities at the individual patient level as performed in the malaria therapy studies conducted prior to 1950. Estimates were determined from the onset of fever in deliberately infected individuals [11]. In 1910, Ross observed that non-immune patients generally developed a fever at a *P. vivax* parasite density of ~50/µL; while Kitchen found the pyrogenic density to be 10 to 120/µL in 4 non-immune patients; and Sinton found the pyrogenic density to be 200-250/µL in “early” cases and ~5000/µL in “chronic” vivax infections [34,42,43]. In 1938, Boyd showed that vivax pyrogenic density varied substantially depending on host immunity where, similar to our study, several thousand parasites/µL were required to induce fever in patients with prior exposure to infection [44]. The clinical decision to prescribe antimalarial drugs for a patient is generally based on the detection of any parasites in the blood, irrespective of the parasite density. Therefore, rather than influencing decisions about whether to administer antimalarial treatment, knowledge of location and age-dependent pyrogenic densities informs whether a fever is likely to be attributable to malaria and thus the need to investigate for and treat other causes of fever. The relationship between parasitaemia and risk of attributable disease is similar between *Plasmodium* species [45]. Estimates can also provide valuable epidemiological information about disease burden and where high levels of asymptomatic transmission are likely to be occurring to help target malaria control and elimination activities.

To our knowledge, this is the first systematic review and meta-analysis investigating the relationship between parasitaemia and fever in *P. vivax*. However, our study has a number of limitations. The geographical generalisability of our findings is limited since 86% of participants included in the day 0 analysis were enrolled in studies conducted in the Asia-Pacific region. Studies were only included if they had been mapped to the WWARN database prior to 16^th^ February 2021, excluding 124 potentially suitable studies, 65% (81/124) of which were conducted in the Asia-Pacific, 25% (31/124) in the Americas, 8% (10/124) in Africa, and 2% (2/124) in multiple regions. However, the sites included in the analysis represent a wide variety of endemic settings and thus our findings are likely to be generalisable to similar settings in the Americas and Africa. Furthermore, although studies were only included if enrolment did not include a parasitaemia threshold, all studies used light microscopy to quantify parasitaemia, placing an inadvertent threshold on quantifying parasitaemia to approximately 25/µL [46]. However, this value is consistent with *P. vivax* pyrogenic densities estimated in individuals during malaria therapy studies [34]. Almost all patients at enrolment presented with either fever or a history of fever, thus population pyrogenic densities could only be estimated during follow up, during which *P. vivax* recurrences were actively detected in both febrile and afebrile individuals. Recurrent parasitaemia was detected through active detection at study-defined timepoints and passive detection when individuals became symptomatic; this approach will have missed individuals with asymptomatic recurrences occurring between routine follow up visits, underestimating the proportion of patients with afebrile parasitaemia at first recurrence and confounding the analysis of risk factors associated with fever.

The parasitaemia reported depends on the method of quantification used in blood film examination by light microscopy [47]. To account for this, we undertook a sensitivity analysis restricted to 12 studies (3,009 patients) which used a similar method of estimating parasite density – the overall centiles at enrolment were similar (Table B in S1 Text). Microscopy quality assurance information was available for 80% (40/50) of all included studies and for 78% (21/27) of studies included in the day 0 analysis (Table G in S1 Text). Of the 40 studies that provided information on quality assurance, all slides were examined by two or more independent microscopists in 75% (30/40) of cases. Our analysis assumed that patients enrolled in the original studies presented with an initial infection, however, they may have presented with a *P. vivax* relapse. Finally, current *P. vivax* RDTs detect parasite antigen concentration (*Plasmodium* lactate dehydrogenase, pLDH) rather than parasite density. However, pLDH concentration and parasitaemia have been shown to be well-correlated [48,49] and pLDH concentration may correlate with the total body *P. vivax* parasite biomass more closely than peripheral parasitaemia due to sequestration of parasites in the spleen [50–52].

In conclusion, the derived parasitaemia centiles from febrile patients presenting with *P. vivax* malaria will inform clinical diagnosis and target product profiles of RDTs. Estimates of pyrogenic density may help clinicians contextualise case management and act as surrogate markers of regional endemicity and population immunity, however they are less informative for assessing the utility and performance of novel diagnostic tests due to their relatively low sensitivities for defining symptomatic malaria. More sensitive diagnostics are required in regions moving towards malaria elimination where low density asymptomatic infections predominate and act as an important residual driver of onward transmission [39,53,54]

## Supporting information

S1 Text Supplementary information presented as checklists, boxes, tables, and figures. (DOCX)

S1 Acknowledgments Membership of the WWARN *P. vivax* Fever Study Group. (DOCX)
