# Supplementary material for: Parasitaemia and fever in uncomplicated Plasmodium vivax malaria: A systematic review and individual patient data meta-analysis
Source: PLoS Negl Trop Dis. 2025 Mar 28;19(3):e0012951. doi: 10.1371/journal.pntd.0012951 (PMC11978046; doi:10.1371/journal.pntd.0012951)
Supplement: S1 Text — (DOCX) [file pntd.0012951.s001.docx]

**Supporting Information**

**Contents**

[**Checklist A:** PRISMA-IPD Checklist for reporting a systematic review and meta-analysis of individual participant data (IPD) **2**](#_Toc191819250)

[**Box A:** Search strategy **6**](#_Toc191819251)

[**Table A:** Schizonticidal treatments administered A) classified by elimination half-life and B) as treatment arms categorised by elimination half-life **7**](#_Toc191819252)

[**Table B:** Sensitivity analysis comparing enrolment parasitaemia centiles for febrile patients **8**](#_Toc191819253)

[**Table C:** Sensitivity analyses for enrolment parasitaemia centiles and estimated pyrogenic density at the first recurrence **8**](#_Toc191819254)

[**Table D:** Comparison of baseline characteristics between included and targeted studies **9**](#_Toc191819255)

[**Table E:** Reasons for study exclusion **9**](#_Toc191819256)

[**Table F:** Studies excluded from the analysis **10**](#_Toc191819257)

[**Table G:** Studies included in the analysis **16**](#_Toc191819258)

[**Table H:** Study sites included in the analysis **21**](#_Toc191819259)

[**Table I:** Parasite density distributions and centiles at enrolment and first recurrence by fever status **24**](#_Toc191819260)

[**Figure A:** Parasite density distributions at enrolment for each study by age group and relapse periodicity **25**](#_Toc191819261)

[**Figure B:** Parasite density distributions at enrolment for each study site by age group and relapse periodicity **26**](#_Toc191819262)

[**Table J:** Parasite density distributions and centiles for febrile patients at enrolment by relapse periodicity and age **27**](#_Toc191819263)

[**Figure C:** Receiver operating characteristic curve depicting three thresholds (parasites/µL) including the pyrogenic density at first recurrence **28**](#_Toc191819264)

[**Table K:** Risk factors for fever at first *P. vivax* parasite recurrence **29**](#_Toc191819265)

[**References** **30**](#_Toc191819266)

Checklist A: PRISMA-IPD Checklist for reporting a systematic review and meta-analysis of individual participant data (IPD)

| **PRISMA-IPD**  **Section/topic** | **Item No** | **Checklist item** | **Reported on page** |
| --- | --- | --- | --- |
| **Title** | | | |
| Title | 1 | Identify the report as a systematic review and meta-analysis of individual participant data. | 1 |
| **Abstract** | | | |
| Structured summary | 2 | Provide a structured summary including as applicable: | 2  2  2-3  3  9, 16-17 |
|  |  | **Background**: state research question and main objectives, with information on participants, interventions, comparators and outcomes. |  |
|  |  | **Methods**: report eligibility criteria; data sources including dates of last bibliographic search or elicitation, noting that IPD were sought; methods of assessing risk of bias. |  |
|  |  | **Results**: provide number and type of studies and participants identified and number (%) obtained; summary effect estimates for main outcomes (benefits and harms) with confidence intervals and measures of statistical heterogeneity. Describe the direction and size of summary effects in terms meaningful to those who would put findings into practice. |  |
|  |  | **Discussion:** state main strengths and limitations of the evidence, general interpretation of the results and any important implications. |  |
|  |  | **Other:** report primary funding source, registration number and registry name for the systematic review and IPD meta-analysis. |  |
| **Introduction** | | | |
| Rationale | 3 | Describe the rationale for the review in the context of what is already known. | 4-5 |
| Objectives | 4 | Provide an explicit statement of the questions being addressed with reference, as applicable, to participants, interventions, comparisons, outcomes and study design (PICOS). Include any hypotheses that relate to particular types of participant-level subgroups. | 4-5 |
| **Methods** | | | |
| Protocol and registration | 5 | Indicate if a protocol exists and where it can be accessed. If available, provide registration information including registration number and registry name. Provide publication details, if applicable. | 6-9 |
| Eligibility criteria | 6 | Specify inclusion and exclusion criteria including those relating to participants, interventions, comparisons, outcomes, study design and characteristics (e.g. years when conducted, required minimum follow-up). Note whether these were applied at the study or individual level i.e. whether eligible participants were included (and ineligible participants excluded) from a study that included a wider population than specified by the review inclusion criteria. The rationale for criteria should be stated. | 6-8 |
| Identifying studies – information sources | 7 | Describe all methods of identifying published and unpublished studies including, as applicable: which bibliographic databases were searched with dates of coverage; details of any hand searching including of conference proceedings; use of study registers and agency or company databases; contact with the original research team and experts in the field; open adverts and surveys. Give the date of last search or elicitation. | 6-7 |
| Identifying studies – search | 8 | Present the full electronic search strategy for at least one database, including any limits used, such that it could be repeated. | Appendix |
| Study selection processes | 9 | State the process for determining which studies were eligible for inclusion. | 6 |
| Data collection processes | 10 | Describe how IPD were requested, collected and managed, including any processes for querying and confirming data with investigators. If IPD were not sought from any eligible study, the reason for this should be stated (for each such study). | 6-7 |
|  |  | If applicable, describe how any studies for which IPD were not available were dealt with. This should include whether, how and what aggregate data were sought or extracted from study reports and publications (such as extracting data independently in duplicate) and any processes for obtaining and confirming these data with investigators. |  |
| Data items | 11 | Describe how the information and variables to be collected were chosen. List and define all study level and participant level data that were sought, including baseline and follow-up information. If applicable, describe methods of standardising or translating variables within the IPD datasets to ensure common scales or measurements across studies. | 6-8 |
| IPD integrity | A1 | Describe what aspects of IPD were subject to data checking (such as sequence generation, data consistency and completeness, baseline imbalance) and how this was done. | 6-7 |
| Risk of bias assessment in individual studies. | 12 | Describe methods used to assess risk of bias in the individual studies and whether this was applied separately for each outcome. If applicable, describe how findings of IPD checking were used to inform the assessment. Report if and how risk of bias assessment was used in any data synthesis. | 9, Appendix |
| Specification of outcomes and effect measures | 13 | State all treatment comparisons of interests. State all outcomes addressed and define them in detail. State whether they were pre-specified for the review and, if applicable, whether they were primary/main or secondary/additional outcomes. Give the principal measures of effect (such as risk ratio, hazard ratio, difference in means) used for each outcome. | 8-9 |
| Synthesis methods | 14 | Describe the meta-analysis methods used to synthesise IPD. Specify any statistical methods and models used. Issues should include (but are not restricted to):   - Use of a one-stage or two-stage approach. - How effect estimates were generated separately within each study and combined across studies (where applicable). - Specification of one-stage models (where applicable) including how clustering of patients within studies was accounted for. - Use of fixed or random effects models and any other model assumptions, such as proportional hazards. - How (summary) survival curves were generated (where applicable). - Methods for quantifying statistical heterogeneity (such as I^2^ and t^2^). - How studies providing IPD and not providing IPD were analysed together (where applicable). - How missing data within the IPD were dealt with (where applicable). | 7-9 |
| Exploration of variation in effects | A2 | If applicable, describe any methods used to explore variation in effects by study or participant level characteristics (such as estimation of interactions between effect and covariates). State all participant-level characteristics that were analysed as potential effect modifiers, and whether these were pre-specified. | 9 |
| Risk of bias across studies | 15 | Specify any assessment of risk of bias relating to the accumulated body of evidence, including any pertaining to not obtaining IPD for particular studies, outcomes or other variables. | 9 |
| Additional analyses | 16 | Describe methods of any additional analyses, including sensitivity analyses. State which of these were pre-specified. | 9 |
| **Results** | | | |
| Study selection and IPD obtained | 17 | Give numbers of studies screened, assessed for eligibility, and included in the systematic review with reasons for exclusions at each stage. Indicate the number of studies and participants for which IPD were sought and for which IPD were obtained. For those studies where IPD were not available, give the numbers of studies and participants for which aggregate data were available. Report reasons for non-availability of IPD. Include a flow diagram. | 10 |
| Study characteristics | 18 | For each study, present information on key study and participant characteristics (such as description of interventions, numbers of participants, demographic data, unavailability of outcomes, funding source, and if applicable duration of follow-up). Provide (main) citations for each study. Where applicable, also report similar study characteristics for any studies not providing IPD. | Appendix |
| IPD integrity | A3 | Report any important issues identified in checking IPD or state that there were none. | 6 |
| Risk of bias within studies | 19 | Present data on risk of bias assessments. If applicable, describe whether data checking led to the up-weighting or down-weighting of these assessments. Consider how any potential bias impacts on the robustness of meta-analysis conclusions. | 10-11, Appendix |
| Results of individual studies | 20 | For each comparison and for each main outcome (benefit or harm), for each individual study report the number of eligible participants for which data were obtained and show simple summary data for each intervention group (including, where applicable, the number of events), effect estimates and confidence intervals. These may be tabulated or included on a forest plot. | Appendix |
| Results of syntheses | 21 | Present summary effects for each meta-analysis undertaken, including confidence intervals and measures of statistical heterogeneity. State whether the analysis was pre-specified, and report the numbers of studies and participants and, where applicable, the number of events on which it is based. | 10-13  Appendix  10-13 |
|  |  | When exploring variation in effects due to patient or study characteristics, present summary interaction estimates for each characteristic examined, including confidence intervals and measures of statistical heterogeneity. State whether the analysis was pre-specified. State whether any interaction is consistent across trials. |  |
|  |  | Provide a description of the direction and size of effect in terms meaningful to those who would put findings into practice. |  |
| Risk of bias across studies | 22 | Present results of any assessment of risk of bias relating to the accumulated body of evidence, including any pertaining to the availability and representativeness of available studies, outcomes or other variables. | Appendix |
| Additional analyses | 23 | Give results of any additional analyses (e.g. sensitivity analyses). If applicable, this should also include any analyses that incorporate aggregate data for studies that do not have IPD. If applicable, summarise the main meta-analysis results following the inclusion or exclusion of studies for which IPD were not available. | 10-13, Appendix |
| **Discussion** | | | |
| Summary of evidence | 24 | Summarise the main findings, including the strength of evidence for each main outcome. | 13-15 |
| Strengths and limitations | 25 | Discuss any important strengths and limitations of the evidence including the benefits of access to IPD and any limitations arising from IPD that were not available. | 15 |
| Conclusions | 26 | Provide a general interpretation of the findings in the context of other evidence. | 13-16 |
| Implications | A4 | Consider relevance to key groups (such as policy makers, service providers and service users). Consider implications for future research. | 16 |
| **Funding** | | | |
| Funding | 27 | Describe sources of funding and other support (such as supply of IPD), and the role in the systematic review of those providing such support. | 16-17 |

© Reproduced with permission of the PRISMA IPD Group, which encourages sharing and reuse for non-commercial purposes

Box A: Search strategy

| **Search strategy**  All prospective *P. vivax* antimalarial clinical trials with a minimum of 28 days follow up, published between Jan 1, 2000 and Feb 16, 2021 were identified by the application of the key terms (listed below) through Medline (Pubmed), Web of Science, Embase and the Cochrane Database of Systematic Reviews. Abstracts of all references containing any mention of antimalarial drugs were manually checked to confirm prospective clinical trials, with review of full text when needed. Studies on prevention, prophylaxis, reviews, animal studies, pregnant women, patients with severe malaria, where chloroquine or artemisinin combination therapy was not used, where adjunctive drugs were given, where schizonticidal treatment was unsupervised, where data were extracted retrospectively from medical records outside of a planned trial, or where data had not been mapped to the WorldWide Antimalarial Resistance Network (WWARN) database by 16^th^ February 2021 were excluded. The year of the study was taken as the year in which the paper was published, although the start and end date of patient enrolment were also recorded. The review process was undertaken by two independent investigators who also performed data extraction (RJC and RNP), and is documented in more detail in Commons et al, Int J Parasitol Drug Drug Res 2017 (1).  **Key terms:**  Literature search (conducted February 2021) with the following key terms (version undertaken in Pubmed): (malaria OR plasmod*) AND (amodiaquine OR atovaquone OR artemisinin OR arteether OR artesunate OR artemether OR artemotil OR azithromycin OR artekin OR chloroquine OR chlorproguanil OR cycloguanil OR clindamycin OR coartem OR dapsone OR dihydroartemisinin OR duo-cotecxin OR doxycycline OR halofantrine OR lumefantrine OR lariam OR malarone OR mefloquine OR naphthoquine OR naphthoquinone OR piperaquine OR primaquine OR proguanil OR pyrimethamine OR pyronaridine OR quinidine OR quinine OR riamet OR sulphadoxine OR tetracycline OR tafenoquine). |
| --- |

Table A: Schizonticidal treatments administered A) classified by elimination half-life and B) as treatment arms categorised by elimination half-life

**A:**

| **Rapid (<1 day)** | **Intermediate (1-7 days)** | **Slow (>7 days)** |
| --- | --- | --- |
| Artemisinins | Dapsone | Amodiaquine |
| Doxycycline | Lumefantrine | Chloroquine |
| Proguanil | Pyrimethamine | Mefloquine |
|  |  | Naphthoquine |
|  |  | Piperaquine |
|  |  | Pyronaridine |
|  |  | Sulfadoxine |
| Combination therapies were categorised based on the drug with the longest elimination half-life | | |

**B:**

| **Treatment arm** | **Number of patients** |
| --- | --- |
| **Rapid elimination half-life regimens** |  |
| Artemisinin | 113 |
| Artemisinin + artesunate | 1 |
| Artesunate | 253 |
| Doxycycline | 15 |
| **Intermediate elimination half-life regimens** |  |
| Artemether-lumefantrine | 548 |
| Artemether-lumefantrine + doxycycline | 1 |
| Artesunate + artemether-lumefantrine | 10 |
| **Slow elimination half-life regimens** |  |
| Amodiaquine | 299 |
| Artemether-lumefantrine + chloroquine | 5 |
| Artemisinin + naphthoquine | 24 |
| Artesunate + amodiaquine | 189 |
| Artesunate + chloroquine | 1 |
| Artesunate + pyronaridine | 224 |
| Artesunate + sulfadoxine-pyrimethamine | 44 |
| Artesunate-mefloquine | 56 |
| Chloroquine | 7718 |
| Chloroquine + doxycycline | 22 |
| Chloroquine + sulfadoxine-pyrimethamine | 57 |
| Chlorproguanil + dapsone | 302 |
| Dihydroartemisinin-piperaquine | 3062 |
| Sulfadoxine-pyrimethamine | 306 |
| **Unknown** | 13 |

Table B: Sensitivity analysis comparing enrolment parasitaemia centiles for febrile patients

| **Variable** | **All studies** | **Studies in which parasitaemia was calculated by the same method^a^** |
| --- | --- | --- |
| Number of studies | 27 | 12 |
| Number of patients | 8,378 | 3,009 |
| 1^st^ centile of parasitaemia (parasites /µL) | 26 | 58 |
| 5^th^ centile of parasitaemia (parasites /µL) | 120 | 176 |
| 10^th^ centile of parasitaemia (parasites /µL) | 278 | 344 |
| 25^th^ centile of parasitaemia (parasites /µL) | 968 | 1,160 |
| Median parasitaemia (parasites /µL) | 3,280 | 3,040 |

^a^Number of parasites per number of white blood cells (WBCs) and assuming a WBC count of 5,500-8,000/µL

Table C: Sensitivity analyses for enrolment parasitaemia centiles and estimated pyrogenic density at the first recurrence

| **Variable** | **Range of parasitaemia (parasites /µL)** | **Coefficient of variation (%)^a^** |
| --- | --- | --- |
| ***Febrile patients at enrolment ^b^*** |  |  |
| 25^th^ centile | 903 – 1048 | 3.05 |
| 10^th^ centile | 260 – 312 | 3.57 |
| 5^th^ centile | 107 – 140 | 5.04 |
| 1^st^ centile | 25 – 32 | 5.67 |
| ***All patients at first recurrence ^c^*** |  |  |
| Pyrogenic density | 900 – 1777 | 23.66 |

Sensitivity analyses were generated by removing each study site one at a time

^a^The coefficient of variation was calculated as the standard deviation divided by the mean of the estimates

^b^There were a total of 49 study sites

^c^There were a total of 68 study sites

Table D: Comparison of baseline characteristics between included and targeted studies

| **Characteristic** | **Included studies** | **Targeted studies** |
| --- | --- | --- |
|  | **(n=50)** | **(n=137)** |
| Region^b^ |  |  |
| Asia-Pacific, studies (%) | 38.8 (77.6%) | 87.8 (64.1%) |
| Africa, studies (%) | 5.2 (10.4%) | 10.2 (7.4%) |
| The Americas, studies (%) | 6.0 (12.0%) | 39.0 (28.5%) |
|  |  |  |
| Year of enrolment^c^ |  |  |
| Pre-2009, studies (%) | 18 (36.0%) | 68 (49.6%) |
| 2009-2021, studies (%) | 32 (64.0%) | 55 (40.1%)^d^ |
|  |  |  |
| Age, years, mean (SD)^a^ | 21.2 (14.5)^e^ | 24.7 (7.4)^f^ |
|  |  |  |
| Female, % of patients, mean (SD)^a^ | 36.8%^g^ | 29.2% (15.9)^h^ |

SD – standard deviation.

^a^ Age, and female percentage of targeted studies calculated using frequency weighted mean or median according to number of patients.

^b^ Multinational studies considered as a proportion of the number of study sites.

^c^ Year of enrolment defined as the year study enrolment completed.

^d^ Year of enrolment not stated for 14 studies.

^e^ Mean or median age not available for 31 patients.

^f^ Mean or median age not available for 54 studies.

^g^ Sex data not available for 28 patients.

^h^ Female percentage not available for 24 studies.

Table E: Reasons for study exclusion

| **Reason** | **Number of Studies** | **Reference** |
| --- | --- | --- |
| Studies of severe malaria | 1 | (2) |
| Studies of pregnant women | 2 | (3,4) |
| Studies on prevention/prophylaxis | 1 | (5) |
| Studies of *P. falciparum* only | 1 | (6) |
| Studies of *P. knowlesi* only | 1 | (7) |
| Studies without chloroquine or artemisinin combination therapy | 14 | (8–21) |
| Studies in which adjunctive drugs were used | 3 | (22–24) |
| Studies not mapped to the WWARN database by 16/02/21 | 124 | (25–148) |
| Studies where not all essential variables were available | 12 | (149–160) |
| Studies with no response from investigators | 1 | (161) |

Table F: Studies excluded from the analysis

| **Author-year** | **Region** | **Country** | **Relapse periodicity^c^** | **Total sample size** | **Female (%)** | **Age (years)** | | **Duration of study (days)** | **Recruitment period** | **Reason excluded** |
| --- | --- | --- | --- | --- | --- | --- | --- | --- | --- | --- |
|  |  |  |  |  |  | **Mean (SD^d^)** | **Median (range)** |  |  |  |
| Abdon-2001(25) | Americas | Brazil | Low | 120 | 37.5 | 27.3 (-) |  | 180 | 1994-1995 | Not mapped to the WWARN database by 16/02/2021 |
| Adak-2001(26) | Asia-Pacific | India | Low | 663 | Not stated | Not stated |  | 365 | Not stated | Not mapped to the WWARN database by 16/02/2021 |
| Ahmedou-2015(27) | Africa | Mauritania | Low | 128 | 53.1 | 27.2 (-) |  | 28 | 2013 | Not mapped to the WWARN database by 16/02/2021 |
| Alecrim-2000(8) | Americas | Brazil | Low | 3 | Not stated | Not stated |  | 28 | 1996-1997 | Studies without CQ or ACT therapy |
| Alvarez-2006(161) | Americas | Colombia | Low | 210 | 33 | 30.1 (12.8) |  | 180 | 2001 | No response from investigators |
| Amaratunga-2014(28) | Asia-Pacific | Cambodia | High | 87 | 20.7 | Not stated | 26 (4-68) | 28 | 2012-2013 | Not mapped to the WWARN database by 16/02/2021 |
| Anez-2012(29) | Americas | Bolivia | Low | 223 | 42.2 | Not stated |  | 28 | 2006-2007 | Not mapped to the WWARN database by 16/02/2021 |
| Anez-2015(149) | Americas | Bolivia | Low | 100 | 36 | Not stated | 20 (5-69) | 28 | 2011 | Not all essential variables available |
| Asih-2011(30) | Asia-Pacific | Indonesia | High | 73 | 50.7 | Not stated | 13.2 (2-60) | 28 | 2007 | Not mapped to the WWARN database by 16/02/2021 |
| Assefa-2015(31) | Africa | Ethiopia | Low | 63 | 41.7 | Not stated | 23 (4-59) | 28 | 2014 | Not mapped to the WWARN database by 16/02/2021 |
| Awab-2017(32) | Asia-Pacific | Afghanistan | Low | 593 | 34.1 | Not stated | 15.5 (-) | 365 | 2009-2013 | Not mapped to the WWARN database by 16/02/2021 |
| Azarian Moghadam-2018(33) | Asia-Pacific | Iran | Low | 170 | Not stated | Not stated |  | 28 | 2013-2014 | Not mapped to the WWARN database by 16/02/2021 |
| Baird-2002(34) | Americas | Guyana | Low | 13 | 21.9 | 31 (-) |  | 28 | 1998 | Not mapped to the WWARN database by 16/02/2021 |
| Barnadas-2008a(35) | Africa | Madagascar | Low | 105 | 53 | 11.2 (-) |  | 28 | 2006 | Not mapped to the WWARN database by 16/02/2021 |
| Barnadas-2008b(9) | Africa | Madagascar | Low | 14 | 43.4 | Not stated | 7.5 (-) | 28 | 2006-2007 | Studies without CQ or ACT therapy |
| Benjamin-2012(36) | Asia-Pacific | PNG^a^ | High | 2 | 35.4 | Not stated |  | 56 | Not stated | Not mapped to the WWARN database by 16/02/2021 |
| Bergonzoli-2000(37) | Americas | Costa Rica | Low | 132 | Not stated | 30.5 (-) |  | 180 | 1994 | Not mapped to the WWARN database by 16/02/2021 |
| Betuela-2012(38) | Asia-Pacific | PNG^a^ | High | 433 | 49.4 | 3.2 (-) |  | 280 | 2008 | Not mapped to the WWARN database by 16/02/2021 |
| Beyene-2016(39) | Africa | Ethiopia | Low | 76 | 32 | Not stated | 19 (3-54) | 28 | 2014 | Not mapped to the WWARN database by 16/02/2021 |
| Blair-Trujillo-2002(40) | Americas | Colombia | Low | 33 | Not stated | 20 (12) |  | 28 | 1998 | Not mapped to the WWARN database by 16/02/2021 |
| Brasil-2018(41) | Americas | Brazil | Low | 213 | 43.7 | Not stated |  | 180 | Not stated | Not mapped to the WWARN database by 16/02/2021 |
| Buchachart-2001(42) | Asia-Pacific | Thailand | High | 593 | 37.1 | 25 (-) |  | 28 | 1992-1997 | Not mapped to the WWARN database by 16/02/2021 |
| Carmona-Fonseca-2006(43) | Americas | Colombia | Low | 228 | 33 | 30.1 (12.8) |  | 28 | 2003-2004 | Not mapped to the WWARN database by 16/02/2021 |
| Carmona-Fonseca-2008(150) | Americas | Colombia | Low | 82 | 38 | Not stated | - (4-10) | 30 | 2000-2003 | Not all essential variables available |
| Carmona-Fonseca-2009(44) | Americas | Colombia | Low | 188 | 30.4 | Not stated |  | 120 | 2001-2003 | Not mapped to the WWARN database by 16/02/2021 |
| Carmona-Fonseca-2010(45) | Americas | Colombia | Low | 79 | Not stated | Not stated |  | 120 | 2005-2008 | Not mapped to the WWARN database by 16/02/2021 |
| Castillo-2002(46) | Americas | Colombia | Low | 50 | 84.1 | 28 (-) |  | 28 | 1998-1999 | Not mapped to the WWARN database by 16/02/2021 |
| Cheoymang-2015(47) | Asia-Pacific | Thailand | High | 85 | 34.1 | Not stated |  | 42 | 2008-2009 | Not mapped to the WWARN database by 16/02/2021 |
| Congpuong-2002(48) | Asia-Pacific | Thailand | High | 26 | 26.9 | 39 (-) |  | 28 | 2000 | Not mapped to the WWARN database by 16/02/2021 |
| Congpuong-2010(6) | Asia-Pacific | Thailand | High | 240 | 18.3 | Not stated | 27 (4-69) | 42 | 2008-2009 | Pf only |
| Congpuong-2011(49) | Asia-Pacific | Thailand | High | 212 | 27.8 | Not stated | 25 (2-80) | 28 | 2009-2010 | Not mapped to the WWARN database by 16/02/2021 |
| da Silva-2003(50) | Americas | Brazil | Low | 240 | 23.3 | 32.9 (-) |  | 180 | Not stated | Not mapped to the WWARN database by 16/02/2021 |
| Daher-2018(151) | Americas | Brazil | Low | 264 | 29.5 | Not stated | - (18-65) | 63 | 2012-2015 | Not all essential variables available |
| Daneshvar-2010(51) | Asia-Pacific | Malaysia | High | 23 | 0 | 38.5 (7.6) |  | 28 | -2007 | Not mapped to the WWARN database by 16/02/2021 |
| de Santana Filho-2007(52) | Americas | Brazil | Low | 166 | Not stated | Not stated |  | 28 | 2004-2005 | Not mapped to the WWARN database by 16/02/2021 |
| de Sena-2019(152) | Americas | Brazil | Low | 81 | 0 | Not stated | 9 (2-14) | 42 | 2016-2017 | Not all essential variables available |
| Das-2020(2) | Asia-Pacific | India | Low | 32 | Not stated | Not stated |  | 28 | 2013-2015 | Severe malaria |
| Delgado-Ratto-2014(53) | Americas | Peru | Low | 37 | 48.6 | Not stated | 15 (-) | 720 | 2008 | Not mapped to the WWARN database by 16/02/2021 |
| Dharmawardena-2017(54) | Asia-Pacific | Sri Lanka | Low | 21 | 6.8 | 35.5 (-) |  | 365 | 2015-2016 | Not mapped to the WWARN database by 16/02/2021 |
| Dilmec-2010(55) | Asia-Pacific | Turkey | Low | 42 | 47.2 | Not stated |  | 28 | Not stated | Not mapped to the WWARN database by 16/02/2021 |
| Dua-2001(56) | Asia-Pacific | India | Low | 5541 | Not stated | Not stated |  | 540 | 1987-2000 | Not mapped to the WWARN database by 16/02/2021 |
| Duarte-2001(57) | Americas | Brazil | Low | 50 | 24 | 31.8 (12.8) |  | 180 | 1997-1998 | Not mapped to the WWARN database by 16/02/2021 |
| Dunne-2005(58) | Asia-Pacific | India | Low | 200 | 6 | 31.8 ( - ) |  | 28 | 1998-2001 | Not mapped to the WWARN database by 16/02/2021 |
| Eibach-2012(59) | Americas | Guyana | Low | 74 | 9.5 | Not stated | 24 (5-57) | 28 | 2009-2010 | Not mapped to the WWARN database by 16/02/2021 |
| Fernandopulle-2003(10) | Asia-Pacific | Sri Lanka | Low | 6 | Not stated | Not stated |  | 180 | Not stated | Studies without CQ or ACT therapy |
| Fryauff-2002(60) | Asia-Pacific | Indonesia | High | 36 | 38.9 | 14 (-) |  | 28 | 1998 | Not mapped to the WWARN database by 16/02/2021 |
| Fukuda-2017(61) | Asia-Pacific | Thailand | High | 70 | 17 | Not stated | 30 (-) | 120 | 2003-2005 | Not mapped to the WWARN database by 16/02/2021 |
| Ganguly-2013(62) | Asia-Pacific | India | High | 250 | 10.8 | 25.2 (-) |  | 42 | 2011-2012 | Not mapped to the WWARN database by 16/02/2021 |
| Genton-2005(63) | Asia-Pacific | PNG^a^ | High | 18 | 52 | 5.1 ( - ) |  | 28 | 1994-1995 | Not mapped to the WWARN database by 16/02/2021 |
| Gomes-2015(153) | Americas | Brazil | Low | 103 | 33 | 30 (12) | - (16-60) | 28 | 2011 | Not all essential variables available |
| Graf-2012(64) | Americas | Peru | Low | 540 | Not stated | Not stated |  | 210 | 2005-2008 | Not mapped to the WWARN database by 16/02/2021 |
| Grigg-2018(7) | Asia-Pacific | Malaysia | High | 116 | Not stated | Not stated | 15 (9-30) | 42 | 2013-2015 | Pk only |
| Hamedi-2002(65) | Asia-Pacific | Iran | Low | 40 | 12.5 | 27.2 (9.1) |  | 28 | 1999-2001 | Not mapped to the WWARN database by 16/02/2021 |
| Hamedi-2004(11) | Asia-Pacific | Thailand | High | 42 | 31 | 24 (8.8) |  | 28 | 2001-2002 | Studies without CQ or ACT therapy |
| Hamid-2018(12) | Africa | Sudan | Low | 78 | 48.7 | Not stated | 17 (10-27) | 42 | 2015-2016 | Studies without CQ or ACT therapy |
| Han-2020(66) | Asia-Pacific | Myanmar | High | 206 | 35 | 27.2 (-) |  | 28 | 2017-2019 | Not mapped to the WWARN database by 16/02/2021 |
| Hapuarachchi-2004(67) | Asia-Pacific | Sri Lanka | Low | 42 | 0 | Not stated |  | 28 | 2002 | Not mapped to the WWARN database by 16/02/2021 |
| Htun-2017(68) | Asia-Pacific | Myanmar | High | 85 | 28.2 | Not stated | 24 (-) | 28 | 2012-2014 | Not mapped to the WWARN database by 16/02/2021 |
| Ketema-2011(69) | Africa | Ethiopia | Low | 87 | 57.4 | Not stated | 8 (0.75-52) | 28 | 2009 | Not mapped to the WWARN database by 16/02/2021 |
| Khan-2006(13) | Asia-Pacific | Pakistan | Low | 36 | 39.2 | 15.7 (9.9) | 12.6 (-) | 28 | 2003 | Studies without CQ or ACT therapy |
| Kheng-2015(14) | Asia-Pacific | Cambodia | High | 75 | 16 | Not stated | 24 (-) | 56 | 2013-2014 | Studies without CQ or ACT therapy |
| Kim-2011(5) | Asia-Pacific | ROK^b^ | Low | 85 | 0 | 22.5 (3.3) |  | 28 | 2006 | Studies on prevention or prophylaxis |
| Kinzer-2010(70) | Asia-Pacific | Vanuatu | High | 21 | 66.7 | Not stated | 11 (5 -53) | 28 | 2005 | Not mapped to the WWARN database by 16/02/2021 |
| Kolaczinski-2007(71) | Asia-Pacific | Afghanistan | Low | 190 | 43 | Not stated | 8.5 (-) | 42 | 2004 | Not mapped to the WWARN database by 16/02/2021 |
| Krudsood-2006(72) | Asia-Pacific | Thailand | High | 141 | 74.6 | 25.0 (6.7) |  | 28 | 2004-2005 | Not mapped to the WWARN database by 16/02/2021 |
| Krudsood-2007(73) | Asia-Pacific | Thailand | High | 98 | 31.4 | 24.3 (6.3) |  | 28 | 2004-2005 | Not mapped to the WWARN database by 16/02/2021 |
| Krudsood-2008(15) | Asia-Pacific | Thailand | High | 399 | 35.1 | 24 (-) |  | 28 | Not stated | Studies without CQ or ACT therapy |
| Kumar-2016(74) | Asia-Pacific | India | Low | 114 | 7.9 | 38.9 (12.4) |  | 480 | 2012-2015 | Not mapped to the WWARN database by 16/02/2021 |
| Kurcer-2004(75) | Asia-Pacific | Turkey | Low | 112 | 51.4 | Not stated |  | 28 | 2002 | Not mapped to the WWARN database by 16/02/2021 |
| Kurcer-2006(76) | Asia-Pacific | Turkey | Low | 91 | 34.1 | Not stated |  | 28 | 2004 | Not mapped to the WWARN database by 16/02/2021 |
| Lacerda-2019(77) | Multicentred | Multinational | Both | 522 | 24.9 | Not stated |  | 180 | 2013-2017 | Not mapped to the WWARN database by 16/02/2021 |
| Lacy-2002(16) | Asia-Pacific | Indonesia | High | 16 | 18.75 | 32.8 (-) |  | 28 | 1999-2000 | Studies without CQ or ACT therapy |
| Leang-2013(78) | Asia-Pacific | Cambodia | High | 390 | 29 | 18.8 (-) |  | 28 | 2008-2011 | Not mapped to the WWARN database by 16/02/2021 |
| Leang-2019(79) | Asia-Pacific | Cambodia | High | 120 | 5 | Not stated |  | 28 | 2018 | Not mapped to the WWARN database by 16/02/2021 |
| Lee-2009b(80) | Asia-Pacific | ROK^b^ | Low | 142 | 0 | Not stated | 21 (19 -50) | 28 | 2007 | Not mapped to the WWARN database by 16/02/2021 |
| Leslie-2004(154) | Asia | Pakistan | Low | 595 | Not stated | Not stated |  | 270 | 2000 | Not all essential variables available |
| Leslie-2008(155) | Asia | Pakistan | Low | 200 | Not stated | Not stated |  | 335 | 2004-2006 | Not all essential variables available |
| Liang-2009(81) | Asia-Pacific | Myanmar | High | 48 | Not stated | Not stated |  | 28 | Not stated | Not mapped to the WWARN database by 16/02/2021 |
| Liu-2013(82) | Asia-Pacific | China | High | 260 | 14 | Not stated |  | 365 | 2009-2010 | Not mapped to the WWARN database by 16/02/2021 |
| Liu-2014(83) | Asia-Pacific | China | High | 750 | 41.5 | 25.2 (6.8) |  | 28 | 2008-2013 | Not mapped to the WWARN database by 16/02/2021 |
| Llanos-Cuentas-2013(84) | Americas | Brazil | Low | 329 | 28.8 | 34.8 (-) |  | 180 | 2011-2013 | Not mapped to the WWARN database by 16/02/2021 |
| Llanos-Cuentas-2018(17) | Americas | Peru | Low | 45 | 42.9 | Not stated |  | 28 | 2015 | Studies without CQ or ACT therapy |
| Llanos-Cuentas-2019(85) | Multicentred | Multinational | Both | 251 | 33.5 | Not stated |  | 180 | 2014-2017 | Not mapped to the WWARN database by 16/02/2021 |
| Lo-2016(86) | Asia-Pacific | Myanmar | High | 130 | Not stated | Not stated |  | 90 | 2011-2013 | Not mapped to the WWARN database by 16/02/2021 |
| Lon-2014(87) | Asia-Pacific | Cambodia | High | 91 | 3.8 | 33.8 (-) |  | 180 | 2010-2011 | Not mapped to the WWARN database by 16/02/2021 |
| MacDonald-Ottevanger-2017(88) | Americas | Suriname | Low | 85 | 33.3 | Not stated |  | 365 | 2006-2008 | Not mapped to the WWARN database by 16/02/2021 |
| Macareo-2013(89) | Asia-Pacific | Thailand | High | 20 | Not stated | Not stated |  | 90 | Not stated | Not mapped to the WWARN database by 16/02/2021 |
| Machado-2003(90) | Americas | Brazil | Low | 30 | Not stated | Not stated |  | 28 | Not stated | Not mapped to the WWARN database by 16/02/2021 |
| Maguire-2002(91) | Asia-Pacific | Indonesia | High | 73 | 62.5 | 32 (-) |  | 28 | Not stated | Not mapped to the WWARN database by 16/02/2021 |
| Maguire-2006(92) | Asia-Pacific | Indonesia | High | 514 | 35.2 | 22.6 ( - ) |  | 28 | 1996-1999 | Not mapped to the WWARN database by 16/02/2021 |
| Manandhar-2013(93) | Asia-Pacific | Nepal | High | 137 | 19 | Not stated |  | 180 | 2010-2011 | Not mapped to the WWARN database by 16/02/2021 |
| Maneeboonyang-2011(94) | Asia-Pacific | Thailand | High | 92 | 40 | Not stated |  | 90 | 2005-2006 | Not mapped to the WWARN database by 16/02/2021 |
| Marfurt-2007(22) | Asia-Pacific | PNG^a^ | High | 104 | Not stated | 3 (-) |  | 28 | 2003-2005 | Adjunctive drugs were used (other than PQ) |
| McGready-2002(3) | Asia-Pacific | Thailand | High | 130 | 100 | 25 (7) |  | 28 | 1995-2000 | Pregnant women only |
| Mesa-Echeverry-2019(95) | Americas | Colombia | Low | 80 | 48.1 | 25.8 (14.4) | 22 (-) | 28 | 2012-2013 | Not mapped to the WWARN database by 16/02/2021 |
| Miahipour-2013(96) | Asia-Pacific | Iran | Low | 180 | 23.3 | Not stated |  | 540 | 2008-2011 | Not mapped to the WWARN database by 16/02/2021 |
| Mishra-2016(97) | Asia-Pacific | India | Low | 401 | 17.5 | Not stated |  | 42 | 2011-2012 | Not mapped to the WWARN database by 16/02/2021 |
| Mohapatra-2002(98) | Asia-Pacific | India | Low | 110 | 36.4 | Not stated |  | 365 | 1998-2000 | Not mapped to the WWARN database by 16/02/2021 |
| Moore-2016(4) | Asia-Pacific | PNG^a^ | High | 1 | 100 | Not stated | 22 (-) | 42 | Not stated | Pregnant women only |
| Muhamad-2011(99) | Asia-Pacific | Thailand | High | 130 | 50.8 | Not stated | 22 (-) | 42 | 2008-2009 | Not mapped to the WWARN database by 16/02/2021 |
| Nandy-2003(100) | Asia-Pacific | India | Both | 800 | Not stated | Not stated |  | 28 | 1998-2001 | Not mapped to the WWARN database by 16/02/2021 |
| Nateghpour-2007(101) | Asia-Pacific | Iran | Low | 225 | 25.6 | Not stated |  | 28 | 2004-2005 | Not mapped to the WWARN database by 16/02/2021 |
| Nateghpour-2009(102) | Asia-Pacific | Iran | Low | Not stated | Not stated | Not stated |  | 28 | Not stated | Not mapped to the WWARN database by 16/02/2021 |
| Negreiros-2016(103) | Americas | Brazil | Low | 119 | 45.4 | Not stated | 23.4 (5-67.3) | 168 | 2014 | Not mapped to the WWARN database by 16/02/2021 |
| Nelwan-2015(156) | Asia | Indonesia | High | 180 | Not stated | 29 | - (21-50) | 365 | 2013 | Not all essential variables available |
| Nyunt-2017(104) | Asia-Pacific | Myanmar | High | 1114 | 38.5 | 24.2 (9) |  | 28 | 2009-2016 | Not mapped to the WWARN database by 16/02/2021 |
| Orjuela-Sanchez-2009(157) | Americas | Brazil | Low | 164 | Not stated | Not stated | - (0-75) | 647 | 2004-2006 | Not all essential variables available |
| Osorio-2007(105) | Americas | Colombia | Low | 22 | 50 | Not stated |  | 28 | 2002-2003 | Not mapped to the WWARN database by 16/02/2021 |
| Pareek-2015(106) | Asia-Pacific | India | Low | 360 | 17.3 | Not stated | 20 (-) | 180 | Not stated | Not mapped to the WWARN database by 16/02/2021 |
| Pedro-2012(107) | Americas | Brazil | Low | 47 | 24.5 | Not stated |  | 28 | 2005-2011 | Not mapped to the WWARN database by 16/02/2021 |
| Perez-2008(108) | Americas | Colombia | Low | 50 | 42.4 | Not stated |  | 28 | 2006 | Not mapped to the WWARN database by 16/02/2021 |
| Pham-2019(109) | Asia-Pacific | Vietnam | High | 260 | 38.8 | Not stated |  | 730 | 2009-2011 | Not mapped to the WWARN database by 16/02/2021 |
| Phong-2019(110) | Asia-Pacific | Vietnam | High | 16 | Not stated | Not stated |  | 28 | 2015-2016 | Not mapped to the WWARN database by 16/02/2021 |
| Phyo-2016(23) | Asia-Pacific | Thailand | High | 40 | 13 | 28.1 (9) |  | 30 | 2003-2013 | Adjunctive drugs were used (other than PQ) |
| Pinto-2003(111) | Americas | Brazil | Low | 132 | 37.9 | 30.7 (-) |  | 28 | 1997-1998 | Not mapped to the WWARN database by 16/02/2021 |
| Popovici-2018a(112) | Asia-Pacific | Cambodia | High | 40 | 32.5 | Not stated |  | 63 | 2014 | Not mapped to the WWARN database by 16/02/2021 |
| Popovici-2018b(113) | Asia-Pacific | Cambodia | High | 50 | 20 | Not stated |  | 63 | 2014 | Not mapped to the WWARN database by 16/02/2021 |
| Pukrittayakamee-2000(114) | Asia-Pacific | Thailand | High | 207 | 0 | 25 (9) |  | 28 | 1992-1998 | Not mapped to the WWARN database by 16/02/2021 |
| Pukrittayakamee-2001(18) | Asia-Pacific | Thailand | High | 27 | 0 | 24 (8) |  | 28 | 1995-1998 | Studies without CQ or ACT therapy |
| Pukrittayakamee-2010(158) | Asia | Thailand | High | 85 | 0 | 24.6 (10.1) | - (14-61) | 28 | 1996-1998 | Not all essential variables available |
| Rajgor-2003(115) | Asia-Pacific | India | Low | 273 | 12.1 | Not stated |  | 180 | 1998-2000 | Not mapped to the WWARN database by 16/02/2021 |
| Rajgor-2014(116) | Asia-Pacific | India | Low | 1556 | 4.8 | 31.2 (-) |  | 180 | Not stated | Not mapped to the WWARN database by 16/02/2021 |
| Rios-2013(117) | Americas | Colombia | Low | 152 | 39.5 | 38 (-) |  | 28 | 2002-2011 | Not mapped to the WWARN database by 16/02/2021 |
| Rogers-2009(118) | Asia-Pacific | Cambodia | High | 110 | 11 | 21.2 (6.7) |  | 42 | 2006-2008 | Not mapped to the WWARN database by 16/02/2021 |
| Ruebush-2003(119) | Americas | Peru | Low | 242 | 40.9 | 19 (-) |  | 28 | 1998-2001 | Not mapped to the WWARN database by 16/02/2021 |
| Saravu-2012(120) | Asia-Pacific | India | Low | 110 | 26.3 | Not stated | 28.5 (-) | 28 | 2007-2009 | Not mapped to the WWARN database by 16/02/2021 |
| Saravu-2018(159) | Asia | India | Low | 50 | 10 | 42 (16) | - (18-76) | 180 | 2017 | Not all essential variables available |
| Seifu-2017(121) | Africa | Ethiopia | Low | 87 | 29.7 | Not stated | 20 (1-65) | 28 | 2013 | Not mapped to the WWARN database by 16/02/2021 |
| Senn-2013(122) | Asia-Pacific | PNG^a^ | High | 688 | Not stated | Not stated |  | 42 | 2006-2010 | Not mapped to the WWARN database by 16/02/2021 |
| Shaikh-2017a(123) | Asia-Pacific | Pakistan | Low | 100 | 45.1 | Not stated |  | 42 | 2012-2013 | Not mapped to the WWARN database by 16/02/2021 |
| Shaikh-2017b(124) | Asia-Pacific | Pakistan | Low | 103 | 39 | Not stated | - (1-15) | 42 | 2016 | Not mapped to the WWARN database by 16/02/2021 |
| Shalini-2014(125) | Asia-Pacific | India | Low | 125 | 5 | 25.9 (10.5) |  | 28 | 2010 | Not mapped to the WWARN database by 16/02/2021 |
| Shumbej-2019(126) | Africa | Ethiopia | Low | 87 | 45.7 | Not stated | 19.0 (1.5-42.0) | 28 | 2016-2017 | Not mapped to the WWARN database by 16/02/2021 |
| Silachamroon-2003(19) | Asia-Pacific | Thailand | High | 801 | 30.3 | 26 (-) |  | 28 | 1999-2001 | Studies without CQ or ACT therapy |
| Singh-2000(127) | Asia-Pacific | India | Low | 75 | 29.3 | 28 (14) |  | 28 | 1998-1999 | Not mapped to the WWARN database by 16/02/2021 |
| Solari Soto-2002(128) | Americas | Peru | Low | 60 | Not stated | Not stated |  | 60 | 1998-1999 | Not mapped to the WWARN database by 16/02/2021 |
| Soto-2001(129) | Americas | Colombia | Low | 28 | 0 | Not stated |  | 28 | Not stated | Not mapped to the WWARN database by 16/02/2021 |
| Srivastava-2008(130) | Asia-Pacific | India | Low | 138 | 47.1 | Not stated |  | 28 | 2003-2005 | Not mapped to the WWARN database by 16/02/2021 |
| Sumawinata-2003(131) | Asia-Pacific | Indonesia | High | 29 | Not stated | Not stated | 24 (5-40) | 28 | 1995 | Not mapped to the WWARN database by 16/02/2021 |
| Sutanto-2013(160) | Asia | Indonesia | High | 116 | 0 | 27 | - (22-42) | 365 | 2010-2011 | Not all essential variables available |
| Takeuchi-2010(132) | Asia-Pacific | Thailand | High | 216 | 39.8 | Not stated |  | 90 | 2007-2009 | Not mapped to the WWARN database by 16/02/2021 |
| Tasanor-2006(133) | Asia-Pacific | Thailand | High | 58 | 41.9 | Not stated | 22 (-) | 28 | 2002-2004 | Not mapped to the WWARN database by 16/02/2021 |
| Tavul-2018(20) | Asia-Pacific | PNG^a^ | High | 70 | Not stated | Not stated |  | 42 | 2012-2014 | Studies without CQ or ACT therapy |
| Taylor-2000(134) | Asia-Pacific | Vietnam | High | 54 | 51.7 | Not stated | 14 (5-40) | 28 | 1995 | Not mapped to the WWARN database by 16/02/2021 |
| Teka-2008(135) | Africa | Ethiopia | Low | 87 | 41.4 | Not stated | 16 (0.7-52) | 28 | 2006 | Not mapped to the WWARN database by 16/02/2021 |
| Tjitra-2002(136) | Asia-Pacific | Indonesia | High | 37 | 33 | 8.8 (-) |  | 28 | 1999 | Not mapped to the WWARN database by 16/02/2021 |
| Tjitra-2012(137) | Asia-Pacific | Indonesia | High | 401 | 15 | 26.2 (9.2) | - (15-69) | 42 | 2007-2008 | Not mapped to the WWARN database by 16/02/2021 |
| Valecha-2016(138) | Asia-Pacific | India | Both | 317 | 8.2 | 33.7 (13.5) |  | 42 | 2011-2013 | Not mapped to the WWARN database by 16/02/2021 |
| Valibayov-2003(139) | Asia-Pacific | Azerbaijan | Low | 153 | 42.5 | 30.8 (-) |  | 28 | Not stated | Not mapped to the WWARN database by 16/02/2021 |
| Van Den Eede-2011(140) | Americas | Peru | Low | 51 | 49 | Not stated | 15 (2-80) | 365 | 2008 | Not mapped to the WWARN database by 16/02/2021 |
| Vijaykadga-2004(141) | Asia-Pacific | Thailand | High | 161 | 19.3 | 32.3 (-) |  | 28 | 2003 | Not mapped to the WWARN database by 16/02/2021 |
| Villalobos-Salcedo-2000(142) | Americas | Brazil | Low | 79 | 21.5 | 31.7 (-) |  | 28 | 1998 | Not mapped to the WWARN database by 16/02/2021 |
| Walsh-2004(143) | Asia-Pacific | Thailand | High | 80 | 68 | Not stated |  | 168 | 1998-1999 | Not mapped to the WWARN database by 16/02/2021 |
| Waqar-2016(144) | Asia-Pacific | Pakistan | Low | 52 | Not stated | 5.4 (3.4) |  | 28 | 2013 | Not mapped to the WWARN database by 16/02/2021 |
| White-2016(24) | Asia-Pacific | Multinational | High | 11 | Not stated | Not stated |  | 28 | 2013 | Adjunctive drugs were used (other than PQ) |
| Yadav-2002(145) | Asia-Pacific | India | Low | 1482 | Not stated | Not stated |  | 365 | 1988-1991 | Not mapped to the WWARN database by 16/02/2021 |
| Yeramian-2005(21) | Asia-Pacific | Thailand | High | 9 | 11.1 | 24.2 (-) |  | 28 | 2003 | Studies without CQ or ACT therapy |
| Yeshiwondim-2010(146) | Africa | Ethiopia | Low | 290 | 45.9 | 23 (12.0) |  | 28 | 2003 | Not mapped to the WWARN database by 16/02/2021 |
| Yohannes-2011(147) | Africa | Ethiopia | Low | 159 | 52.1 | Not stated | 19 (10-26) | 28 | 2004-2005 | Not mapped to the WWARN database by 16/02/2021 |
| Zhu-2013(148) | Asia-Pacific | China | Low | 39 | 42.1 | 43 (-) |  | 28 | 2008-2009 | Not mapped to the WWARN database by 16/02/2021 |

^a^ PNG – Papua New Guinea.

^b^ ROK – Republic of Korea.

^c^ Low relapse periodicity ≤47 days.

^d^ SD – standard deviation.

Table G: Studies included in the analysis

| **WWARN ID** | **Author-year** | **Country** | **Recruitment period** | **Age of participants** | | | **Follow up (days)** | **Parasitaemia threshold on day 0 (Yes/No)** | **Method of parasitaemia calculation** | **Microscopy quality assurance information available (Yes/No)** | **Details on microscopy quality assurance** |
| --- | --- | --- | --- | --- | --- | --- | --- | --- | --- | --- | --- |
|  |  |  |  | **<5 years** | **≥5 - <15 years** | **≥15 years** |  |  |  |  |  |
| TCGQS | Abdallah-2012(162) | Sudan | 2011 | 1 | 11 | 25 | 28 | No | Thick: parasites per 200 WBCs, WBCC assumed 8000/μL | Yes | All slides checked by ≥2 microscopists |
| VUSIG | Abreha-2017(163) | Ethiopia | 2012 - 2014 | 30 | 126 | 241 | 713 | No | Thick films | Yes | All slides checked by ≥2 microscopists |
| MYUES | Awab-2010(164) | Afghanistan | 2007 - 2009 | 73 | 258 | 205 | 56 | No | Thick: parasites per 200 WBCs, WBCC assumed 8000/μL | Yes | 10% of slides cross-checked in reference laboratory |
| QMZCN | Barber-2013(165) | Malaysia | 2010 - 2015 | 0 | 4 | 58 | 161 | No | Thick & thin: thick = parasites per 200 WBCs. Thin film = parasites per 1000 RBCs | No | N/A |
| ABJMD | Chu-2018(166) | Thailand | 2010 - 2011 | 54 | 157 | 433 | 412 | No | Thick & thin: thick = parasites per 500 WBCs, WBCC assumed 8000/μL. Thin film per 1000 RBCs if "higher parasitaemias" | Yes | All slides checked by ≥2 microscopists |
| UJIUX | Chu-2019(167) | Thailand | 2012 - 2014 | 25 | 181 | 448 | 408 | No | Thick & thin: thick = parasites per 500 WBCs. Thin film = parasites per 1000 RBCs | No | N/A |
| HZLBF | Dao-2007(168) | Vietnam | 2003 - 2005 | 0 | 0 | 28 | 28 | Yes | Thick: parasites per 200 WBCs | Yes | All slides checked by ≥2 microscopists |
| DAQJD | Getachew-2015(169) | Ethiopia | 2010 - 2013 | 76 | 117 | 92 | 29 | Yes | Thick & thin: parasites per 200 WBCs, WBCC assumed 8000/μL | Yes | All slides checked by ≥2 microscopists |
| XRBVP | Gonzalez-Ceron-2015(170) | Mexico | 2008 - 2010 | 9 | 36 | 114 | 365 | Yes | Thick: parasites per 200 WBCs if ≥10 parasites per 200 WBCs, parasites per 500 WBCs if <10 parasites per 200 WBCs, WBCC assumed 7000/μL | Yes | All slides checked by ≥2 microscopists |
| CAKNG | Grigg-2016(171) | Malaysia | 2013 - 2014 | 3 | 41 | 59 | 406 | No | Thick & thin: thick = parasites per 200 or 500 WBCs, WBCC of each patient used. Thin film per 1000 RBCs | No | N/A |
| EUUFC | Guthmann-2008(172) | Myanmar | 2002 - 2003 | 83 | 95 | 74 | 28 | Yes | Thick: parasites per 200 WBCs if ≥10 parasites per 200 WBCs, parasites per 500 WBCs if <10 parasites per 200 WBCs, WBCC assumed 8000/μL | Yes | Slides read twice, 55% cross-checked at reference laboratory |
| QCKHC | Hasugian-2007(173) | Indonesia | 2005 | 36 | 23 | 47 | 55 | No | Thick & thin: thick = parasites per 200 WBCs, WBCC of each patient used. Thin film if >200 parasites per 200 WBCs | Yes | All slides checked by ≥2 microscopists |
| NEWPD | Hasugian-2009(174) | Indonesia | 2004 - 2005 | 15 | 31 | 32 | 51 | No | Thick & thin: thick = parasites per 200 WBCs, WBCC assumed 7300/μL. Thin film if >200 parasites per 200 WBCs | Yes | All slides checked by ≥2 microscopists |
| QQOYU | Heidari-2012(175) | Iran | 2010 | 15 | 45 | 210 | 28 | No | Thick & thin films | No | N/A |
| ZMNBX | Hien-unpublished* | Vietnam | 2002 - 2003 | 0 | 65 | 667 | 238 | Yes | Thick & thin films | No | N/A |
| RAKYS | Hwang-2013(176) | Ethiopia | 2009 | 32 | 83 | 127 | 42 | Yes | Thick & thin: parasites per 200 WBCs, WBCC assumed 8000/μL | Yes | All slides checked by ≥2 microscopists |
| OSJAH | Karunajeewa-2008(177) | Papua New Guinea | 2005 | 171 | 2 | 0 | 52 | Yes | Thick & thin: thick = parasites on minimum of 40 high power fields x calculated volume of blood, thin = parasites per 2000 RBCs, RBCC assumed 5 × 10^6 RBCs/μL | Yes | All slides checked by ≥2 microscopists |
| USDTS | Karunajeewa-unpublished* | Vanuatu | 2013 | 11 | 10 | 5 | 86 | No | Thick & thin: WBCC assumed 8000/μL | No | N/A |
| GZQSB | Ketema-2009(178) | Ethiopia | 2007 | 26 | 36 | 22 | 28 | Yes | Unclear if thick or thin: parasites per 1000 WBCs, WBCC assumed 8000/μL | Yes | All slides checked by ≥2 microscopists |
| LYHCD | Ladeia-Andrade-2019(179) | Brazil | 2014 - 2015 | 0 | 50 | 138 | 545 | No | Thick & thin: parasites per 200 WBCs if ≥10 parasites per 200 WBCs, parasites per 500 WBCs if <10 parasites per 200 WBCs, WBCC assumed 8000/μL | Yes | All negative slides checked by ≥2 microscopists. Parasite densities estimated by an expert microscopist |
| YTHTA | Laman-2014(180) | Papua New Guinea | 2011 - 2012 | 40 | 1 | 0 | 42 | Yes | Thick: parasites per 200 WBCs, WBCC assumed 6000/μL | Yes | All slides checked by ≥2 microscopists |
| UXLEY | Leslie-2007(181) | Multinational | 2004 - 2006 | 27 | 552 | 188 | 28 | No | Unclear if thick or thin: parasites per 200-500 WBCs, WBCC assumed 8000/μL | Yes | All slides checked by ≥2 microscopists |
| WANHR | Ley-2016(182) | Bangladesh | 2014 - 2015 | 2 | 19 | 34 | 32 | No | Thick & thin films | Yes | All slides checked by ≥2 microscopists |
| ZVYVK | Lidia-2015(183) | Indonesia | 2013 | 0 | 0 | 51 | 42 | Yes | Thick & thin: thick = parasites per 200 WBCs. Thin film = parasites per 2000 RBCs | No | N/A |
| AMNPY | Longley-2016(184) | Thailand | 2014 | 0 | 19 | 28 | 272 | No | Unclear if thick or thin | Yes | Parasites detected by expert microscopy |
| DGFPB | Marques-2014(185) | Brazil | 2007-2008 | 0 | 4 | 131 | 28 | Yes | Unclear if thick or thin | Yes | Parasite densities estimated by experienced microscopists |
| OGUXE | Mishra-2012(186) | India | 2009 - 2010 | 2 | 32 | 176 | 28 | Yes | Thick: parasites per 100 WBCs, WBCC of each patient used | Yes | All negative and 10% of positive slides checked by ≥2 microscopists |
| BGGDZ | Pasaribu-2013(187) | Indonesia | 2010 - 2012 | 26 | 148 | 157 | 378 | Yes | Thick: parasites per 200 WBCs, WBCC assumed 8000/μL | Yes | 10% of slides cross-checked in reference laboratory |
| YORDH | Pereira-2016(188) | Brazil | 2013 - 2014 | 0 | 0 | 86 | 32 | Yes | Thick: parasites per 200 WBCs | Yes | All slides checked by ≥2 microscopists |
| KLWVL | Phan-2002(189) | Vietnam | 1997 - 1999 | 0 | 0 | 227 | 28 | No | Unclear if thick or thin | Yes | All slides checked by ≥2 microscopists |
| FJCUS | Phyo-2011(190) | Thailand | 2007 - 2008 | 68 | 135 | 292 | 69 | Yes | Thick: parasites per undisclosed no. WBCs | No | N/A |
| XPFQR | Poespoprodjo-2018(191) | Indonesia | 2015 - 2016 | 1 | 21 | 46 | 42 | Yes | Thick & thin: parasites per 1000 RBCs, parasites per 500 WBCs if parasitaemia <1% | Yes | All slides checked by ≥2 microscopists |
| ASPIF | Poravuth-2011(192) | Multinational | 2007 - 2008 | 0 | 44 | 405 | 49 | Yes | Thick & thin: thick = parasites per 200 WBCs, WBCC assumed 7300/μL. Thin film if >200 parasites per 200 WBCs | Yes | All slides checked by ≥2 microscopists |
| GLNZB | Ratcliff-2007a(193) | Indonesia | 2004 | 15 | 9 | 19 | 42 | No | Thick: parasites per 200 WBCs if ≥10 parasites per 200 WBCs, parasites per 500 WBCs if <10 parasites per 200 WBCs | Yes | All slides checked by ≥2 microscopists |
| RCBGY | Ratcliff-2007b(194) | Indonesia | 2004 - 2005 | 33 | 49 | 93 | 56 | No | Thick & thin: thick = parasites per 200 WBCs, WBCC assumed 7300/μL. Thin film if >200 parasites per 200 WBCs | Yes | All slides checked by ≥2 microscopists |
| UOEHD | Rijal-2019(195) | Nepal | 2015 - 2016 | 0 | 13 | 193 | 389 | No | Thick & thin: thick = parasites per 200 WBCs, WBCC of each patient used. Thin film if >200 parasites per 200 WBCs | Yes | All slides checked by ≥2 microscopists |
| ZGBQY | Rishikesh-2015(196) | India | 2012 - 2015 | 0 | 0 | 117 | 28 | No | Thick & thin: WBCC assumed 8000/μL | Yes | Parasite densities taken as mean of 3 consecutive readings |
| VMMZX | Saravu-2016(197) | India | 2012 - 2014 | 0 | 0 | 161 | 212 | No | Thin: parasites per 200 WBCs if ≥10 parasites per 200 WBCs, parasites per 500 WBCs if <10 parasites per 200 WBCs, WBCC of each patient used | Yes | All slides checked by ≥2 microscopists |
| XAWRM | Siqueira-2016(198) | Brazil | 2012 - 2013 | 11 | 32 | 336 | 97 | Yes | Unclear if thick or thin: parasites per 200 WBCs if ≥10 parasites per 200 WBCs, parasites per 500 WBCs if <10 parasites per 200 WBCs, WBCC of each patient used | Yes | All slides checked by ≥2 microscopists |
| RYVHL | Sutanto-2009(199) | Indonesia | 2001 - 2002 | 9 | 20 | 2 | 28 | Yes | Unclear if thick or thin: parasites per undisclosed no. WBCs, WBCC of each patient used | Yes | All slides checked by ≥2 microscopists |
| JWUER | Sutanto-2010(200) | Indonesia | 2002 | 13 | 23 | 13 | 28 | Yes | Thick & thin: parasites per 200 WBCs, WBCC assumed 8000/μL | Yes | All slides checked by ≥2 microscopists |
| NVZAA | Taylor-2001(201) | Indonesia | 1995 - 1997 | 0 | 0 | 61 | 28 | No | Thick films | No | N/A |
| EOGVV | Taylor-2019(202) | Multinational | 2014 - 2017 | 154 | 837 | 1348 | 438 | No | Thick: parasites per 200 WBCs, WBCC assumed 8000/μL | Yes | 10% of slides cross-checked in reference laboratory |
| PCNGH | Thanh-2015(203) | Vietnam | 2009 - 2010 | 43 | 118 | 99 | 32 | No | Thick & thin: parasites per 200 WBCs, WBCC assumed 8000/μL | Yes | All slides checked by ≥2 microscopists |
| OMCLL | Thuan-2016(204) | Vietnam | 2013 - 2014 | 0 | 24 | 101 | 66 | Yes | Unclear if thick or thin: parasites per 500 WBCs or parasites per 1000 RBCs | Yes | All slides checked by ≥2 microscopists |
| IZIOR | Valecha-2006(205) | India | 2002 - 2003 | 9 | 28 | 250 | 28 | Yes | Thick & thin films | Yes | 10% of slides cross-checked |
| CFAGS | Wangchuk-2016(206) | Bhutan | 2013 - 2015 | 0 | 1 | 27 | 397 | No | Thick & thin films | Yes | All slides checked by ≥2 microscopists |
| CVIAE | Xu-2020(207) | Myanmar | 2014 - 2016 | 4 | 146 | 128 | 42 | No | Thick & thin: parasites per 200 WBCs, parasites per 500 WBCs if <99 parasites per 200 WBCs, WBCC assumed 8000/μL if age<5 or 5500/μL if age≥5 | Yes | All slides checked by ≥2 microscopists |
| ZVFMZ | Yuan-2015(208) | Myanmar | 2012 - 2013 | 64 | 373 | 151 | 42 | No | Thick & thin: parasites per 200 WBCs, WBCC assumed 8000/μL | Yes | All slides checked by ≥2 microscopists |
| MGZBE | Zuluaga-Idarraga-2016(209) | Colombia | 2012 - 2013 | 1 | 17 | 69 | 224 | Yes | Unclear if thick or thin: WHO guidance referenced but no further details | No | N/A |

WBC – white blood cell; WBCC – white blood cell count; RBC – red blood cell; RBCC – red blood cell count; WHO – World Health Organization

*These unpublished studies were not identified in the literature review. However, these data are held in the WWARN repository and met the study inclusion criteria and were therefore included in the analysis.

Table H: Study sites included in the analysis

| **Author-year** | **Country** | **Site name** | **Latitude** | **Longitude** | **Year start** | **Year end** | | **MAP incidence rate per 1000 (year of study)^a^** | | **Final transmission intensity classification** | **Region of relapse periodicity**(210) | | **Final category of relapse periodicity^b^** |
| --- | --- | --- | --- | --- | --- | --- | --- | --- | --- | --- | --- | --- | --- |
| Abdallah-2012(162) | Sudan | Kassala | 15 | 36 | 2011 | | 2011 | | 8 | Moderate | | 7 | Low |
| Abreha-2017(163) | Ethiopia | Batu | 7 | 39 | 2012 | | 2014 | | 68 | High | | 7 | Low |
| Abreha-2017(163) | Ethiopia | Bishoftu | 9 | 39 | 2012 | | 2014 | | 68 | High | | 7 | Low |
| Awab-2010(164) | Afghanistan | Jalalabad | 34 | 70 | 2007 | | 2009 | | 30 | High | | 11 | Low |
| Awab-2010(164) | Afghanistan | Maimana | 36 | 65 | 2007 | | 2009 | | 10 | Moderate | | 11 | Low |
| Awab-2010(164) | Afghanistan | Taloqan | 37 | 70 | 2007 | | 2009 | | 9 | Moderate | | 11 | Low |
| Barber-2013(165) | Malaysia | Sabah | 6 | 116 | 2010 | | 2015 | | 0 | Low | | 10 | High |
| Chu-2018(166) | Thailand | Mae Sot | 17 | 99 | 2010 | | 2012 | | 3 | Moderate | | 10 | High |
| Chu-2019(167) | Thailand | Mae Sot | 17 | 99 | 2012 | | 2015 | | 3 | Moderate | | 10 | High |
| Dao-2007(168) | Vietnam | Ho Chi Minh City | 11 | 107 | 2003 | | 2005 | | 0 | Low | | 10 | High |
| Dao-2007(168) | Vietnam | Truong Xuan | 15 | 109 | 2003 | | 2005 | | 0 | Low | | 10 | High |
| Getachew-2015(169) | Ethiopia | Arba Minch | 6 | 38 | 2010 | | 2013 | | 49 | High | | 7 | Low |
| Getachew-2015(169) | Ethiopia | Guba | 11 | 35 | 2010 | | 2013 | | 48 | High | | 7 | Low |
| Getachew-2015(169) | Ethiopia | Shone | 7 | 38 | 2010 | | 2013 | | 49 | High | | 7 | Low |
| Getachew-2015(169) | Ethiopia | Ziway | 8 | 39 | 2010 | | 2013 | | 68 | High | | 7 | Low |
| Gonzalez-Ceron-2015(170) | Mexico | Cacahoatan | 15 | -92 | 2008 | | 2020 | | 1 | Low | | 2 | Low |
| Gonzalez-Ceron-2015(170) | Mexico | Canton Nueva Esperanza | 15 | -92 | 2008 | | 2020 | | 0 | Low | | 2 | Low |
| Gonzalez-Ceron-2015(170) | Mexico | Fr Hidalgo | 15 | -92 | 2008 | | 2020 | | 1 | Low | | 2 | Low |
| Gonzalez-Ceron-2015(170) | Mexico | Huehuetan | 15 | -92 | 2008 | | 2020 | | 1 | Low | | 2 | Low |
| Gonzalez-Ceron-2015(170) | Mexico | Suchiate | 15 | -92 | 2008 | | 2020 | | 1 | Low | | 2 | Low |
| Gonzalez-Ceron-2015(170) | Mexico | Tapachula | 15 | -92 | 2008 | | 2020 | | 1 | Low | | 2 | Low |
| Gonzalez-Ceron-2015(170) | Mexico | Tuxtla Chico | 15 | -92 | 2008 | | 2020 | | 1 | Low | | 2 | Low |
| Gonzalez-Ceron-2015(170) | Mexico | Tuzantan | 15 | -92 | 2008 | | 2020 | | 1 | Low | | 2 | Low |
| Grigg-2016(171) | Malaysia | Kota Marudu | 6 | 117 | 2013 | | 2015 | | 0 | Low | | 10 | High |
| Grigg-2016(171) | Malaysia | Kudat | 7 | 117 | 2013 | | 2015 | | 0 | Low | | 10 | High |
| Grigg-2016(171) | Malaysia | Pitas | 7 | 117 | 2013 | | 2015 | | 0 | Low | | 10 | High |
| Guthmann-2008(172) | Myanmar | Dawei | 19 | 97 | 2002 | | 2003 | | 8 | Moderate | | 10 | High |
| Hasugian-2007(173) | Indonesia | Timika | -5 | 137 | 2005 | | 2005 | | 16 | High | | 12 | High |
| Hasugian-2009(174) | Indonesia | Timika | -5 | 137 | 2004 | | 2005 | | 16 | High | | 12 | High |
| Heidari-2012(175) | Iran | Sistan | -7 | 111 | 2010 | | 2010 | | 0 | Low | | 8 | Low |
| Hien-unpublished | Vietnam | Dak O | 12 | 108 | 2002 | | 2003 | | 0 | Low | | 10 | High |
| Hwang-2013(176) | Ethiopia | Bishoftu | 9 | 39 | 2009 | | 2010 | | 17 | High | | 7 | Low |
| Hwang-2013(176) | Ethiopia | Bulbula | 8 | 39 | 2009 | | 2010 | | 17 | High | | 7 | Low |
| Karunajeewa-2008(177) | Papua New Guinea | Kunjingini | -4 | 143 | 2005 | | 2005 | | 106 | High | | 12 | High |
| Karunajeewa-2008(177) | Papua New Guinea | Madang | -5 | 146 | 2005 | | 2005 | | 106 | High | | 12 | High |
| Karunajeewa-unpublished | Vanuatu | Luganville | -16 | 167 | 2013 | | 2013 | | 20 | High | | 12 | High |
| Karunajeewa-unpublished | Vanuatu | Nambauk | -15 | 167 | 2013 | | 2013 | | 20 | High | | 12 | High |
| Karunajeewa-unpublished | Vanuatu | Port Olry | -15 | 167 | 2013 | | 2013 | | 20 | High | | 12 | High |
| Ketema-2009(178) | Ethiopia | Serbo | 9 | 35 | 2007 | | 2007 | | 21 | High | | 7 | Low |
| Ladeia-Andrade-2019(179) | Brazil | Mancio Lima | -8 | -73 | 2014 | | 2015 | | 45 | High | | 3 | Low |
| Laman-2014(180) | Papua New Guinea | Madang | -5 | 146 | 2011 | | 2012 | | 56 | High | | 12 | High |
| Leslie-2007(181) | Afghanistan | Jalalabad | 34 | 70 | 2004 | | 2006 | | 30 | High | | 11 | Low |
| Leslie-2007(181) | Pakistan | Adizai | 34 | 72 | 2004 | | 2006 | | 2 | Moderate | | 8 | Low |
| Ley-2016(182) | Bangladesh | Alikadam Upazilla | 24 | 90 | 2014 | | 2015 | | 0 | Low | | 10 | High |
| Lidia-2015(183) | Indonesia | Kupang | -10 | 124 | 2013 | | 2013 | | 14 | High | | 10 | High |
| Longley-2016(184) | Thailand | Tha Song Yang | 18 | 98 | 2014 | | 2015 | | 1 | Low | | 10 | High |
| Marques-2014(185) | Brazil | Manaus | -3 | -60 | 2007 | | 2008 | | 77 | High | | 3 | Low |
| Mishra-2012(186) | India | Gulburga | 17 | 77 | 2009 | | 2010 | | 5 | Moderate | | 8 | Low |
| Mishra-2012(186) | India | Mangalore | 13 | 75 | 2009 | | 2010 | | 5 | Moderate | | 8 | Low |
| Mishra-2012(186) | India | North Goa | 16 | 74 | 2009 | | 2010 | | 8 | Moderate | | 8 | Low |
| Pasaribu-2013(187) | Indonesia | Tanjung Leidong | 3 | 100 | 2010 | | 2012 | | 0 | Low | | 10 | High |
| Pereira-2016(188) | Brazil | Rondonia | -12 | -64 | 2013 | | 2014 | | 10 | High | | 3 | Low |
| Phan-2002(189) | Vietnam | Binh Thuan | 11 | 107 | 1997 | | 1999 | | 0 | Low | | 10 | High |
| Phyo-2011(190) | Thailand | Mae Sot | 17 | 99 | 2007 | | 2008 | | 1 | Moderate | | 10 | High |
| Poespoprodjo-2018(191) | Indonesia | Timika | -5 | 137 | 2015 | | 2016 | | 6 | Moderate | | 12 | High |
| Poravuth-2011(192) | Cambodia | Pailin | 13 | 103 | 2007 | | 2008 | | 2 | Moderate | | 10 | High |
| Poravuth-2011(192) | India | Mangalore | 13 | 75 | 2007 | | 2008 | | 9 | Moderate | | 8 | Low |
| Poravuth-2011(192) | Indonesia | Maumere | -9 | 122 | 2007 | | 2008 | | 21 | High | | 10 | High |
| Poravuth-2011(192) | Thailand | Mae Ramat | 17 | 99 | 2007 | | 2008 | | 1 | Moderate | | 10 | High |
| Poravuth-2011(192) | Thailand | Mae Sot | 17 | 99 | 2007 | | 2008 | | 1 | Moderate | | 10 | High |
| Ratcliff-2007a(193) | Indonesia | Timika | -5 | 137 | 2004 | | 2004 | | 7 | Moderate | | 12 | High |
| Ratcliff-2007b(194) | Indonesia | Timika | -5 | 137 | 2004 | | 2005 | | 16 | High | | 12 | High |
| Rijal-2019(195) | Nepal | Jhapa | 27 | 88 | 2015 | | 2017 | | 0 | Low | | 10 | High |
| Rijal-2019(195) | Nepal | Kailali | 29 | 81 | 2015 | | 2017 | | 0 | Low | | 10 | High |
| Rishikesh-2015(196) | India | Kasturba | 13 | 75 | 2012 | | 2015 | | 3 | Moderate | | 8 | Low |
| Saravu-2016(197) | India | Udupi taluk | 13 | 75 | 2012 | | 2014 | | 2 | Moderate | | 8 | Low |
| Siqueira-2016(198) | Brazil | Manaus | -3 | -60 | 2012 | | 2013 | | 36 | High | | 3 | Low |
| Sutanto-2009(199) | Indonesia | Alor | -8 | 125 | 2001 | | 2002 | | 35 | High | | 10 | High |
| Sutanto-2010(200) | Indonesia | Lampung | -6 | 105 | 2002 | | 2002 | | 4 | Moderate | | 10 | High |
| Taylor-2001(201) | Indonesia | Jayapura | -3 | 141 | 1995 | | 1997 | | - | High | | 12 | High |
| Taylor-2019(202) | Afghanistan | Jalalabad | 34 | 70 | 2014 | | 2018 | | 41 | High | | 11 | Low |
| Taylor-2019(202) | Afghanistan | Laghman | 35 | 70 | 2014 | | 2018 | | 30 | High | | 11 | Low |
| Taylor-2019(202) | Ethiopia | Arba Minch | 6 | 38 | 2014 | | 2018 | | 20 | High | | 7 | Low |
| Taylor-2019(202) | Ethiopia | Metahara | 9 | 40 | 2014 | | 2018 | | 22 | High | | 7 | Low |
| Taylor-2019(202) | Indonesia | Hanura | -6 | 105 | 2014 | | 2018 | | 1 | Moderate | | 10 | High |
| Taylor-2019(202) | Indonesia | Tanjung Leidong | 3 | 100 | 2014 | | 2018 | | 0 | Low | | 10 | High |
| Taylor-2019(202) | Vietnam | Bu Gia Map | 12 | 107 | 2014 | | 2018 | | 0 | Low | | 10 | High |
| Taylor-2019(202) | Vietnam | Dak O | 12 | 108 | 2014 | | 2018 | | 0 | Low | | 10 | High |
| Taylor-2019(202) | Vietnam | Krong Pa | 13 | 109 | 2014 | | 2018 | | 0 | Low | | 10 | High |
| Thanh-2015(203) | Vietnam | Tra Leng | 15 | 108 | 2009 | | 2011 | | 0 | Low | | 10 | High |
| Thuan-2016(204) | Vietnam | Bu Gia Map | 12 | 107 | 2013 | | 2014 | | 1 | Low | | 10 | High |
| Thuan-2016(204) | Vietnam | Dak O | 12 | 108 | 2013 | | 2014 | | 1 | Low | | 10 | High |
| Valecha-2006(205) | India | Chennai | 13 | 80 | 2002 | | 2003 | | 4 | Moderate | | 8 | Low |
| Valecha-2006(205) | India | Gautam | 29 | 78 | 2002 | | 2003 | | 1 | Low | | 8 | Low |
| Valecha-2006(205) | India | Mumbai | 19 | 73 | 2002 | | 2003 | | 6 | Moderate | | 8 | Low |
| Wangchuk-2016(206) | Bhutan | Pemagatshel | 27 | 91 | 2013 | | 2015 | | 0 | Low | | 10 | High |
| Wangchuk-2016(206) | Bhutan | Samdrupjongkhar | 27 | 92 | 2013 | | 2015 | | 2 | Moderate | | 10 | High |
| Wangchuk-2016(206) | Bhutan | Sarpang | 27 | 90 | 2013 | | 2015 | | 0 | Low | | 10 | High |
| Wangchuk-2016(206) | Bhutan | Trongsa | 28 | 91 | 2013 | | 2015 | | 0 | Low | | 10 | High |
| Wangchuk-2016(206) | Bhutan | Tsirang | 27 | 90 | 2013 | | 2015 | | 0 | Low | | 10 | High |
| Wangchuk-2016(206) | Bhutan | Wangdiphodrang | 27 | 90 | 2013 | | 2015 | | 0 | Low | | 10 | High |
| Xu-2020(207) | Myanmar | Laiza | 25 | 98 | 2014 | | 2017 | | 2 | Moderate | | 10 | High |
| Yuan-2015(208) | Myanmar | Laiza | 25 | 98 | 2012 | | 2014 | | 5 | Moderate | | 10 | High |
| Zuluaga-Idarraga-2016(209) | Colombia | Turbo | 8 | -77 | 2012 | | 2013 | | 1 | Low | | 2 | Low |

^a^ *P. vivax* incidence per 1000 people estimated by the Malaria Atlas Project (MAP) for the median year of the study.

^b^ Low relapse periodicity ≤47 days.

| Table I: Parasite density distributions and centiles at enrolment and first recurrence by fever status | | | | | | | |
| --- | --- | --- | --- | --- | --- | --- | --- |
| **Category** | **Number of Patients** | **Median parasite density (IQR) (parasites /µL)** | **50^th^ centile (parasites /µL)** | **25^th^ centile (parasites /µL)** | **10^th^ centile (parasites /µL)** | **5^th^ centile (parasites /µL)** | **1^st^ centile (parasites /µL)** |
| ***A) Enrolment*** | **8,378** | **3,280 (968 – 8320)** | **3,280** | **968** | **278** | **120** | **26** |
| Age group |  |  |  |  |  |  |  |
| Age <5 years | 592 | 4,047 (1,240 – 10,578) | 4,047 | 1,240 | 368 | 188 | 38 |
| Age 5-<15 years | 3,051 | 3,400 (1,100 – 8,280) | 3,400 | 1,100 | 329 | 140 | 34 |
| Age ≥15 years | 4,734 | 3,087 (864 – 8,040) | 3,087 | 864 | 240 | 100 | 23 |
| Relapse periodicity region |  |  |  |  |  |  |  |
| Low relapse periodicity | 3,484 | 3,444 (1,204 – 8,000) | 3,444 | 1,204 | 504 | 271 | 82 |
| High relapse periodicity | 4,894 | 3,160 (760 – 8,774) | 3,160 | 760 | 185 | 77 | 21 |
| ***B) Febrile 1^st^ recurrence*** | **849** | **3,926 (1,019 - 9,744)** | **3,926** | **1,019** | **152** | **56** | **20** |
| Age group |  |  |  |  |  |  |  |
| Age <5 years | 103 | 5,046 (940 - 12,434) | 5,046 | 940 | 199 | 75 | 33 |
| Age 5-<15 years | 280 | 4,572 (1,027 - 10,020) | 4,572 | 1,027 | 150 | 60 | 16 |
| Age ≥15 years | 466 | 3,317 (1,063 - 9,035) | 3,317 | 1,063 | 150 | 41 | 22 |
| Relapse periodicity |  |  |  |  |  |  |  |
| Low relapse periodicity | 360 | 4,332 (1,336 - 9,660) | 4,332 | 1,336 | 481 | 220 | 74 |
| High relapse periodicity | 489 | 3,752 (719 - 9,912) | 3,752 | 719 | 66 | 33 | 17 |
| ***C) Afebrile 1^st^ recurrence*** | **1,134** | **434 (93 – 2,480)** | **434** | **93** | **33** | **22** | **15** |
| Age group |  |  |  |  |  |  |  |
| Age <5 years | 227 | 515 (112 – 3,674) | 515 | 112 | 40 | 32 | 17 |
| Age 5-<15 years | 324 | 396 (98 – 1,987) | 396 | 98 | 32 | 16 | 11 |
| Age ≥15 years | 583 | 437 (75 – 2,500) | 437 | 75 | 32 | 20 | 15 |
| Relapse periodicity |  |  |  |  |  |  |  |
| Low relapse periodicity | 237 | 860 (322 – 4,019) | 860 | 322 | 140 | 71 | 40 |
| High relapse periodicity | 897 | 312 (69 – 2,112) | 312 | 69 | 32 | 17 | 11 |

Figure A: Parasite density distributions at enrolment for each study by age group and relapse periodicity


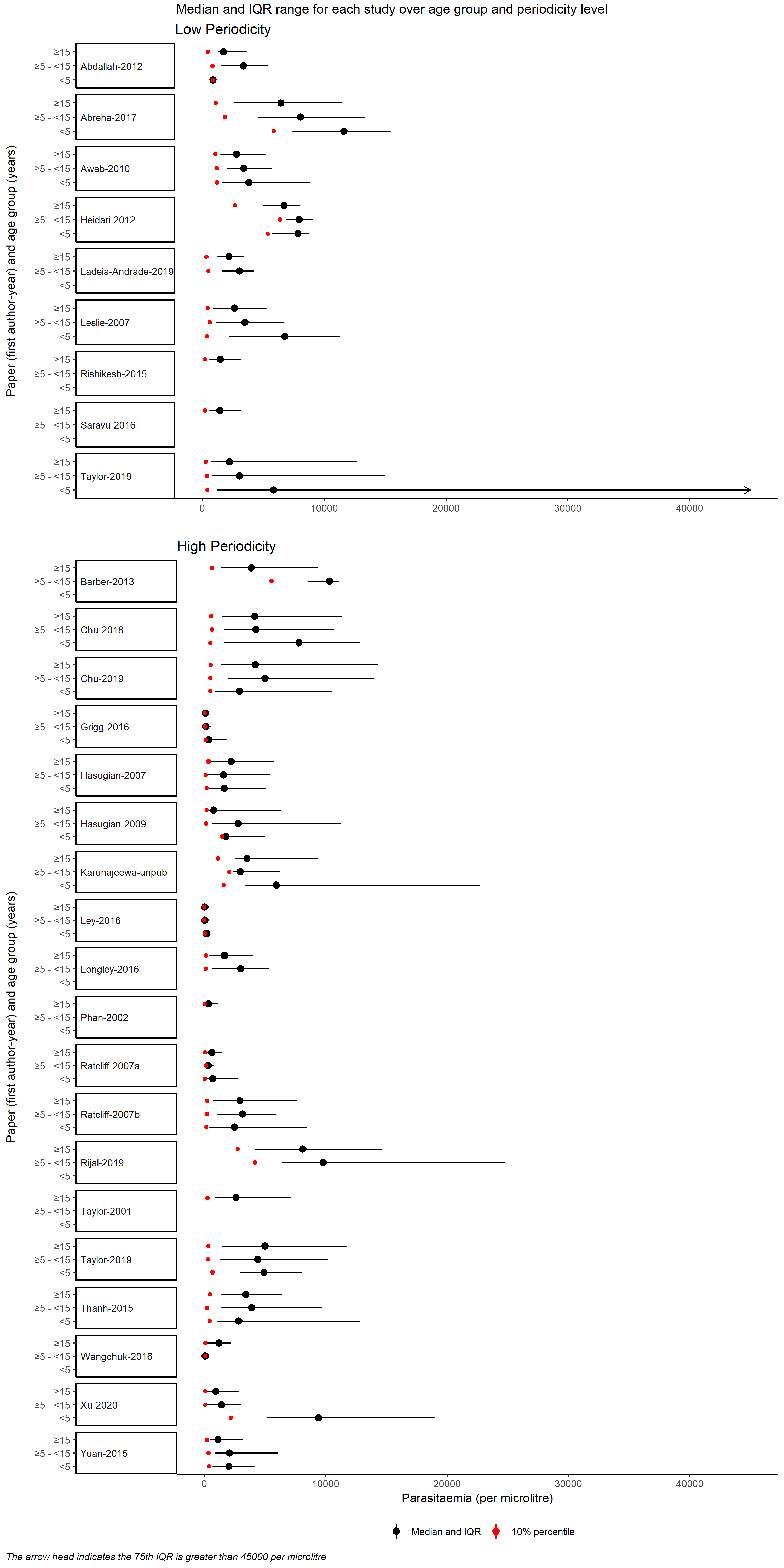


The arrowhead indicates the 75^th^ percentile is greater than 45,000 parasites per microlitre. Some multisite studies are recorded for both high and low relapse periodicity regions.


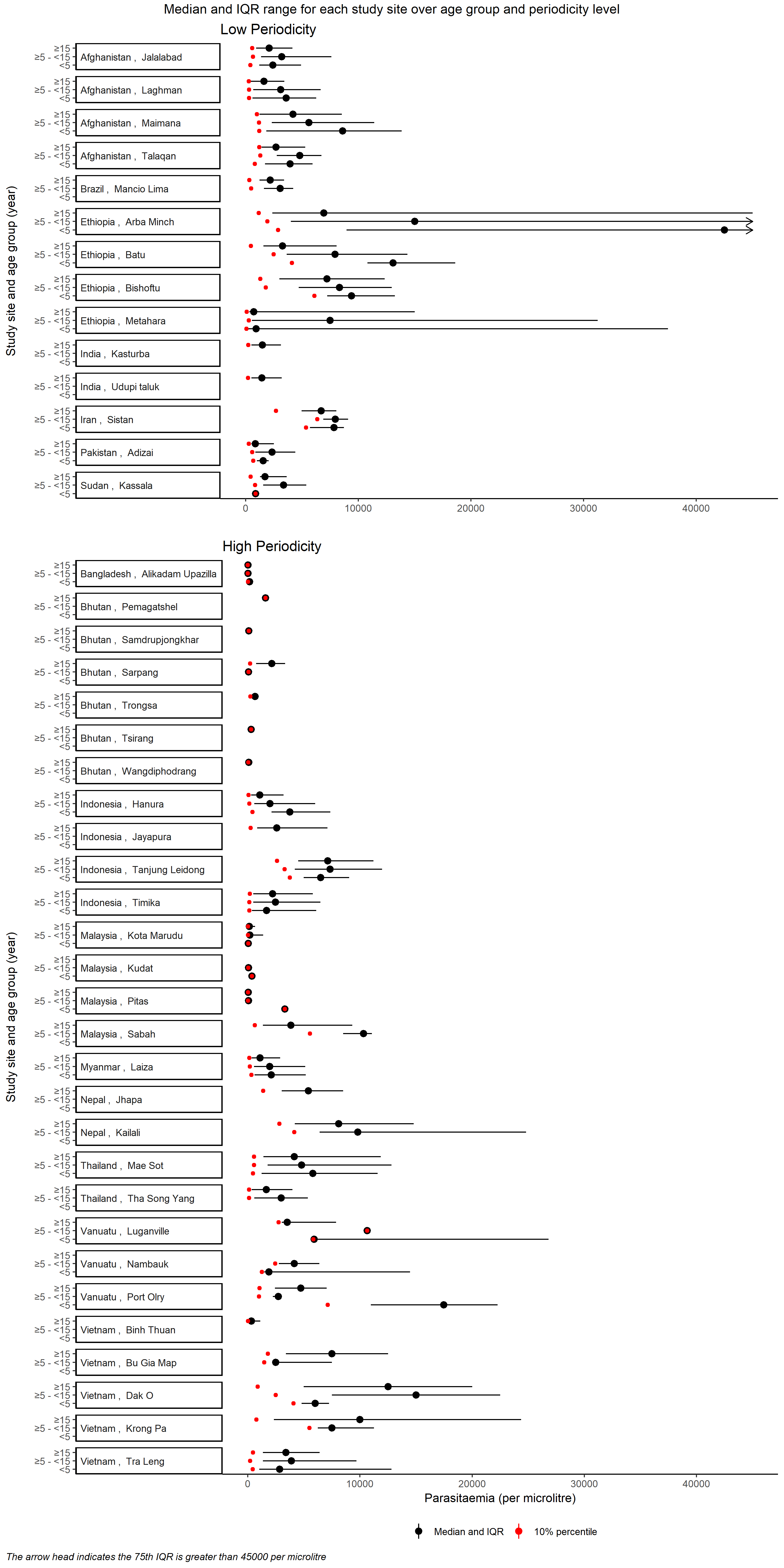
Figure B: Parasite density distributions at enrolment for each study site by age group and relapse periodicity

The arrowhead indicates the 75^th^ percentile is greater than 45,000 parasites per microlitre. Some multisite studies are recorded for both high and low relapse periodicity regions.

Table J: Parasite density distributions and centiles for febrile patients at enrolment by relapse periodicity and age

| **Category** | **Number of Patients** | **Median parasite density (IQR) (parasites /µL)** | **Absolute IQR (parasites /µL)** | **50^th^ centile (parasites /µL)** | **25^th^ centile (parasites /µL)** | **10^th^ centile (parasites /µL)** | **5^th^ centile (parasites /µL)** | **1^st^ centile (parasites /µL)** |
| --- | --- | --- | --- | --- | --- | --- | --- | --- |
| Low relapse periodicity | | | | | | | | |
| Age <5 years | 229 | 6,069 (1,880 – 13,680) | 11,880 | 6,069 | 1,880 | 760 | 370 | 75 |
| Age 5-<15 years | 1,440 | 3,827 (1,430 – 8,320) | 6,890 | 3,827 | 1,431 | 624 | 352 | 104 |
| Age ≥15 years | 1,814 | 2,963 (1,093 – 7,200) | 6,107 | 2,963 | 1,093 | 400 | 210 | 64 |
| High relapse periodicity | | | | | | | | |
| Age <5 years | 363 | 3,318 (832 – 8,740) | 7,908 | 3,318 | 832 | 260 | 150 | 38 |
| Age 5-<15 years | 1,611 | 3,080 (852 – 8,211) | 7,359 | 3,080 | 852 | 189 | 81 | 24 |
| Age ≥15 years | 2,920 | 3,200 (714 – 9,084) | 8,370 | 3,200 | 714 | 170 | 65 | 16 |

Figure C: Receiver operating characteristic curve depicting three thresholds (parasites/µL) including the pyrogenic density at first recurrence


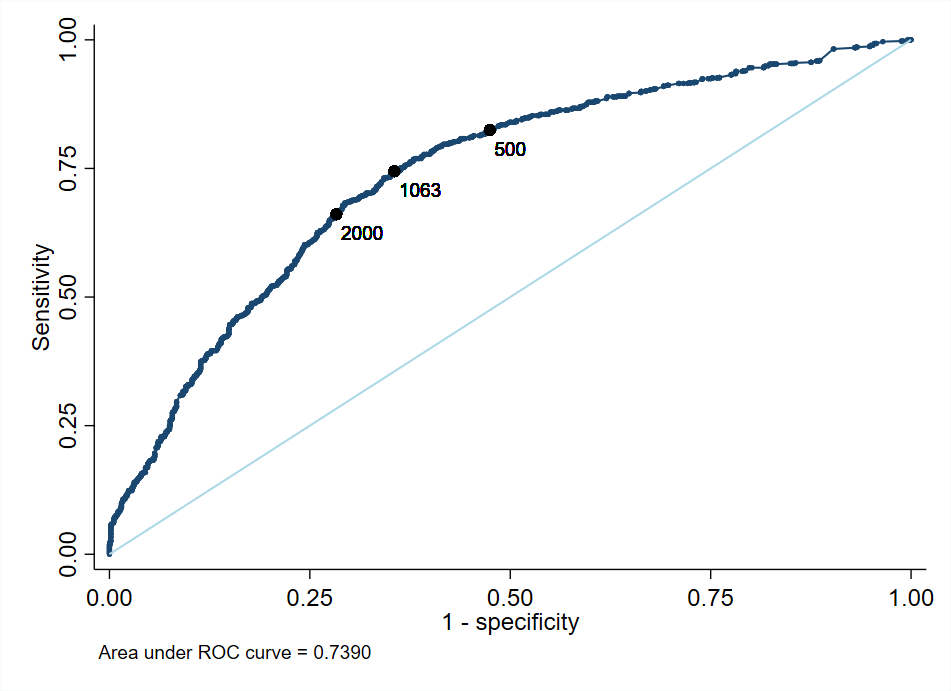


Table K: Risk factors for fever at first *P. vivax* parasite recurrence

| **Variable** | **Fever / Total patients (%)** | **OR (95% CI)** | **p value** |
| --- | --- | --- | --- |
| Baseline parasite density (/µL, per 10-fold increase) | 849/1,983 (42.8%) | 2.77 (2.00, 3.84) | <0.0001 |
| *Age group* |  |  |  |
| <5 years | 103/330 (31.2%) | 0.57 (0.25, 1.27) | 0.167 |
| 5 - <15 years | 280/604 (46.4%) | 1.08 (0.70, 1.66) | 0.721 |
| ≥15 years | 466/1,049 (44.4%) | Reference | - |
| *Sex* |  |  |  |
| Male | 552/1,282 (43.1%) | Reference | - |
| Female | 297/701 (42.4%) | 0.97 (0.78, 1.22) | 0.807 |
| *Relapse periodicity region* |  |  |  |
| Low | 360/597 (60.3%) | Reference | - |
| High | 489/1,386 (35.3%) | 0.36 (0.14, 0.90) | 0.029 |
| *Schizonticidal half-life* |  |  |  |
| Rapidly eliminated | 23/180 (12.8%) | 0.18 (0.11, 0.29) | <0.0001 |
| Intermediately eliminated | 105/199 (52.8%) | 1.37 (0.48, 3.90) | 0.558 |
| Slowly eliminated | 721/1,604 (45.0%) | Reference | - |
| *Timing of recurrence after initial episode* |  |  |  |
| >90 days | 257/428 (60.0%) | Reference | - |
| ≤90 days | 592/1,555 (38.1%) | 0.41 (0.24, 0.70) | 0.001 |

References

1. Commons RJ, Thriemer K, Humphreys G, Suay I, Sibley CH, Guerin PJ, et al. The vivax surveyor: online mapping database for Plasmodium vivax clinical trials. Int J Parasitol Drugs Drug Resist. 2017;7(2):181–90.

2. Das S, Padhi S. Prevalence of Clinical Artesunate Resistance among Cases of Complicated Malaria in Southern Odisha, India. J Clin Diagn Res [Internet]. 2020 [cited 2023 Jul 17]; Available from: https://jcdr.net/article_fulltext.asp?issn=0973-709x&year=2020&volume=14&issue=9&page=DC10&issn=0973-709x&id=14016

3. McGready R, Thwai KL, Cho T, Samuel, Looareesuwan S, White NJ, et al. The effects of quinine and chloroquine antimalarial treatments in the first trimester of pregnancy. Trans R Soc Trop Med Hyg. 2002/06/12 ed. 2002 Mar;96(2):180–4.

4. Moore BR, Benjamin JM, Auyeung SO, Salman S, Yadi G, Griffin S, et al. Safety, tolerability and pharmacokinetic properties of coadministered azithromycin and piperaquine in pregnant Papua New Guinean women: Pharmacokinetics of AZI plus PQP in pregnancy. Br J Clin Pharmacol. 2016 Jul;82(1):199–212.

5. Kim C, Choi JY, Park I, Kim YK, Kim JM, Kim HY. Therapeutic Efficacy of Chloroquine in Plasmodium vivax and the pvmdr1 Polymorphisms in the Republic of Korea Under Mass Chemoprophylaxis. Am J Trop Med Hyg. 2011 Apr 5;84(4):532–4.

6. Congpuong K, Bualombai P, Banmairuroi V, Na-Bangchang K. Compliance with a three-day course of artesunate-mefloquine combination and baseline anti-malarial treatment in an area of Thailand with highly multidrug resistant falciparum malaria. Malar J. 2010/02/06 ed. 2010 Feb 4;9:43.

7. Grigg MJ, William T, Barber BE, Rajahram GS, Menon J, Schimann E, et al. Age-Related Clinical Spectrum of Plasmodium knowlesi Malaria and Predictors of Severity. Clin Infect Dis. 2018/06/07 ed. 2018 Jul 18;67(3):350–9.

8. Alecrim MG, Carvalho LM, Fernandes MC, de Andrade SD, Loureiro AC, Arcanjo AR, et al. [Malaria treatment with artesunate (retocaps) in children of the Brazilian Amazon]. Rev Soc Bras Med Trop. 2000/07/06 ed. 2000 Mar;33(2):163–8.

9. Barnadas C, Tichit M, Bouchier C, Ratsimbasoa A, Randrianasolo L, Raherinjafy R, et al. Plasmodium vivax dhfr and dhps mutations in isolates from Madagascar and therapeutic response to sulfadoxine-pyrimethamine. Malar J. 2008;7:35–35.

10. Fernandopulle BM, Weeraratne CL, Weerasuriya K, Karunaweera ND. Efficacy of a five-day course of primaquine in preventing relapses in Plasmodium vivax malaria--a pilot study. Ceylon Med J. 2003/06/11 ed. 2003 Mar;48(1):32.

11. Hamedi Y, Safa O, Zare S, Tan-ariya P, Kojima S, Looareesuwan S. Therapeutic efficacy of artesunate in Plasmodium vivax malaria in Thailand. Southeast Asian J Trop Med Public Health. 2004;35(3):570–4.

12. Hamid MMA, Thriemer K, Elobied ME, Mahgoub NS, Boshara SA, Elsafi HMH, et al. Low risk of recurrence following artesunate-Sulphadoxine-pyrimethamine plus primaquine for uncomplicated Plasmodium falciparum and Plasmodium vivax infections in the Republic of the Sudan. Malar J. 20180316th ed. 2018 Mar 16;17(1):117.

13. Khan MZ, Isani Z, Ahmed TM, Zafar AB, Gilal N, Maqbool S, et al. Efficacy and safety of halofantrine in Pakistani children and adults with malaria caused by P. falciparum and P. vivax. Southeast Asian J Trop Med Public Health. 2006/11/24 ed. 2006 Jul;37(4):613–8.

14. Kheng S, Muth S, Taylor WR, Tops N, Kosal K, Sothea K, et al. Tolerability and safety of weekly primaquine against relapse of Plasmodium vivax in Cambodians with glucose-6-phosphate dehydrogenase deficiency. BMC Med. 2015/08/26 ed. 2015 Aug 25;13:203.

15. Krudsood S, Tangpukdee N, Wilairatana P, Phophak N, Baird JK, Brittenham GM, et al. High-dose primaquine regimens against relapse of Plasmodium vivax malaria. Am J Trop Med Hyg. 2008;78(5):736–40.

16. Lacy MD, Maguire JD, Barcus MJ, Ling J, Bangs MJ, Gramzinski R, et al. Atovaquone/proguanil therapy for Plasmodium falciparum and Plasmodium vivax malaria in Indonesians who lack clinical immunity. Clin Infect Dis. 2002/10/18 ed. 2002 Nov 1;35(9):e92-5.

17. Llanos-Cuentas A, Casapia M, Chuquiyauri R, Hinojosa JC, Kerr N, Rosario M, et al. Antimalarial activity of single-dose DSM265, a novel plasmodium dihydroorotate dehydrogenase inhibitor, in patients with uncomplicated Plasmodium falciparum or Plasmodium vivax malaria infection: a proof-of-concept, open-label, phase 2a study. Lancet Infect Dis. 2018 Aug;18(8):874–83.

18. Pukrittayakamee S, Clemens R, Chantra A, Nontprasert A, Luknam T, Looareesuwan S, et al. Therapeutic responses to antibacterial drugs in vivax malaria. Trans R Soc Trop Med Hyg. 2001/11/15 ed. 2001 Sep;95(5):524–8.

19. Silachamroon U, Krudsood S, Treeprasertsuk S, Wilairatana P, Chalearmrult K, Mint HY, et al. Clinical trial of oral artesunate with or without high-dose primaquine for the treatment of vivax malaria in Thailand. Am J Trop Med Hyg. 2003;69(1):14–8.

20. Tavul L, Hetzel MW, Teliki A, Walsh D, Kiniboro B, Rare L, et al. Efficacy of artemether–lumefantrine and dihydroartemisinin–piperaquine for the treatment of uncomplicated malaria in Papua New Guinea. Malar J. 2018 Dec;17(1):350.

21. Yeramian P, Meshnick SR, Krudsood S, Chalermrut K, Silachamroon U, Tangpukdee N, et al. Efficacy of DB289 in Thai patients with Plasmodium vivax or acute, uncomplicated Plasmodium falciparum infections. J Infect Dis. 2005/06/18 ed. 2005 Jul 15;192(2):319–22.

22. Marfurt J, Mueller I, Sie A, Maku P, Goroti M, Reeder JC, et al. Low efficacy of amodiaquine or chloroquine plus sulfadoxine-pyrimethamine against Plasmodium falciparum and P. vivax malaria in Papua New Guinea. Am J Trop Med Hyg. 2007/11/07 ed. 2007 Nov;77(5):947–54.

23. Phyo AP, Jittamala P, Nosten FH, Pukrittayakamee S, Imwong M, White NJ, et al. Antimalarial activity of artefenomel (OZ439), a novel synthetic antimalarial endoperoxide, in patients with Plasmodium falciparum and Plasmodium vivax malaria: an open-label phase 2 trial. Lancet Infect Dis. 2015/10/09 ed. 2016 Jan;16(1):61–9.

24. White NJ, Duong TT, Uthaisin C, Nosten F, Phyo AP, Hanboonkunupakarn B, et al. Antimalarial Activity of KAF156 in Falciparum and Vivax Malaria. N Engl J Med. 2016/09/23 ed. 2016 Sep 22;375(12):1152–60.

25. Abdon NP, Pinto AY, das Silva Rdo S, de Souza JM. [Assessment of the response to reduced treatment schemes for vivax malaria]. Rev Soc Bras Med Trop. 2001/09/20 ed. 2001 Jul;34(4):343–8.

26. Adak T, Valecha N, Sharma VP. Plasmodium vivax polymorphism in a clinical drug trial. Clin Diagn Lab Immunol. 2001/08/31 ed. 2001 Sep;8(5):891–4.

27. Ould Ahmedou Salem MS, Mohamed Lemine YO, Deida JM, Lemrabott MA, Ouldabdallahi M, Ba MD, et al. Efficacy of chloroquine for the treatment of Plasmodium vivax in the Saharan zone in Mauritania. Malar J. 2015/01/30 ed. 2015 Jan 28;14:39.

28. Amaratunga C, Sreng S, Mao S, Tullo GS, Anderson JM, Chuor CM, et al. Chloroquine remains effective for treating Plasmodium vivax malaria in Pursat province, Western Cambodia. Antimicrob Agents Chemother. 2014/07/23 ed. 2014 Oct;58(10):6270–2.

29. Anez A, Navarro-Costa D, Yucra O, Garnica C, Melgar V, Moscoso M, et al. [Therapeutic response of Plasmodium vivax to chloroquine in Bolivia]. Biomedica. 2013/05/30 ed. 2012 Oct;32(4):527–35.

30. Asih PB, Syafruddin D, Leake J, Sorontou Y, Sadikin M, Sauerwein RW, et al. Phenotyping clinical resistance to chloroquine in Plasmodium vivax in northeastern Papua, Indonesia. Int J Parasitol Drugs Drug Resist. 2011/12/01 ed. 2011 Dec;1(1):28–32.

31. Assefa M, Eshetu T, Biruksew A. Therapeutic efficacy of chloroquine for the treatment of Plasmodium vivax malaria among outpatients at Hossana Health Care Centre, southern Ethiopia. Malar J. 2015/11/19 ed. 2015 Nov 17;14:458.

32. Awab GR, Imwong M, Bancone G, Jeeyapant A, Day NPJ, White NJ, et al. Chloroquine-Primaquine versus Chloroquine Alone to Treat Vivax Malaria in Afghanistan: An Open Randomized Superiority Trial. Am J Trop Med Hyg. 2017/11/17 ed. 2017 Dec;97(6):1782–7.

33. Azarian Moghadam HN, Raeisi M, Motevalli Haghi A, Edrissian A, Farivar G. Monitoring the Response of Plasmodium vivax to Chloroquine and Uncomplicated P. falciparum to Artesunate-fansidar Antimalarials in Southeastern Iran. Iran J Parasitol. 2018;13(1):31–8.

34. Baird JK, Tiwari T, Martin GJ, Tamminga CL, Prout TM, Tjaden J, et al. Chloroquine for the treatment of uncomplicated malaria in Guyana. Ann Trop Med Parasitol. 2002/08/13 ed. 2002 Jun;96(4):339–48.

35. Barnadas C, Ratsimbasoa A, Tichit M, Bouchier C, Jahevitra M, Picot S, et al. Plasmodium vivax resistance to chloroquine in Madagascar: clinical efficacy and polymorphisms in pvmdr1 and pvcrt-o genes. Antimicrob Agents Chemother. 2008;52(12):4233–40.

36. Benjamin J, Moore B, Lee ST, Senn M, Griffin S, Lautu D, et al. Artemisinin-naphthoquine combination therapy for uncomplicated pediatric malaria: a tolerability, safety, and preliminary efficacy study. Antimicrob Agents Chemother. 2012/02/15 ed. 2012 May;56(5):2465–71.

37. Bergonzoli G, Rivers Cuadra JC. [Therapeutic efficacy of different antimalarial regimens in the Costa Rica-Nicaragua border region]. Rev Panam Salud Publica. 2000/08/19 ed. 2000 Jun;7(6):366–70.

38. Betuela I, Rosanas-Urgell A, Kiniboro B, Stanisic DI, Samol L, De Lazzari E, et al. Relapses contribute significantly to the risk of Plasmodium vivax infection and disease in Papua New Guinean children 1-5 years of age. J Infect Dis. 2012;206(11):1771–80.

39. Beyene HB, Beyene MB, Ebstie YA, Desalegn Z. Efficacy of Chloroquine for the Treatment of Vivax malaria in Northwest Ethiopia. PLoS One. 2016/09/01 ed. 2016;11(8):e0161483.

40. Blair-Trujillo S, Castano AT, Restrepo ME, Sanchez GA, Carmona-Fonseca J. [Adequate clinical and parasitological Plasmodium vivax response to chloroquine in Colombia (Turbo, Antioquia), 2001]. Infectio. 2002;6(1):21–6.

41. Brasil LW, Rodrigues-Soares F, Santoro AB, Almeida ACG, Kühn A, Ramasawmy R, et al. CYP2D6 activity and the risk of recurrence of Plasmodium vivax malaria in the Brazilian Amazon: a prospective cohort study. Malar J. 2018 Dec;17(1):57.

42. Buchachart K, Krudsood S, Singhasivanon P, Treeprasertsuk S, Phophak N, Srivilairit S, et al. Effect of primaquine standard dose (15 mg/day for 14 days) in the treatment of vivax malaria patients in Thailand. Southeast Asian J Trop Med Public Health. 2001;32(4):720–6.

43. Carmona-Fonseca J, Álvarez G, Blair S. Malaria por Plasmodium vivax: curación del ataque agudo con tres dosis diferentes de primaquina y dosis fija de cloroquina. Antioquia, Colombia, 2003-2004. Biomédica. 2006 Sep 1;26(3):353.

44. Carmona-Fonseca J, Maestre A. Prevention of Plasmodium vivax malaria recurrence: efficacy of the standard total dose of primaquine administered over 3 days. Acta Trop. 2009;112(2):188–92.

45. Carmona-Fonseca J. Vivax malaria in children: Recurrences with standard total dose of primaquine administered in 3 vs. 7 days. Iatreia. 2010;23(1):10–20.

46. Castillo CM, Osorio LE, Palma GI. Assessment of therapeutic response of Plasmodium vivax and Plasmodium falciparum to chloroquine in a Malaria transmission free area in Colombia. Mem Inst Oswaldo Cruz. 2002/07/16 ed. 2002 Jun;97(4):559–62.

47. Cheoymang A, Ruenweerayut R, Muhamad P, Rungsihirunrat K, Na-Bangchang K. Patients’ adherence and clinical effectiveness of a 14-day course of primaquine when given with a 3-day chloroquine in patients with Plasmodium vivax at the Thai-Myanmar border. Acta Trop. 2015/08/19 ed. 2015 Dec;152:151–6.

48. Congpuong K, Na-Bangchang K, Thimasarn K, Tasanor U, Wernsdorfer WH. Sensitivity of Plasmodium vivax to chloroquine in Sa Kaeo Province, Thailand. Acta Trop. 2002/06/29 ed. 2002 Aug;83(2):117–21.

49. Congpuong K, Satimai W, Sujariyakul A, Intanakom S, Harnpitakpong W, Pranuth Y, et al. In vivo sensitivity monitoring of chloroquine for the treatment of uncomplicated vivax malaria in four bordered provinces of Thailand during 2009–2010. J Vector Borne Dis. 2011;

50. da Silva RSU, Pinto AYN, Calvosa VSP, de Souza JM. [Short course schemes for vivax malaria treatment]. Rev Soc Bras Med Trop. 2003;36(2):235–9.

51. Daneshvar C, Davis TM, Cox-Singh J, Rafa’ee MZ, Zakaria SK, Divis PC, et al. Clinical and parasitological response to oral chloroquine and primaquine in uncomplicated human Plasmodium knowlesi infections. Malar J. 2010;9(1):238–238.

52. de Santana Filho FS, Arcanjo AR, Chehuan YM, Costa MR, Martinez-Espinosa FE, Vieira JL, et al. Chloroquine-resistant Plasmodium vivax, Brazilian Amazon. Emerg Infect Dis. 2007;13(7):1125–6.

53. Delgado-Ratto C, Soto-Calle VE, Van den Eede P, Gamboa D, Rosas A, Abatih EN, et al. Population structure and spatio-temporal transmission dynamics of Plasmodium vivax after radical cure treatment in a rural village of the Peruvian Amazon. Malar J. 2014/01/08 ed. 2014 Jan 6;13:8.

54. Dharmawardena P, Rodrigo C, Mendis K, de AWGW, Premaratne R, Ringwald P, et al. Response of imported malaria patients to antimalarial medicines in Sri Lanka following malaria elimination. PLoS One. 2017/11/29 ed. 2017;12(11):e0188613.

55. Dilmec F, Kurcer MA, Akkafa F, Simsek Z. Monitoring of failure of chloroquine treatment for Plasmodium vivax using polymerase chain reaction in Sanliurfa province, Turkey. Parasitol Res. 2010;106(4):783–8.

56. Dua VK, Sharma VP. Plasmodium vivax relapses after 5 days of primaquine treatment, in some industrial complexes of India. Ann Trop Med Parasitol. 2002/01/11 ed. 2001 Oct;95(7):655–9.

57. Duarte EC, Pang LW, Ribeiro LC, Fontes CJ. Association of subtherapeutic dosages of a standard drug regimen with failures in preventing relapses of vivax malaria. Am J Trop Med Hyg. 2001 Nov;65(5):471–6.

58. Dunne MW, Singh N, Shukla M, Valecha N, Bhattacharyya PC, Patel K, et al. A double-blind, randomized study of azithromycin compared to chloroquine for the treatment of Plasmodium vivax malaria in India. Am J Trop Med Hyg. 2005/12/16 ed. 2005 Dec;73(6):1108–11.

59. Eibach D, Ceron N, Krishnalall K, Carter K, Bonnot G, Bienvenu AL, et al. Therapeutic efficacy of artemether-lumefantrine for Plasmodium vivax infections in a prospective study in Guyana. Malar J. 2012/10/23 ed. 2012 Oct 19;11:347.

60. Fryauff DJ, Leksana B, Masbar S, Wiady I, Sismadi P, Susanti AI, et al. The drug sensitivity and transmission dynamics of human malaria on Nias Island, North Sumatra, Indonesia. Ann Trop Med Parasitol. 2002/08/27 ed. 2002 Jul;96(5):447–62.

61. Fukuda MM, Krudsood S, Mohamed K, Green JA, Warrasak S, Noedl H, et al. A randomized, double-blind, active-control trial to evaluate the efficacy and safety of a three day course of tafenoquine monotherapy for the treatment of Plasmodium vivax malaria. PLoS One. 2017/11/10 ed. 2017;12(11):e0187376.

62. Ganguly S, Saha P, Guha SK, Das S, Bera DK, Biswas A, et al. In vivo therapeutic efficacy of chloroquine alone or in combination with primaquine against vivax malaria in Kolkata, West Bengal, India, and polymorphism in pvmdr1 and pvcrt-o genes. Antimicrob Agents Chemother. 2012/12/25 ed. 2013 Mar;57(3):1246–51.

63. Genton B, Baea K, Lorry K, Ginny M, Wines B, Alpers MP. Parasitological and clinical efficacy of standard treatment regimens against Plasmodium falciparum, P. vivax and P. malariae in Papua New Guinea. P N G Med J. 2007/01/11 ed. 2005 Sep;48(3–4):141–50.

64. Graf PC, Durand S, Alvarez Antonio C, Montalvan C, Galves Montoya M, Green MD, et al. Failure of Supervised Chloroquine and Primaquine Regimen for the Treatment of Plasmodium vivax in the Peruvian Amazon. Malar Res Treat. 2012/06/16 ed. 2012;2012:936067.

65. Hamedi Y, Nateghpour M, Tan-ariya P, Tiensuwan M, Silachamroon U, Looareesuwan S. Plasmodium vivax malaria in Southeast Iran in 1999-2001: establishing the response to chloroquine in vitro and in vivo. Southeast Asian J Trop Med Public Health. 2003/04/16 ed. 2002 Sep;33(3):512–8.

66. Han KT, Lin K, Han ZY, Myint MK, Aye KH, Thi A, et al. Efficacy and Safety of Pyronaridine–Artesunate for the Treatment of Uncomplicated Plasmodium falciparum and Plasmodium vivax Malaria in Myanmar. Am J Trop Med Hyg. 2020 Sep 2;103(3):1088–93.

67. Hapuarachchi HA, Dayanath MY, Abeysundara S, Bandara KB, Abeyewickreme W, de Silva NR. Chloroquine resistant falciparum malaria among security forces personnel in the Northern Province of Sri Lanka. Ceylon Med J. 2004/09/01 ed. 2004 Jun;49(2):47–51.

68. Htun MW, Mon NCN, Aye KM, Hlaing CM, Kyaw MP, Handayuni I, et al. Chloroquine efficacy for Plasmodium vivax in Myanmar in populations with high genetic diversity and moderate parasite gene flow. Malar J. 2017/07/12 ed. 2017 Jul 10;16(1):281.

69. Ketema T, Getahun K, Bacha K. Therapeutic efficacy of chloroquine for treatment of Plasmodium vivax malaria cases in Halaba district, South Ethiopia. Parasit Vectors. 2011;4(1):46–46.

70. Kinzer MH, Chand K, Basri H, Lederman ER, Susanti AI, Elyazar I, et al. Active case detection, treatment of falciparum malaria with combined chloroquine and sulphadoxine/pyrimethamine and vivax malaria with chloroquine and molecular markers of anti-malarial resistance in the Republic of Vanuatu. Malar J. 2010;9(1):89–89.

71. Kolaczinski K, Durrani N, Rahim S, Rowland M. Sulfadoxine-pyrimethamine plus artesunate compared with chloroquine for the treatment of vivax malaria in areas co-endemic for Plasmodium falciparum and P. vivax: a randomised non-inferiority trial in Eastern Afghanistan. Trans R Soc Trop Med Hyg. 2007;101(11):1081–7.

72. Krudsood S, Wilairatana P, Tangpukdee N, Chalermrut K, Srivilairit S, Thanachartwet V, et al. Safety and tolerability of elubaquine (bulaquine, CDRI 80/53) for treatment of Plasmidium vivax malaria in Thailand. Korean J Parasitol. 2006;44(3):221–8.

73. Krudsood S, Tangpukdee N, Muangnoicharoen S, Thanachartwet V, Luplertlop N, Srivilairit S, et al. Clinical efficacy of chloroquine versus artemether-lumefantrine for Plasmodium vivax treatment in Thailand. Korean J Parasitol. 2007;45(2):111–4.

74. Kumar R, Guddattu V, Saravu K. Therapeutic Assessment of Primaquine for Radical Cure of Plasmodium vivax Malaria at Primary and Tertiary Care Centres in Southwestern India. Korean J Parasitol. 2016 Dec 31;54(6):733–42.

75. Kurcer MA, Simsek Z, Zeyrek FY, Atay S, Celik H, Kat I, et al. Efficacy of chloroquine in the treatment of Plasmodium vivax malaria in Turkey. Ann Trop Med Parasitol. 2004;98(5):447–51.

76. Kurcer MA, Simsek Z, Kurcer Z. The decreasing efficacy of chloroquine in the treatment of Plasmodium vivax malaria, in Sanliurfa, south-eastern Turkey. Ann Trop Med Parasitol. 2006/02/24 ed. 2006 Mar;100(2):109–13.

77. Lacerda MVG, Llanos-Cuentas A, Krudsood S, Lon C, Saunders DL, Mohammed R, et al. Single-Dose Tafenoquine to Prevent Relapse of Plasmodium vivax Malaria. N Engl J Med. 2019/01/17 ed. 2019 Jan 17;380(3):215–28.

78. Leang R, Barrette A, Bouth DM, Menard D, Abdur R, Duong S, et al. Efficacy of dihydroartemisinin-piperaquine for treatment of uncomplicated Plasmodium falciparum and Plasmodium vivax in Cambodia, 2008 to 2010. Antimicrob Agents Chemother. 2012/12/05 ed. 2013 Feb;57(2):818–26.

79. Leang R, Khim N, Chea H, Huy R, Mairet-Khedim M, Mey Bouth D, et al. Efficacy and Safety of Pyronaridine-Artesunate plus Single-Dose Primaquine for the Treatment of Malaria in Western Cambodia. Antimicrob Agents Chemother. 2019 Oct;63(10):e01273-19.

80. Lee SW, Lee M, Lee DD, Kim C, Kim YJ, Kim JY, et al. Biological resistance of hydroxychloroquine for Plasmodium vivax malaria in the Republic of Korea. Am J Trop Med Hyg. 2009/10/10 ed. 2009 Oct;81(4):600–4.

81. Liang GL, Sun XD, Wang J, Zhang ZX. [Sensitivity of Plasmodium vivax to chloroquine in Laza City, Myanmar]. Zhongguo Ji Sheng Chong Xue Yu Ji Sheng Chong Bing Za Zhi. 2009/10/27 ed. 2009 Apr;27(2):175–6.

82. Liu H, Yang HL, Xu JW, Wang JZ, Nie RH, Li CF. Artemisinin-naphthoquine combination versus chloroquine-primaquine to treat vivax malaria: an open-label randomized and non-inferiority trial in Yunnan Province, China. Malar J. 2013/11/13 ed. 2013 Nov 11;12:409.

83. Liu H, Yang HL, Tang LH, Li XL, Huang F, Wang JZ, et al. Monitoring Plasmodium vivax chloroquine sensitivity along China-Myanmar border of Yunnan Province, China during 2008-2013. Malar J. 2014/09/17 ed. 2014 Sep 15;13:364.

84. Llanos-Cuentas A, Lacerda MV, Rueangweerayut R, Krudsood S, Gupta SK, Kochar SK, et al. Tafenoquine plus chloroquine for the treatment and relapse prevention of Plasmodium vivax malaria (DETECTIVE): a multicentre, double-blind, randomised, phase 2b dose-selection study. Lancet. 2014;383(9922):1049–58.

85. Llanos-Cuentas A, Lacerda MVG, Hien TT, Vélez ID, Namaik-larp C, Chu CS, et al. Tafenoquine versus Primaquine to Prevent Relapse of Plasmodium vivax Malaria. N Engl J Med. 2019;380(3):229–41.

86. Lo E, Nguyen J, Oo W, Hemming-Schroeder E, Zhou G, Yang Z, et al. Examining Plasmodium falciparum and P. vivax clearance subsequent to antimalarial drug treatment in the Myanmar-China border area based on quantitative real-time polymerase chain reaction. BMC Infect Dis. 2016/04/17 ed. 2016 Apr 16;16:154.

87. Lon C, Manning JE, Vanachayangkul P, So M, Sea D, Se Y, et al. Efficacy of two versus three-day regimens of dihydroartemisinin-piperaquine for uncomplicated malaria in military personnel in northern Cambodia: an open-label randomized trial. PLoS One. 2014/03/29 ed. 2014;9(3):e93138.

88. Mac Donald-Ottevanger MS, Adhin M, Jitan J, Bretas G, Vreden S. Primaquine double dose for 7 days is inferior to single-dose treatment for 14 days in preventing Plasmodium vivax recurrent episodes in Suriname. Infect Drug Resist. 2017 Dec;Volume 11:3–8.

89. Macareo L, Lwin KM, Cheah PY, Yuentrakul P, Miller RS, Nosten F. Triangular test design to evaluate tinidazole in the prevention of Plasmodium vivax relapse. Malar J. 2013/05/31 ed. 2013 May 29;12:173.

90. Machado RL, de Figuereido Filho AF, Calvosa VS, Figueredo MC, Nascimento JM, Povoa MM. Correlation between Plasmodium vivax variants in Belem, Para State, Brazil and symptoms and clearance of parasitaemia. Braz J Infect Dis. 2003/09/23 ed. 2003 Jun;7(3):175–7.

91. Maguire JD, Lacy MD, Sururi, Sismadi P, Krisin, Wiady I, et al. Chloroquine or sulfadoxine-pyrimethamine for the treatment of uncomplicated, Plasmodium falciparum malaria during an epidemic in Central Java, Indonesia. Ann Trop Med Parasitol. 2003/01/23 ed. 2002 Oct;96(7):655–68.

92. Maguire JD, Krisin, Marwoto Hariyani, Richie TL, Fryauff DJ, Baird JK. Mefloquine is highly efficacious against chloroquine-resistant Plasmodium vivax malaria and Plasmodium falciparum malaria in Papua, Indonesia. Clin Infect Dis. 2006;42(8):1067–72.

93. Manandhar S, Bhusal CL, Ghimire U, Singh SP, Karmacharya DB, Dixit SM. A study on relapse/re-infection rate of Plasmodium vivax malaria and identification of the predominant genotypes of P. vivax in two endemic districts of Nepal. Malar J. 2013/09/18 ed. 2013 Sep 16;12:324.

94. Maneeboonyang W, Lawpoolsri S, Puangsa-Art S, Yimsamran S, Thanyavanich N, Wuthisen P, et al. Directly observed therapy with primaquine to reduce the recurrence rate of Plasmodium vivax infection along the Thai-Myanmar border. Southeast Asian J Trop Med Public Health. 2011;42(1):9–18.

95. Mesa-Echeverry E, Niebles-Bolívar M, Tobón-Castaño A. Chloroquine–Primaquine Therapeutic Efficacy, Safety, and Plasma Levels in Patients with Uncomplicated Plasmodium vivax Malaria in a Colombian Pacific Region. Am J Trop Med Hyg. 2019 Jan 9;100(1):72–7.

96. Miahipour A, Keshavarz H, Heidari A, Raeisi A, Rezaeian M, Rezaei S. Assessment of the efficacy of 8 weeks of primaquine for the prevention of relapse in vivax malaria patients using SSCP-PCR and sequencing in South and South-East Iran, 2008-2011. Trans R Soc Trop Med Hyg. 2013/05/23 ed. 2013 Jul;107(7):420–6.

97. Mishra N, Srivastava B, Bharti RS, Rana R, Kaitholia K, Anvikar AR, et al. Monitoring the efficacy of antimalarial medicines in India via sentinel sites: Outcomes and risk factors for treatment failure. J Vector Borne Dis. 2016/06/30 ed. 2016 Apr;53(2):168–78.

98. Mohapatra MK, Padhiary KN, Mishra DP, Sethy G. Atypical manifestations of Plasmodium vivax malaria. Indian J Malariol. 2002;39(1–2):18–25.

99. Muhamad P, Ruengweerayut R, Chacharoenkul W, Rungsihirunra K, Na-Bangchang K. Monitoring of clinical efficacy and in vitro sensitivity of Plasmodium vivax to chloroquine in area along Thai Myanmar border during 2009-2010. Malar J. 2011;10(1):44–44.

100. Nandy A, Addy M, Maji AK, Bandyopadhyay AK. Monitoring the chloroquine sensitivity of Plasmodium vivax from Calcutta and Orissa, India. Ann Trop Med Parasitol. 2003/06/14 ed. 2003 Apr;97(3):215–20.

101. Nateghpour M, Sayedzadeh SA, Edrissian GH, Raeisi A, Jahantigh A, Motevalli-Haghi A, et al. Evaluation of Sensitivity of Plasmodium vivax to Chloroquine. Iran J Public Health. 2007;36(3):60–3.

102. Nateghpour M, Edrissian G, Torabi A, Raesi A, Motevalli-Haghi A, Abed-Khojasteh H, et al. Monitoring of Plasmodium vivax and Plasmodium falciparum response to chloroquine in Bandar-Abbas district, Hormozgan province, Iran. [Arabic]. Tehran Uni Med J. 2009;67(3):178–83.

103. Negreiros S, Farias S, Viana GM, Okoth SA, Chenet SM, de Souza TM, et al. Efficacy of Chloroquine and Primaquine for the Treatment of Uncomplicated Plasmodium vivax Malaria in Cruzeiro do Sul, Brazil. Am J Trop Med Hyg. 2016/11/04 ed. 2016 Nov 2;95(5):1061–8.

104. Nyunt MH, Han JH, Wang B, Aye KM, Aye KH, Lee SK, et al. Clinical and molecular surveillance of drug resistant vivax malaria in Myanmar (2009-2016). Malar J. 2017/03/17 ed. 2017 Mar 16;16(1):117.

105. Osorio L, Perez Ldel P, Gonzalez IJ. [Assessment of the efficacy of antimalarial drugs in Tarapaca, in the Colombian Amazon basin]. Biomedica. 2007/06/05 ed. 2007 Mar;27(1):133–40.

106. Pareek A, Chandurkar N, Gogtay N, Deshpande A, Kakrani A, Kaneria M, et al. Sustained Release Formulation of Primaquine for Prevention of Relapse of Plasmodium vivax Malaria: A Randomized, Double-Blind, Comparative, Multicentric Study. Malar Res Treat. 2015/09/15 ed. 2015;2015:579864.

107. Pedro RS, Guaraldo L, Campos DP, Costa AP, Daniel-Ribeiro CT, Brasil P. Plasmodium vivax malaria relapses at a travel medicine centre in Rio de Janeiro, a non-endemic area in Brazil. Malar J. 2012/07/31 ed. 2012 Jul 28;11:245.

108. Perez MA, Cortes LJ, Guerra AP, Knudson A, Usta C, Nicholls RS. [Efficacy of the amodiaquine+sulfadoxine-pyrimethamine combination and of chloroquine for the treatment of malaria in Cordoba, Colombia, 2006]. Biomedica. 2008/07/23 ed. 2008 Mar;28(1):148–59.

109. Pham TV, Nguyen HV, Aguirre AR, Nguyen VV, M AC, Nguyen XX, et al. Plasmodium vivax morbidity after radical cure: A cohort study in Central Vietnam. PLoS Med. 20190517th ed. 2019 May;16(5):e1002784.

110. Phong NC, Chavchich M, Quang HH, San NN, Birrell GW, Chuang I, et al. Susceptibility of Plasmodium falciparum to artemisinins and Plasmodium vivax to chloroquine in Phuoc Chien Commune, Ninh Thuan Province, south-central Vietnam. Malar J. 2019 Dec;18(1):10.

111. Pinto AY, Azevedo CH, da Silva JB, de Souza JM. Assessment of chloroquine single dose treatment of malaria due to Plasmodium vivax in Brazilian Amazon. Rev Inst Med Trop Sao Paulo. 2004/02/06 ed. 2003 Nov;45(6):327–31.

112. Popovici J, Pierce-Friedrich L, Kim S, Bin S, Run V, Lek D, et al. Recrudescence, Reinfection, or Relapse? A More Rigorous Framework to Assess Chloroquine Efficacy for *Plasmodium vivax* Malaria. J Infect Dis. 2019 Jan 7;219(2):315–22.

113. Popovici J, Vantaux A, Primault L, Samreth R, Piv EP, Bin S, et al. Therapeutic and Transmission-Blocking Efficacy of Dihydroartemisinin/Piperaquine and Chloroquine against *Plasmodium vivax* Malaria, Cambodia. Emerg Infect Dis. 2018 Aug;24(8):1516–9.

114. Pukrittayakamee S, Chantra A, Simpson JA, Vanijanonta S, Clemens R, Looareesuwan S, et al. Therapeutic responses to different antimalarial drugs in vivax malaria. Antimicrob Agents Chemother. 2000;44(6):1680–5.

115. Rajgor DD, Gogtay NJ, Kadam VS, Kamtekar KD, Dalvi SS, Chogle AR, et al. Efficacy of a 14-day primaquine regimen in preventing relapses in patients with Plasmodium vivax malaria in Mumbai, India. Trans R Soc Trop Med Hyg. 2004/07/21 ed. 2003 Jul;97(4):438–40.

116. Rajgor DD, Gogtay NJ, Kadam VS, Kocharekar MM, Parulekar MS, Dalvi SS, et al. Antirelapse Efficacy of Various Primaquine Regimens for Plasmodium vivax. Malar Res Treat. 2014/10/09 ed. 2014;2014:347018.

117. Rios A, Alvarez G, Blair S. [Ten years of chloroquine efficacy for uncomplicated Plasmodium vivax malaria treatment, Turbo, Antioquia, 2002 and 2011]. Biomedica. 2014/03/22 ed. 2013 Jul;33(3):429–38.

118. Rogers WO, Sem R, Tero T, Chim P, Lim P, Muth S, et al. Failure of artesunate-mefloquine combination therapy for uncomplicated Plasmodium falciparum malaria in Southern Cambodia. Malar J. 2009;8:10–10.

119. Ruebush 2nd TK, Zegarra J, Cairo J, Andersen EM, Green M, Pillai DR, et al. Chloroquine-resistant Plasmodium vivax malaria in Peru. Am J Trop Med Hyg. 2003;69(5):548–52.

120. Saravu K, Acharya V, Kumar K, Kumar R. Plasmodium vivax remains responsive to chloroquine with primaquine treatment regimen: a prospective cohort study from tertiary care teaching hospital in southern India. Trop Doct. 2012/04/21 ed. 2012 Jul;42(3):163–4.

121. Seifu S, Zeynudin A, Zemene E, Suleman S, Biruksew A. Therapeutic efficacy of chloroquine for the treatment of Plasmodium vivax malaria among outpatients at Shawa Robit Health Care Centre, North-East Ethiopia. Acta Trop. 2017/03/17 ed. 2017 Jul;171:44–51.

122. Senn N, Rarau P, Manong D, Salib M, Siba P, Reeder JC, et al. Effectiveness of artemether/lumefantrine for the treatment of uncomplicated Plasmodium vivax and P. falciparum malaria in young children in Papua New Guinea. Clin Infect Dis. 2013/02/14 ed. 2013 May;56(10):1413–20.

123. Shaikh S, Memon S, Das C. Efficacy of oral chloroquine in uncomplicated vivax malaria in children. Med Forum Mon. 2017;28(4):148–51.

124. Shaikh S, Ahmed I, Memon SM, Saleem A, Memon H, Babar A. Therapeutic efficacy and safety of Dihydroartemisinin-piperaquine (DP) for the treatment of uncomplicated Plasmodium vivax malaria: A single center study. J Liaquat Uni Med Health Sci. 2017;16(2):93–8.

125. Shalini S, Chaudhuri S, Sutton PL, Mishra N, Srivastava N, David JK, et al. Chloroquine efficacy studies confirm drug susceptibility of Plasmodium vivax in Chennai, India. Malar J. 2014/04/02 ed. 2014 Mar 31;13:129.

126. Shumbej T, Jemal A, Worku A, Bekele F, Weldesenbet H. Therapeutic efficacy of chloroquine for treatment of Plasmodium vivax malaria cases in Guragae zone southern Central Ethiopia. BMC Infect Dis. 2019 Dec;19(1):413.

127. Singh RK. Emergence of chloroquine-resistant vivax malaria in South Bihar (India). Trans R Soc Trop Med Hyg. 2000;94:327–327.

128. Solari Soto L, Soto Tarazona AR, Mendoza Requena D, Llanos Cuentas EA. Ensayo clinico del tratamiento de la malaria vivax con esquema acortado de primaquina comparado con el esquema tradicional. Rev Soc Peru Med Interna. 2002;15(4):197–9.

129. Soto J, Toledo J, Gutierrez P, Luzz M, Llinas N, Cedeno N, et al. Plasmodium vivax clinically resistant to chloroquine in Columbia. Am J Trop Med Hyg. 2001;65:90–3.

130. Srivastava HC, Yadav RS, Joshi H, Valecha N, Mallick PK, Prajapati SK, et al. Therapeutic responses of Plasmodium vivax and P. falciparum to chloroquine, in an area of Western India where P. vivax predominates. Ann Trop Med Parasitol. 2008;102(6):471–80.

131. Sumawinata IW, Bernadeta, Leksana B, Sutamihardja A, Purnomo, Subianto B, et al. Very high risk of therapeutic failure with chloroquine for uncomplicated Plasmodium falciparum and P. vivax malaria in Indonesian Papua. Am J Trop Med Hyg. 2003;68(4):416–20.

132. Takeuchi R, Lawpoolsri S, Imwong M, Kobayashi J, Kaewkungwal J, Pukrittayakamee S, et al. Directly-observed therapy (DOT) for the radical 14-day primaquine treatment of Plasmodium vivax malaria on the Thai-Myanmar border. Malar J. 2010;9(1):308–308.

133. Tasanor O, Ruengweerayut R, Sirichaisinthop J, Congpuong K, Wernsdorfer WH, Na-Bangchang K. Clinical-parasitological response and in-vitro sensitivity of Plasmodium vivax to chloroquine and quinine on the western border of Thailand. Trans R Soc Trop Med Hyg. 2006/02/25 ed. 2006 May;100(5):410–8.

134. Taylor WR, Doan HN, Nguyen DT, Tran TU, Fryauff DJ, Gomez-Saladin E, et al. Assessing drug sensitivity of Plasmodium vivax to halofantrine or choroquine in southern, central Vietnam using an extended 28-day in vivo test and polymerase chain reaction genotyping. Am J Trop Med Hyg. 2001/04/17 ed. 2000 Jun;62(6):693–7.

135. Teka H, Petros B, Yamuah L, Tesfaye G, Elhassan I, Muchohi S, et al. Chloroquine-resistant Plasmodium vivax malaria in Debre Zeit, Ethiopia. Malar J. 2008;7:220–220.

136. Tjitra E, Baker J, Suprianto S, Cheng Q, Anstey NM. Therapeutic efficacies of artesunate-sulfadoxine-pyrimethamine and chloroquine-sulfadoxine-pyrimethamine in vivax malaria pilot studies: relationship to Plasmodium vivax dhfr mutations. Antimicrob Agents Chemother. 2002;46:3947–53.

137. Tjitra E, Hasugian AR, Siswantoro H, Prasetyorini B, Ekowatiningsih R, Yusnita EA, et al. Efficacy and safety of artemisinin-naphthoquine versus dihydroartemisinin-piperaquine in adult patients with uncomplicated malaria: a multi-centre study in Indonesia. Malar J. 2012/05/05 ed. 2012 Jun 14;11:153.

138. Valecha N, Savargaonkar D, Srivastava B, Rao BH, Tripathi SK, Gogtay N, et al. Comparison of the safety and efficacy of fixed-dose combination of arterolane maleate and piperaquine phosphate with chloroquine in acute, uncomplicated Plasmodium vivax malaria: a phase III, multicentric, open-label study. Malar J. 2016/01/29 ed. 2016 Jan 27;15:42.

139. Valibayov A, Abdullayev F, Mammadov S, Gasimov E, Sabatinelli G, Kondrachine AV, et al. Clinical efficacy of chloroquine followed by primaquine for Plasmodium vivax treatement in Azerbaijan. Acta Trop. 2003;88:99–102.

140. Van den Eede P, Soto-Calle VE, Delgado C, Gamboa D, Grande T, Rodriguez H, et al. Plasmodium vivax sub-patent infections after radical treatment are common in Peruvian patients: results of a 1-year prospective cohort study. PLoS One. 2011/02/08 ed. 2011 Jan 28;6(1):e16257.

141. Vijaykadga S, Rojanawatsirivej C, Congpoung K, Wilairatana P, Satimai W, Uaekowitchai C, et al. Assessment of therapeutic efficacy of chloroquine for vivax malaria in Thailand. Southeast Asian J Trop Med Public Health. 2005/02/04 ed. 2004 Sep;35(3):566–9.

142. Villalobos-Salcedo JM, Tada MS, Kimura E, Menezes MJ, Pereira da Silva LH. In-vivo sensitivity of Plasmodium vivax isolates from Rond nia (western Amazon region, Brazil) to regimens including chloroquine and primaquine. Ann Trop Med Parasitol. 2001/02/24 ed. 2000 Dec;94(8):749–58.

143. Walsh DS, Wilairatana P, Tang DB, Heppner DG Jr, Brewer TG, Krudsood S, et al. Randomized trial of 3-dose regimens of tafenoquine (WR238605) versus low-dose primaquine for preventing Plasmodium vivax malaria relapse. Clin Infect Dis. 2004/10/16 ed. 2004 Oct 15;39(8):1095–103.

144. Waqar T, Khushdil A, Haque K. Efficacy of Chloroquine as a first line agent in the treatment of uncomplicated malaria due to Plasmodium vivax in children and treatment practices in Pakistan: A Pilot study. J Pak Med Assoc. 2015/12/30 ed. 2016 Jan;66(1):30–3.

145. Yadav RS, Ghosh SK. Radical curative efficacy of five-day regimen of primaquine for treatment of Plasmodium vivax malaria in India. J Parasitol. 2002/11/19 ed. 2002 Oct;88(5):1042–4.

146. Yeshiwondim AK, Tekle AH, Dengela DO, Yohannes AM, Teklehaimanot A. Therapeutic efficacy of chloroquine and chloroquine plus primaquine for the treatment of Plasmodium vivax in Ethiopia. Acta Trop. 2010;113(2):105–13.

147. Yohannes AM, Teklehaimanot A, Bergqvist Y, Ringwald P. Confirmed vivax resistance to chloroquine and effectiveness of artemether-lumefantrine for the treatment of vivax malaria in Ethiopia. Am J Trop Med Hyg. 2011;84(1):137–40.

148. Zhu G, Lu F, Cao J, Zhou H, Liu Y, Han ET, et al. Blood stage of Plasmodium vivax in central China is still susceptible to chloroquine plus primaquine combination therapy. Am J Trop Med Hyg. 2013/05/15 ed. 2013 Jul;89(1):184–7.

149. Anez A, Moscoso M, Laguna A, Garnica C, Melgar V, Cuba M, et al. Resistance of infection by Plasmodium vivax to chloroquine in Bolivia. Malar J. 2015/07/02 ed. 2015 Jul 1;14:261.

150. Carmona-Fonseca J, Uscategui RM, Correa AM. Vivax malaria in children: Clinical features and response to chloroquine. Colomb Medica. 2008;39(4):364–77.

151. Daher A, Pereira D, Lacerda MVG, Alexandre MAA, Nascimento CT, Alves de Lima ESJC, et al. Efficacy and safety of artemisinin-based combination therapy and chloroquine with concomitant primaquine to treat Plasmodium vivax malaria in Brazil: an open label randomized clinical trial. Malar J. 2018/01/25 ed. 2018 Jan 24;17(1):45.

152. de Sena LWP, Mello A, Ferreira MVD, de Ataide MA, Dias RM, Vieira JLF. Doses of chloroquine in the treatment of malaria by Plasmodium vivax in patients between 2 and 14 years of age from the Brazilian Amazon basin. Malar J. 20191221st ed. 2019 Dec 21;18(1):439.

153. Gomes Mdo S, Vieira JL, Machado RL, Nacher M, Stefani A, Musset L, et al. Efficacy in the treatment of malaria by Plasmodium vivax in Oiapoque, Brazil, on the border with French Guiana: the importance of control over external factors. Malar J. 2015/10/11 ed. 2015 Oct 9;14:402.

154. Leslie T, Rab MA, Ahmadzai H, Durrani N, Fayaz M, Kolaczinski J, et al. Compliance with 14-day primaquine therapy for radical cure of vivax malaria: a randomized placebo-controlled trial comparing unsupervised with supervised treatment. Trans R Soc Trop Med Hyg. 2004;98:168–73.

155. Leslie T, Mayan I, Mohammed N, Erasmus P, Kolaczinski J, Whitty CJ, et al. A randomised trial of an eight-week, once weekly primaquine regimen to prevent relapse of Plasmodium vivax in Northwest Frontier Province, Pakistan. PLoS ONE. 2008;3(8):e2861–e2861.

156. Nelwan EJ, Ekawati LL, Tjahjono B, Setiabudy R, Sutanto I, Chand K, et al. Randomized trial of primaquine hypnozoitocidal efficacy when administered with artemisinin-combined blood schizontocides for radical cure of Plasmodium vivax in Indonesia. BMC Med [Internet]. 2015;13(1). Available from: https://bmcmedicine.biomedcentral.com/articles/10.1186/s12916-015-0535-9

157. Orjuela-Sanchez P, da Silva NS, da Silva-Nunes M, Ferreira MU. Recurrent parasitemias and population dynamics of Plasmodium vivax polymorphisms in rural Amazonia. Am J Trop Med Hyg. 2009 Dec;81(6):961–8.

158. Pukrittayakamee S, Imwong M, Chotivanich K, Singhasivanon P, Day NP, White NJ. A comparison of two short-course primaquine regimens for the treatment and radical cure of Plasmodium vivax malaria in Thailand. Am J Trop Med Hyg. 2010;82(4):542–7.

159. Saravu K, Tellapragada C, Kulavalli S, Xavier W, Umakanth S, Brahmarouphu G, et al. A pilot randomized controlled trial to compare the effectiveness of two 14-day primaquine regimens for the radical cure of vivax malaria in South India. Malar J. 2018/09/05 ed. 2018 Sep 3;17(1):321.

160. Sutanto I, Tjahjono B, Basri H, Taylor WR, Putri FA, Meilia RA, et al. Randomized, open-label trial of primaquine against vivax malaria relapse in Indonesia. Antimicrob Agents Chemother. 2013;57(3):1128–35.

161. Alvarez G, Pineros JG, Tobon A, Rios A, Maestre A, Blair S, et al. Efficacy of three chloroquine-primaquine regimens for treatment of Plasmodium vivax malaria in Colombia. Am J Trop Med Hyg. 2006;75(4):605–9.

162. Abdallah TM, Ali AA, Bakri M, Gasim GI, Musa IR, Adam I. Efficacy of artemether-lumefantrine as a treatment for uncomplicated Plasmodium vivax malaria in eastern Sudan. Malar J. 2012/12/12 ed. 2012 Dec 5;11:404.

163. Abreha T, Hwang J, Thriemer K, Tadesse Y, Girma S, Melaku Z, et al. Comparison of artemether-lumefantrine and chloroquine with and without primaquine for the treatment of Plasmodium vivax infection in Ethiopia: A randomized controlled trial. PLoS Med. 2017;14(5):1–17.

164. Awab GR, Pukrittayakamee S, Imwong M, Dondorp AM, Woodrow CJ, Lee SJ, et al. Dihydroartemisinin-piperaquine versus chloroquine to treat vivax malaria in Afghanistan: an open randomized, non-inferiority, trial. Malar J. 2010;9(1):105–105.

165. Barber BE, William T, Grigg MJ, Menon J, Auburn S, Marfurt J, et al. A prospective comparative study of knowlesi, falciparum, and vivax malaria in Sabah, Malaysia: high proportion with severe disease from Plasmodium knowlesi and Plasmodium vivax but no mortality with early referral and artesunate therapy. Clin Infect Dis. 2013 Feb;56(3):383–97.

166. Chu CS, Phyo AP, Lwin KM, Win HH, San T, Aung AA, et al. Comparison of the Cumulative Efficacy and Safety of Chloroquine, Artesunate, and Chloroquine-Primaquine in Plasmodium vivax Malaria. Clin Infect Dis [Internet]. 2018/06/12 ed. 2018 Jun 8; Available from: https://www.ncbi.nlm.nih.gov/pubmed/29889239

167. Chu CS, Phyo AP, Turner C, Win HH, Poe NP, Yotyingaphiram W, et al. Chloroquine Versus Dihydroartemisinin-Piperaquine With Standard High-dose Primaquine Given Either for 7 Days or 14 Days in Plasmodium vivax Malaria. Clin Infect Dis. 24 August 2018. 2019;68(8):1311–9.

168. Dao NVH, Cuong BT, Ngoa ND, Thuy LTT, The ND, Duy DN, et al. Vivax malaria: preliminary observations following a shorter course of treatment with artesunate plus primaquine. Trans R Soc Trop Med Hyg. 2007;101(6):534–9.

169. Getachew S, Thriemer K, Auburn S, Abera A, Gadisa E, Aseffa A, et al. Chloroquine efficacy for Plasmodium vivax malaria treatment in southern Ethiopia. Malar J. 2015/12/26 ed. 2015 Dec 24;14:525.

170. Gonzalez-Ceron L, Rodriguez MH, Sandoval MA, Santillan F, Galindo-Virgen S, Betanzos AF, et al. Effectiveness of combined chloroquine and primaquine treatment in 14 days versus intermittent single dose regimen, in an open, non-randomized, clinical trial, to eliminate Plasmodium vivax in southern Mexico. Malar J. 2015/11/01 ed. 2015 Oct 30;14:426.

171. Grigg MJ, William T, Menon J, Barber BE, Wilkes CS, Rajahram GS, et al. Efficacy of Artesunate-mefloquine for Chloroquine-resistant Plasmodium vivax Malaria in Malaysia: An Open-label, Randomized, Controlled Trial. Clin Infect Dis. 2016 Jun 1;62(11):1403–11.

172. Guthmann JP, Pittet A, Lesage A, Imwong M, Lindegardh N, Min Lwin M, et al. Plasmodium vivax resistance to chloroquine in Dawei, Southern Myanmar. Trop Med Int Health. 2008;13(1):91–8.

173. Hasugian AR, Purba HLE, Kenangalem E, Wuwung RM, Ebsworth EP, Maristela R, et al. Dihydroartemisinin-piperaquine versus artesunate-amodiaquine: superior efficacy and posttreatment prophylaxis against multidrug-resistant Plasmodium falciparum and Plasmodium vivax malaria. Clin Infect Dis. 2007;44:1067–74.

174. Hasugian AR, Tjitra E, Ratcliff A, Siswantoro H, Kenangalem E, Wuwung RM, et al. In vivo and in vitro efficacy of amodiaquine monotherapy for treatment of infection by chloroquine-resistant Plasmodium vivax. Antimicrob Agents Chemother. 2009;53(3):1094–9.

175. Heidari A, Keshavarz H, Shojaee S, Raeisi A, Dittrich S. In vivo Susceptibility of Plasmodium vivax to Chloroquine in Southeastern Iran. Iran J Parasitol. 2012/10/31 ed. 2012;7(2):8–14.

176. Hwang J, Alemayehu BH, Reithinger R, Tekleyohannes SG, Takele T, Birhanu SG, et al. In vivo efficacy of artemether-lumefantrine and chloroquine against Plasmodium vivax: a randomized open label trial in central Ethiopia. PLoS One. 2013;8(5):e63433.

177. Karunajeewa HA, Mueller I, Senn M, Lin E, Law I, Gomorrai PS, et al. A trial of combination antimalarial therapies in children from Papua New Guinea. N Engl J Med. 2008;359:2545–57.

178. Ketema T, Bacha K, Birhanu T, Petros B. Chloroquine-resistant Plasmodium vivax malaria in Serbo town, Jimma zone, South-west Ethiopia. Malar J. 2009;8:177–177.

179. Ladeia-Andrade S, Ferreira MarceloU, de Carvalho ME, Curado I, Coura JR. Age-dependent acquisition of protective immunity to malaria in riverine populations of the Amazon Basin of Brazil. Am J Trop Med Hyg. 2009;80(3):452–9.

180. Laman M, Moore BR, Benjamin JM, Yadi G, Bona C, Warrel J, et al. Artemisinin-naphthoquine versus artemether-lumefantrine for uncomplicated malaria in Papua New Guinean children: an open-label randomized trial. PLoS Med. 2014/12/31 ed. 2014 Dec;11(12):e1001773.

181. Leslie T, Mayan MI, Hasan MA, Safi MH, Klinkenberg E, Whitty CJ, et al. Sulfadoxine-pyrimethamine, chlorproguanil-dapsone, or chloroquine for the treatment of Plasmodium vivax malaria in Afghanistan and Pakistan: a randomized controlled trial. JAMA. 2007 May 23;297(20):2201–9.

182. Ley B, Alam MS, Thriemer K, Hossain MS, Kibria MG, Auburn S, et al. G6PD Deficiency and Antimalarial Efficacy for Uncomplicated Malaria in Bangladesh: A Prospective Observational Study. PLoS One. 2016/04/30 ed. 2016;11(4):e0154015.

183. Lidia K, Dwiprahasto I, Kristin E. Therapeutic effects of dyhidroartemisinin piperaquine versus chloroquine for uncomplicated Vivax Malaria in Kupang, East Nusa Tenggara, Indonesia. Int J Pharm Sci Rev Res. 2015;31(2):247–51.

184. Longley RJ, Sripoorote P, Chobson P, Saeseu T, Sukasem C, Phuanukoonnon S, et al. High Efficacy of Primaquine Treatment for Plasmodium vivax in Western Thailand. Am J Trop Med Hyg. 2016/11/04 ed. 2016 Nov 2;95(5):1086–9.

185. Marques MM, Costa MR, Santana Filho FS, Vieira JL, Nascimento MT, Brasil LW, et al. Plasmodium vivax chloroquine resistance and anemia in the western Brazilian Amazon. Antimicrob Agents Chemother. 2013/10/30 ed. 2014;58(1):342–7.

186. Mishra N, Singh JP, Srivastava B, Arora U, Shah NK, Ghosh SK, et al. Monitoring antimalarial drug resistance in India via sentinel sites: outcomes and risk factors for treatment failure, 2009-2010. Bull World Health Organ. 2013/01/04 ed. 2012 Dec 1;90(12):895–904.

187. Pasaribu AP, Chokejindachai W, Sirivichayakul C, Tanomsing N, Chavez I, Tjitra E, et al. A randomized comparison of dihydroartemisinin-piperaquine and artesunate-amodiaquine combined with primaquine for radical treatment of vivax malaria in Sumatera, Indonesia. J Infect Dis. 2013/08/09 ed. 2013 Dec 1;208(11):1906–13.

188. Pereira D, Daher A, Zanini G, Maia I, Fonseca L, Pitta L, et al. Safety, efficacy and pharmacokinetic evaluations of a new coated chloroquine tablet in a single-arm open-label non-comparative trial in Brazil: a step towards a user-friendly malaria vivax treatment. Malar J. 2016/09/19 ed. 2016 Sep 17;15:477.

189. Phan GT, de Vries PJ, Tran BQ, Le HQ, Nguyen NV, Nguyen TV, et al. Artemisinin or chloroquine for blood stage Plasmodium vivax malaria in Vietnam. Trop Med Int Health. 2002;7(10):858–64.

190. Phyo AP, Lwin KM, Ashley EA, Price RN, Russell B, Sriprawat K, et al. Dihydroartemisinin-piperaquine versus chloroquine in the treatment of P. vivax malaria in Thailand: a randomized controlled trial. Clin Infect Dis. 2011;53:977–84.

191. Poespoprodjo JR, Kenangalem E, Wafom J, Chandrawati F, Puspitasari AM, Ley B, et al. Therapeutic Response to Dihydroartemisinin-Piperaquine for P. falciparum and P. vivax Nine Years after Its Introduction in Southern Papua, Indonesia. Am J Trop Med Hyg. 2018/01/19 ed. 2018 Mar;98(3):677–82.

192. Poravuth Y, Socheat D, Rueangweerayut R, Uthaisin C, Phyo AP, Valecha N, et al. Pyronaridine-artesunate versus chloroquine in patients with acute Plasmodium vivax malaria: a randomized, double-blind, non-inferiority trial. PLoS ONE. 2011;6(1):e14501–e14501.

193. Ratcliff A, Siswantoro H, Kenangalem E, Wuwung M, Brockman A, Edstein MD, et al. Therapeutic response of multidrug-resistant Plasmodium falciparum and P. vivax to chloroquine and sulfadoxine-pyrimethamine in Southern Papua, Indonesia. Trans R Soc Trop Med Hyg. 2007;101:351–9.

194. Ratcliff A, Siswantoro H, Kenangalem E, Maristela R, Wuwung M, Laihad F, et al. Two fixed-dose artemisinin combinations for drug-resistant falciparum and vivax malaria in Papua, Indonesia: an open-label randomised comparison. Lancet. 2007;369:757–65.

195. Rijal KR, Adhikari B, Ghimire P, Banjara MR, Das Thakur G, Hanboonkunupakarn B, et al. Efficacy of Primaquine in Preventing Short- and Long-Latency Plasmodium vivax Relapses in Nepal. J Infect Dis. 2019/03/19 ed. 2019 Jul 2;220(3):448–56.

196. Rishikesh K, Kamath A, Hande MH, Vidyasagar S, Acharya RV, Acharya V, et al. Therapeutic assessment of chloroquine-primaquine combined regimen in adult cohort of Plasmodium vivax malaria from a tertiary care hospital in southwestern India. Malar J. 2015/08/12 ed. 2015 Aug 11;14:310.

197. Saravu K, Kumar R, Ashok H, Kundapura P, Kamath V, Kamath A, et al. Therapeutic Assessment of Chloroquine-Primaquine Combined Regimen in Adult Cohort of Plasmodium vivax Malaria from Primary Care Centres in Southwestern India. PLoS One. 2016/06/18 ed. 2016;11(6):e0157666.

198. Siqueira AM, Alencar AC, Melo GC, Magalhaes BL, Machado K, Alencar Filho AC, et al. Fixed-Dose Artesunate-Amodiaquine Combination vs Chloroquine for Treatment of Uncomplicated Blood Stage P. vivax Infection in the Brazilian Amazon: An Open-Label Randomized, Controlled Trial. Clin Infect Dis. 2016/12/19 ed. 2017 Jan 15;64(2):166–74.

199. Sutanto I, Suprijanto S, Nurhayati, Manoempil P, Baird JK. Resistance to chloroquine by Plasmodium vivax at Alor in the Lesser Sundas Archipelago in Eastern Indonesia. Am J Trop Med Hyg. 2009;81(2):338–42.

200. Sutanto I, Endawati D, Ling LH, Laihad F, Setiabudy R, Baird JK. Evaluation of chloroquine therapy for vivax and falciparum malaria in southern Sumatra, western Indonesia. Malar J. 2010 Feb 12;9:52.

201. Taylor WR, Widjaja H, Richie TL, Basri H, Ohrt C, Tjitra, et al. Chloroquine/doxycycline combination versus chloroquine alone, and doxycycline alone for the treatment of Plasmodium falciparum and Plasmodium vivax malaria in Northeastern Irian Jaya, Indonesia. Am J Trop Med Hyg. 2001;64(5–6):223–8.

202. Taylor WRJ, Thriemer K, von Seidlein L, Yuentrakul P, Assawariyathipat T, Assefa A, et al. Short-course primaquine for the radical cure of Plasmodium vivax malaria: a multicentre, randomised, placebo-controlled non-inferiority trial. The Lancet. 2019;394(10202):929–38.

203. Thanh PV, Hong NV, Van NV, Louisa M, Baird K, Xa NX, et al. Confirmed Plasmodium vivax Resistance to Chloroquine in Central Vietnam. Antimicrob Agents Chemother. 2015 Dec;59(12):7411–9.

204. Thuan PD, Ca NT, Van Toi P, Nhien NT, Thanh NV, Anh ND, et al. A Randomized Comparison of Chloroquine Versus Dihydroartemisinin-Piperaquine for the Treatment of Plasmodium vivax Infection in Vietnam. Am J Trop Med Hyg. 2016/02/10 ed. 2016 Apr;94(4):879–85.

205. Valecha N, Joshi H, Eapen A, Ravinderan J, Kumar A, Prajapati SK, et al. Therapeutic efficacy of chloroquine in Plasmodium vivax from areas with different epidemiological patterns in India and their Pvdhfr gene mutation pattern. Trans R Soc Trop Med Hyg. 2006/03/04 ed. 2006 Sep;100(9):831–7.

206. Wangchuk S, Drukpa T, Penjor K, Peldon T, Dorjey Y, Dorji K, et al. Where chloroquine still works: the genetic make-up and susceptibility of Plasmodium vivax to chloroquine plus primaquine in Bhutan. Malar J. 2016/05/14 ed. 2016 May 12;15(1):277.

207. Xu S, Zeng W, Ngassa Mbenda HG, Liu H, Chen X, Xiang Z, et al. Efficacy of directly-observed chloroquine-primaquine treatment for uncomplicated acute Plasmodium vivax malaria in northeast Myanmar: A prospective open-label efficacy trial. Travel Med Infect Dis. 20191008th ed. 2020 Jul;36:101499.

208. Yuan L, Wang Y, Parker DM, Gupta B, Yang Z, Liu H, et al. Therapeutic responses of Plasmodium vivax malaria to chloroquine and primaquine treatment in northeastern Myanmar. Antimicrob Agents Chemother. 2015 Feb;59(2):1230–5.

209. Zuluaga-Idarraga L, Blair S, Akinyi Okoth S, Udhayakumar V, Marcet PL, Escalante AA, et al. Prospective Study of Plasmodium vivax Malaria Recurrence after Radical Treatment with a Chloroquine-Primaquine Standard Regimen in Turbo, Colombia. Antimicrob Agents Chemother. 2016/05/18 ed. 2016 Aug;60(8):4610–9.

210. Battle KE, Karhunen MS, Bhatt S, Gething PW, Howes RE, Golding N, et al. Geographical variation in Plasmodium vivax relapse. Malar J. 2014 Apr 15;13:144.
