# Supplementary material for: Parasitaemia and fever in uncomplicated Plasmodium vivax malaria: A systematic review and individual patient data meta-analysis
Source: PLoS Negl Trop Dis. 2025 Mar 28;19(3):e0012951. doi: 10.1371/journal.pntd.0012951 (PMC11978046; doi:10.1371/journal.pntd.0012951)
Supplement: S1 Acknowledgments — (DOCX) [file pntd.0012951.s002.docx]

The WWARN P. vivax Fever Study Group comprises the named authors and the following additional authors, who we would like to thank and acknowledge:

Abraham Aseffa, Tesfay Abreha, Ishag Adam, Bipin Adhikari, Mohammad Shafiul Alam, Sisay Getachew Alemu, Nicholas M. Anstey, Ashenafi Assefa, Ghulam Rahim Awab, J. Kevin Baird, Bridget E. Barber, Isabelle Borghini-Fuhrer, Sarah C. Boyd, Nguyen Hoang Chau, Cindy S. Chu, Liwang Cui, Timothy M. E. Davis, Nicholas P. J. Day, Peter J. de Vries, Tamiru Shibiru Degaga, Arjen M. Dondorp, Nicholas M. Douglas, Michael D. Edstein, Annette Erhart , Marcelo U. Ferreira, Prakash Ghimire, Lilia Gonzalez‑Ceron, Matthew J. Grigg, Philippe J. Guerin, Mohammad Anwar Hasanzai, Aliehsan Heidari, Jimee Hwang, Piet A. Kager, Harin Karunajeewa, Tsige Ketema, Wasif Ali Khan, Marcus V. G. Lacerda, Simone Ladeia-Andrade, Moses Laman, Toby Leslie, Benedikt Ley, Kartini Lidia, Rhea J. Longley, Asrat Hailu Mekuria, Wuelton Marcelo Monteiro, Brioni R. Moore, Ivo Mueller, Mohammad Nader Naadim, Francois Nosten, Giao Trong Phan, Aung Pyae Phyo, Jeanne Rini Poespoprodjo, David J. Price, Sasithon Pukrittayakamee, Komal Raj Rijal, Mark Rowland, Jetsumon Sattabongkot, André M. Siqueira, Kasia Stepniewska, Inge Sutanto, Walter R. J. Taylor, Pham Vinh Thanh, Kamala Thriemer, Guy Thwaites, Binh Quang Tran, Neena Valecha, Sonam Wangchuk, Nicholas J. White, Timothy William, Charles J. Woodrow, Adugna Woyessa, Lina M. Zuluaga-Idarraga.
